# The Brain–Gut–Bone Axis in Neurodegenerative Diseases: Insights, Challenges, and Future Prospects

**DOI:** 10.1002/advs.202307971

**Published:** 2024-08-09

**Authors:** Rong Li, Zong Miao, Yu'e Liu, Xiao Chen, Hongxiang Wang, Jiacan Su, Juxiang Chen

**Affiliations:** ^1^ Department of Neurosurgery Shanghai Changhai Hospital Naval Medical University Shanghai 200433 China; ^2^ Tongji University Cancer Center Shanghai Tenth People's Hospital of Tongji University School of Medicine Tongji University Shanghai 200092 China; ^3^ Department of Orthopedics Xinhua Hospital Shanghai Jiao Tong University School of Medicine Shanghai 200092 China; ^4^ Institute of Translational Medicine Shanghai University Shanghai 200444 China; ^5^ Organoid Research Center Shanghai University Shanghai 200444 China

**Keywords:** brain–gut–bone axis, intestinal microbiota regulation, neurodegenerative diseases, physiological systems, skeletal system

## Abstract

Neurodegenerative diseases are global health challenges characterized by the progressive degeneration of nerve cells, leading to cognitive and motor impairments. The brain–gut–bone axis, a complex network that modulates multiple physiological systems, has gained increasing attention owing to its profound effects on the occurrence and development of neurodegenerative diseases. No comprehensive review has been conducted to clarify the triangular relationship involving the brain–gut–bone axis and its potential for innovative therapies for neurodegenerative disorders. In light of this, a new perspective is aimed to propose on the interplay between the brain, gut, and bone systems, highlighting the potential of their dynamic communication in neurodegenerative diseases, as they modulate multiple physiological systems, including the nervous, immune, endocrine, and metabolic systems. Therapeutic strategies for maintaining the balance of the axis, including brain health regulation, intestinal microbiota regulation, and improving skeletal health, are also explored. The intricate physiological interactions within the brain–gut–bone axis pose a challenge in the development of effective treatments that can comprehensively target this system. Furthermore, the safety of these treatments requires further evaluation. This review offers a novel insights and strategies for the prevention and treatment of neurodegenerative diseases, which have important implications for clinical practice and patient well‐being.

## Graphical abstract

This review offers a novel perspective on the brain–gut–bone axis, highlighting its dynamic communication and exploring its significance in neurodegenerative diseases. It proposes therapeutic strategies to preserve axis balance, including regulating brain health, intestinal flora, and improving skeletal well‐being. These findings have crucial implications for clinical practice and overall patient well‐being.

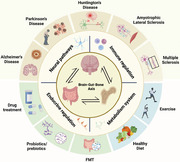

## 1 Introduction

Neurodegenerative diseases are characterized by dysfunctional neurons in the brain and spinal cord, they include Alzheimer's disease (AD), Parkinson's disease (PD), genetic degenerative diseases of the nervous system, such as Huntington's disease (HD), motor neuron diseases such as amyotrophic lateral sclerosis (ALS), progressive bulbar paralysis, and spinal muscular atrophy.^[^ ^1^ ^]^ Neurodegenerative diseases are among the dominant causes of disability and morbidity worldwide and have received considerable attention owing to their remarkable influence on an aging society. These diseases are primarily caused by the constant deterioration of neuronal function, which leads to brain atrophy.^[^ ^2^ ^]^ With an increase in the aging population, the number of neurodegenerative diseases is increasing annually. Currently, the pathogenesis of neurodegenerative diseases is not clear, and the available drugs only relieve symptoms but cannot reverse or prevent the loss of neurons.^[^ ^3^ ^]^ Therefore, it is of great practical significance to conduct research on neurodegenerative diseases, explore the mechanisms of disease occurrence and development, and find effective ways for early diagnosis, prevention, and treatment.

The role of the brain–gut axis in the management of neurodegenerative diseases has gained increasing attention in recent years.^[^ ^4^ ^]^ The brain–gut axis is a complex and bidirectional communication network between the central nervous system (CNS) and gut microbiota, which has been increasingly recognized to play a critical role in the pathogenesis of neurodegenerative diseases.^[^ ^5^ ^]^ Studies have shown that dysregulated commensal microbiota may contribute to the pathogenesis of neurodegenerative diseases such as AD and PD and induce chronic systemic inflammation, leading to neuroinflammation and neurodegeneration.^[^ ^6^ ^]^ The gut microbiota also produces metabolites, including neurotransmitters and neuropeptides, that can influence brain function and behavior.^[^ ^7^ ^]^

Extensive investigations have been conducted on the bidirectional communication between the CNS and skeletal system. The brain is regarded as the principal coordinator of body homeostasis because it regulates the activity of all body organs and their interplay, whereas bones have hematopoietic, endocrine, metabolic, and storage functions, in addition to their primary mechanical function.^[^ ^8^ ^]^ Numerous stress, mood, and neurodegenerative disorders of the brain are associated with bone loss.^[^ ^9^ ^]^

Interestingly, bone metabolism and homeostasis are regulated by the gut microbes.^[^ ^10^ ^]^ The gut microbiota plays an important role in nutrient absorption, immunomodulation, and the brain–gut–bone axis, and has been associated with bone diseases such as osteoporosis (OP).^[^ ^11^ ^]^ The gut microbiota also modulates the immune system and the enteric nervous system (ENS), which contributes to the development and progression of neurodegenerative diseases.^[^ ^12^ ^]^

Recent studies on systemic diseases such as hypertension and OP have introduced the concept of the brain–gut–bone marrow axis, which refers to the hypothesis that hematopoietic stem cells might migrate to the brain, gut, or bone, contributing to local inflammation and complex immune responses.^[^ ^13^ ^]^ The axis is described by multiple reactional signaling interactions between the gastrointestinal tract, bone, and CNS.

Recent research has shown that dysbiosis of the gut microbiota and skeletal system has profound effects on the occurrence and development of neurodegenerative diseases.^[^ ^4^, ^14^ ^]^ To date, no comprehensive review has been conducted to elucidate the triangular relationship involving the brain–gut–bone axis and its potential for innovative therapies in the management of neurodegenerative disorders.

**Figure 1:**
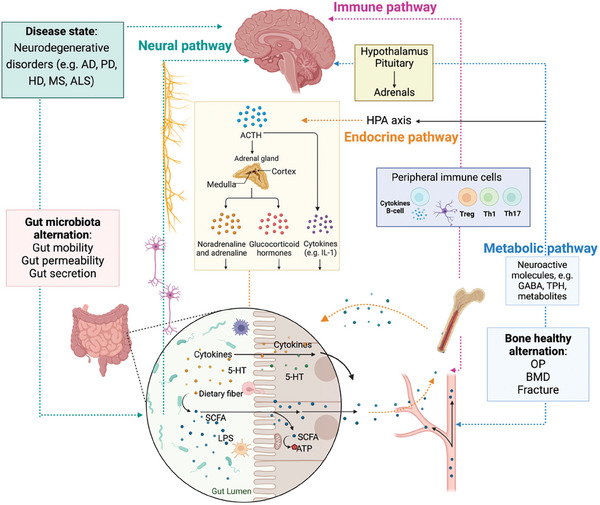
Schematic diagram of molecular communication pathways among the brain, microbiota, and the bone system via the brain–gut–bone axis. The brain–gut–bone axis is comprised of the neural pathways; the immune pathways; the endocrine pathways and microbial metabolites. The CNS and the ENS of the gut are connected through intermediate neural pathways such as the vagus nerve (VN) and/or spinal fibers. The brain primarily regulates brain‐derived molecules, including hormones, neuropeptides, and neurotransmitters, which depend on the sensory nerve supply acting on the skeleton. The gut microbiota participates in the regulation of neural function by modulating brain‐derived neuroactive molecules, including neurons, neuroendocrine factors, and neuroimmune factors. The gut microbial community influences skeletal metabolism balance through aspects such as intestinal barrier, metabolic pathways, nutrient absorption of calcium and phosphorus, immune system, and hormonal environment. The skeleton primarily regulates bone‐derived molecules, including hormones and peptides, which act on the brain. The gut and bone tissue interact through a complex network regulated by the gut microbiota. Figures were created with BioRender.com.

In light of this, our review aims to propose a hypothesis regarding the role of the brain–gut–bone axis in neurodegenerative diseases, which refers to the interconnected network of biological systems that allow dynamic communication between the brain, gut microbiota, and bone. As illustrated in **Figure** **1**, communication pathways in these biological networks involve neuronal pathways, the immune system, endocrine regulation, and metabolism system, which are crucial for maintaining the homeostasis of the gastrointestinal, central nervous, and skeletal systems. This review also explores therapeutic strategies for maintaining the balance of the brain–gut–bone axis, including the regulation of brain, intestinal microbiota, and skeletal health. Neurodegenerative treatments aim to maintain the balance in the brain–gut–bone axis by combining interventions such as regulating and reshaping the intestinal microbiota (using probiotics, prebiotics, and fecal microbiota transplant), enhancing the skeletal system through exercise, and integrating emerging therapeutic approaches. These strategies can positively affect the gut microbiota and slow down the progression of neurodegenerative diseases. Hence, the insights provided by this review shed light on the important role of the brain–gut–bone axis in the prevention and treatment of neurodegenerative diseases, which have significant implications for clinical practice.

## 2 Brain–Gut–Bone Axis: Interplay and Regulation

In recent years, exploring the interplay in the pathogenesis of neurodegenerative diseases and developing rational and effective intervention strategies have emerged as new research trends. The exploration of the gut microbiota has provided insights regarding the potential targets of the brain–gut–bone axis in neurodegenerative diseases that influence their onset and progression. Therefore, based on the concept of the brain–gut–bone axis, we primarily delved into and summarized the neurogenic, immune, endocrine, and microbial metabolic pathways to provide a novel perspective for prevention strategies and mechanistic research of neurodegenerative diseases.

### 2.1 Neural Pathways

#### 2.1.1 The Vagus Nerves (VNs) and Autonomic Nervous System (ANS) Play a Vital Role in Brain and Gut Communication

Neural pathways are the specific routes within the nervous system that transmit information. Regulation of the gut–brain axis relies primarily on the vagus nerve (VN). There is a significant presence of VN in the human digestive tract, which can perceive changes in microbial populations. These nerves integrate and transmit information from the gut to the CNS, resulting in adaptive or maladaptive responses. Maladaptive responses primarily manifest as gastrointestinal pathologies and neurodegenerative diseases.^[^ ^15^ ^]^ The brain and the gut communicate bidirectionally via the VN and ANS in the spinal cord, influencing each other's functions through intricate neurohumoral pathways. Signals from the brain alter sensorimotor and secretory functions in the gut via complex neurohumoral pathways.^[^ ^16^ ^]^ In addition, VN activation shows anti‐inflammatory effects, and VN activity positively influences the gut microbiota and beneficial bacteria. The VN transfers endocrine neurons and microbial changes from the gastrointestinal tract to the brain.^[^ ^17^ ^]^

The ANS, combined with the hypothalamic‐pituitary‐adrenal (HPA) axis, constitutes a large and complex comprehensive communication network between the brain and gut, which involuntarily establishes and regulates the physiological homeostasis of the host.^[^ ^18^ ^]^ The ANS responds to neuronal and neuroendocrine signals to induce CNS‐regulated intestinal changes (top‐down effect).^[^ ^19^ ^]^ Key gastrointestinal functions, including intestinal motility and permeability, epithelial fluid maintenance, intraventricular osmotic pressure, biliary secretion, carbohydrate levels, mucosal mechanical deformation, bicarbonate and mucus production, mucosal immune response, and intestinal fluid processing, are controlled by the ANS.^[^ ^20^ ^]^ In the ANS, components of the microbial–gut–brain axis communicate in antagonistic and synergistic ways.^[^ ^21^ ^]^

Visceral afferent signals originating from the gut also regulate brain function.^[^ ^16^ ^]^ Gut microbiota contributes to the development of enteric glial cells, which regulate gut homeostasis and maintain neuronal networks.^[^ ^22^ ^]^ Molecular compounds derived from microorganisms contribute to the modulation of the behavior of hosts and the function of their nervous systems. The neuronal pathways of the VN and the immune system are modified by molecules derived from microbes. These compounds can travel through portal circulation and directly affect the local pathways of the VN, providing signals to the brain via the ENS and afferent pathways of the VN.

Hence, the VN and ANS are essential components in the bidirectional communication between the brain and gut. The VN is the longest cranial nerve and plays a vital role in transmitting sensory and motor signals between the brain and various organs, including the gut. The ANS, which consists of the sympathetic and parasympathetic branches, regulates involuntary body functions, including digestion and gut motility. This bidirectional pathway allows the exchange of signals and information between the two systems.

#### 2.1.2 The Role of VN and ENS in the Brain and Gut System

VN is one of the most important neuronal pathways in the body, running from the brainstem to innervate the gut and ENS. Afferent pathways of the VN and the ENS can be stimulated by stress hormones, immune mediators, and CNS neurotransmitters, which alter the gut environment and microbiota.^[^ ^16^, ^22^ ^]^ Bacteria establish direct neural connections between the brain and gastrointestinal microbiota through stimulation of afferent neurons in the VN and ENS. The ENS is a plexus of internal nerves that is distributed in the wall of the gastrointestinal tube from the esophagus to the anus. The ENS is divided into two ganglion plexuses, the submucosal plexus and the myenteric plexus, which are mainly responsible for coordinating intestinal functions, such as motor and fluid movement control.^[^ ^23^ ^]^ A ganglion is composed of an interconnected nerve plexus or neural network with small groups of nerve cell bodies in the wall of the tube.^[^ ^24^ ^]^ The preganglionic fibers of the VN from the dorsal nucleus of the VN form synapses with motor neurons in the ENS. Postganglionic fibers from the spinal cord terminate directly in the muscular layer and blood vessels. Under physiological conditions, the ENS receives incoming signals from the central sympathetic and parasympathetic nerves to regulate gastrointestinal movement.^[^ ^25^ ^]^

The intestinal microbiota and the CNS are bidirectional and multichannel.^[^ ^26^ ^]^ The abnormalities in intestinal microorganisms lead to the secretion of amyloid proteins and lipopolysaccharides (LPS), increase intestinal permeability, and enable other cytokines, such as the LPS amyloid protein, to enter the intestinal wall and stimulate Toll‐like receptor 4 (TLR4) and other TLRs to produce inflammatory cytokines.^[^ ^27^ ^]^ These cytokines increase the permeability of the blood–brain barrier (BBB) and can enter the brain tissue, inducing neuroinflammation and nerve injury, and depositing amyloid plaques. Probiotics can strengthen the epithelial connection, protect the mucosal barrier, and reduce intestinal permeability, and the damage to the nervous system caused by intestinal inflammatory factors entering the blood circulation.^[^ ^28^ ^]^

An increasing body of evidence indicates that chemical signaling between enteric neurons and the gut microbiota influences their functions. The activation of aryl hydrocarbon receptors in adult mice has been shown to affect gut motility via the ENS.^[^ ^29^ ^]^ Microbiota products, such as short‐chain fatty acids (SCFAs),^[^ ^30^ ^]^ bacterial cell wall components, and others, have been found to affect rodent gut motility and ENS activity.^[^ ^31^ ^]^ A recent study reported that the microbial product 5‐hydroxytryptamine (5‐HT) influences the ENS through the microbial–gut–brain axis.^[^ ^32^ ^]^ Microbial products in the brain also affect neuronal pathways. In addition, ENS abnormalities have been linked to neuropathic chronic intestinal pseudo‐obstruction,^[^ ^33^ ^]^ and primary disorders of the CNS, including autism spectrum disorder, AD, and PD.^[^ ^34^ ^]^ The ENS, sometimes referred to as the “second brain,” is a network of neurons lining the gastrointestinal tract that can operate independently and communicate with the CNS via the VN.

#### 2.1.3 Nervous System Regulation of the Brain and Bone Health

Recent studies have shown that the nervous system regulates skeletal metabolism, and skeletal interception regulates bone homeostasis.^[^ ^35^ ^]^ In addition to the central regulators and brain‐derived molecules that influence bone with transmitters of the sympathetic, parasympathetic, and sensory nervous systems, bone‐derived mediators are released from bone cells and bone marrow and can impact brain function. This reciprocal interaction suggests that bone may serve as a pivotal “afferent” regulator of cerebral development, function, and pathophysiology.^[^ ^9^ ^]^ Peripheral nerves actively contribute to bone development and repair by transmitting various signals, including through neurotransmitters, neuropeptides, axon guidance factors, and neurotrophins. Concurrently, the bone provides a microenvironmental niche for these nerves, offering mechanical support and a protective enclosure within its internal milieu.^[^ ^36^ ^]^

Bone metabolism is directly controlled by neural pathways mediated by the CNS, and sensory and autonomic nerve fibers are involved in the regulation of bone tissue.^[^ ^11^ ^]^ There is a balance between the CNS‐mediated neuropeptide network, which plays a central role in balancing osteoblasts (OB) and osteoclasts (OC), as well as bone formation and resorption.^[^ ^9^ ^]^ Hence, skeletal metabolism is strictly regulated by the nervous system, whereas sensory regulation within the skeleton maintains homeostasis.

The ENS also plays a significant role in the regulation of bone metabolism and the maintenance of bone health. The ENS communicates with bone cells through various signaling molecules, including neuropeptides and neurotransmitters. For example, the neuropeptide substance P, which is produced by ENS neurons, stimulates bone formation by increasing the activity of OB.^[^ ^37^ ^]^ In contrast, other ENS signaling molecules, such as vasoactive intestinal peptides, have been shown to inhibit bone resorption by decreasing the activity of OC, which break down bone tissue.^[^ ^38^ ^]^ Moreover, the ENS regulates the gut microbiota, which in turn affects bone health.^[^ ^39^ ^]^ Overall, the ENS plays a crucial role in regulating bone metabolism and maintaining bone health.

**Figure 2:**
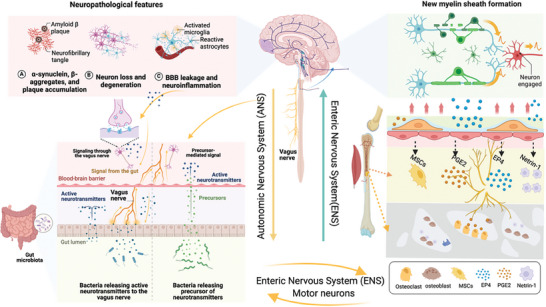
Neural pathways regulation in the brain–gut–bone axis. Communication between the brain and gut occurs through the VN and ANS in the spinal cord, which influences each other through complex neurohumoral pathways. The VN is a major neuronal pathway that runs from the brainstem to innervate the gut and ENS. The VN and ENS afferent pathways can be stimulated by neurotransmitters from the CNS, leading to changes in the gut microbiota and environment. The ENS receives signals from the central sympathetic and parasympathetic nerves, which regulate gastrointestinal movement and communicate with bone cells through neuropeptides and neurotransmitters. Dysfunction in the ENS can lead to changes in the gut microbiota, inflammation, and altered bone remodeling. OB secrete prostaglandin E2 (PGE2), which activates the PGE2 receptor 4 (EP4) in sensory nerves, serving as the ascending skeletal interoceptive pathway involved in bone remodeling. Sensory nerves that innervate the gut connect with skeletal nerves, allowing communication between the ENS and bone cells. These sensory neurons send signals to the spinal cord, where they are integrated with other sensory information and relayed back to the gut via motor neurons. CNS‐mediated neuropeptide networks play a crucial role in balancing osteoblast and osteoclast activity for proper bone formation and absorption. Figures were created with BioRender.com.

Skeletal nerves, which are part of the somatic nervous system, innervate the skeletal muscles and bones.^[^ ^40^ ^]^ However, they can also indirectly affect the gut through their connection to the ANS. Skeletal nerves can influence the ANS by activating the sympathetic nervous system and parasympathetic nervous system.^[^ ^41^ ^]^ In addition, the skeletal nerves can also affect the gut through their connection with the ENS. Skeletal nerves interact with the ENS through their connection with the sensory neurons that innervate the gut.^[^ ^42^ ^]^ These sensory neurons send signals to the spinal cord, where they can be integrated with other sensory information and relayed back to the gut via motor neurons.^[^ ^42^ ^]^ Overall, skeletal nerves indirectly influence many functions of the gut through their connection with the ANS and ENS. **Figure** **2** provides a detailed illustration of neural pathway regulation of the brain–gut–bone axis.

In conclusion, dysregulation of the brain–gut–bone axis may play a significant role in the development of neurodegenerative diseases. Intricate interactions between the nervous systems, including the VN and ANS, and the gut involves communication with the gut microbiota, the immune system, inflammatory responses, and skeletal nerves. This communication imbalance can lead to increased inflammation, gut permeability, and neuronal damage, accelerating the progression of neurodegenerative diseases.

### 2.2 Immune Regulation

#### 2.2.1 Crosstalk between the Gut Immune System and the Neuroimmune System

The development of the gut immune system depends on the gut microbiota.^[^ ^43^ ^]^ The gut microbiota communicates with systemic immune cells, regulates the gut epithelial barrier, and permeates the gut‐associated lymphoid tissue and the bloodstream.^[^ ^44^ ^]^ Certain bacteria stimulates effector T cell differentiation, and then promotes T cell brain infiltration, as well as, secrete inflammatory cytokines, disrupting the integrity of the BBB, leading to neuroimmune inflammatory responses and disease development.^[^ ^45^ ^]^

The gut is home to a vast number of immune cells, including T cells, B cells, and various types of innate immune cells such as macrophages, dendritic cells, and innate lymphoid cells that interact with the gut microbiota through various mechanisms, such as direct contact, secretion of immune modulators, and recognition of microbial antigens.^[^ ^46^ ^]^ Metabolites of the intestinal microbiota can trigger an immune response, inducing intestinal inflammation and PD development.^[^ ^47^ ^]^

The gut microbiota also plays a key role in maintaining the integrity of the gut epithelial barrier, which is critical for preventing the entry of harmful microorganisms and toxins into the bloodstream.^[^ ^48^ ^]^ Dysbiosis, or an imbalance in the gut microbiota leads to increased gut permeability and inflammation, contributing to the development of various diseases.^[^ ^49^ ^]^ Inflammatory factors increase the permeability of the BBB, allowing their entry into the brain tissue and causing neuroinflammation, nerve damage, and the deposition of amyloid proteins, leading to neuronal death and directly affecting brain function.^[^ ^50^ ^]^

The stress response to CNS injury alters gut microbiota and stimulates inflammatory immune cells, which migrate to the CNS and exacerbate neuroinflammation.^[^ ^51^ ^]^ In addition to immune cell activation, stress‐induced dysbiosis can lead to the migration of immune cells from the gut into the brain. This migration can occur through a variety of mechanisms, including disruption of the BBB and activation of immune cells in the gut, which then migrate to the brain.^[^ ^52^ ^]^ Once in the brain, the immune cells contribute to the development of neuroinflammation, which has been implicated in various neurological disorders.^[^ ^53^ ^]^

Based on this, we believe that the development of the gut immune system relies on the gut microbiota. Dysbiosis of the gut microbiota leads to increased gut permeability and exacerbated inflammation, thereby contributing to the development of various diseases. The stress response to CNS injury alters the gut microbiota and stimulates the migration of inflammatory immune cells to the CNS, exacerbating neuroinflammation.

#### 2.2.2 Gut Microbiota Regulates Bone Metabolism by Regulating the Host Immune System

Gut microbiota influences both the immune system and bone metabolism; gut microbial dysbiosis has been associated with both neurodegenerative and bone‐related diseases.^[^ ^54^ ^]^ The presence of colonizing bacteria in the intestine leads to the preferential accumulation of Tregs in the lamina propria of the colon, thus affecting the number and function of Tregs in the intestine and finally modulating bone metabolism.^[^ ^11^ ^]^ For example, *Bacillus clausii* promotes bone formation by increasing Treg cells in an ovariectomy (OVX) mouse model,^[^ ^55^ ^]^ and *Lactobacillus rhamnosus GG* treatment regulates bone anabolism via Treg cell‐mediated regulation of CD8^+^ T cell Wnt10b production.^[^ ^56^ ^]^

Emerging evidence suggests intestinal filamentous bacteria increase IFN‐γ production and IL‐17, which play an essential role in bone formation in vivo and rescue OP in mice following OVX.^[^ ^57^ ^]^ For example, the probiotic bacterium *Lactobacillus acidophilus* inhibits bone loss in OVX mice by modulating the Treg‐Th17 cell balance.^[^ ^58^ ^]^ Moreover, the segular filamentous bacteria, a type of gut microbe, regulates the homeostasis of OB and OC by producing IL‐17 and IFN‐γ.^[^ ^59^ ^]^ Mature helper T17 (Th17) cells and the related inflammatory factor IL‐17 are the main driving forces in the pathogenesis of bone loss diseases such as rheumatoid arthritis and OP. IL‐17 significantly upregulates the expression of RANKL and its receptor RANK and enhances the activity of OC, thus disrupting bone metabolic balance and aggravating bone destruction.^[^ ^60^ ^]^ Thus, an imbalance in the gut microbiology can inhibit the differentiation of Tregs, leading to Th17 cell differentiation and promoting OC differentiation.^[^ ^11^, ^61^ ^]^

In addition to affecting Tregs and T cells, the gut microbiota also functions in B cell development by producing osteoprotegerin (OPG), an OC inhibitor for effective bone resorption.^[^ ^62^ ^]^ B cells are a key source of OPG and are potent regulators of bone turnover.^[^ ^63^ ^]^ Inflammatory processes exert a substantial impact on bone remodeling, leading to bone loss through the heightened stimulation of OC activity. B cells transplantation into mice lacking B cells normalized OPG production and prevented bone resorption and loss of bone mass.^[^ ^64^ ^]^ OPG regulates autophagy‐related genes and AMP‐activated protein kinase/mTOR/p70S6K signaling, which ultimately inhibits OC differentiation and bone resorption.^[^ ^65^ ^]^ The gut microbiota produces SCFAs as metabolites that have anti‐inflammatory properties and inhibit the activation of nuclear factor kappa‐light‐chain‐enhancer of activated B cells. This helps reduce autoimmune inflammation and supports osteoimmunity.^[^ ^66^ ^]^ The immune system may be involved in OP pathogenesis mediated by intestinal microorganisms. Secondary OP is a common consequence of immune system abnormalities. Collectively, these studies suggest that the gut microbiota regulates bone metabolism by altering the host's immune status.

Studies have suggested that the gut microbiota plays a significant role in the regulation of bone metabolism. The gut microbiota closely interacts with the host immune system, and this interaction has implications for bone health. The gut microbiota and the immune system have a mutual relationship; the immune system helps maintain a balanced gut microbiota composition, and in turn, the gut microbiota influences immune function. This bidirectional communication between the gut microbiota and the immune system can affect bone health.

#### 2.2.3 Neuroinflammation in the Brain Affects Bone Metabolism and Bone Absorption

Inflammation is a common feature of both neurodegenerative and bone‐related diseases, and contributes to bone loss and impaired bone formation by promoting the activation and differentiation of OC (cells that break down bone tissue) and inhibiting OB (cells that build new bone tissue).^[^ ^67^ ^]^ During the process of neurodegeneration, neuronal death and neuroinflammatory responses produce a series of inflammatory mediators such as cytokines and chemokines, which affect bone metabolism and absorption, leading to the occurrence of bone‐related diseases such as OP and fractures.^[^ ^68^ ^]^ In addition, proinflammatory cytokines, such as tumor necrosis factor‐alpha (TNF‐α) and interleukin‐6 (IL‐6), stimulate the production of receptor activator of nuclear factor kappa‐B (NF‐κB) ligand, a protein required for OC differentiation and activation.^[^ ^69^ ^]^ This increases bone resorption and decreases bone density.

Immunological cells and molecules are involved in neurodegenerative and bone‐related diseases. For example, T cells and B cells, which are involved in the immune response, can produce RANKL and OPG, a protein that inhibits RANKL, thereby preventing OC activation. These molecules affect the differentiation and function of the bone cells, thereby affecting their metabolism and absorption.^[^ ^70^ ^]^

Recent studies indicate the presence of a hidden “alarm system” within the skull that can effectively monitor neuroinflammation with unique subtypes of neutrophils. Research has revealed differential expression of neutrophil‐associated pathways and unique synaptic protein features in the skull. The skull bone marrow not only has a distinct cellular composition and molecular profiles but can also reflect neuroinflammation in brain diseases. Consequently, noninvasive cranial imaging holds great promise for monitoring brain health and diagnosing neurological conditions. Moreover, specific immune cell types may also exist in the skeletal systems of other parts of the body. These cells could potentially be employed for the surveillance of CNS diseases, such as AD, facilitating early detection and diagnosis to prevent disease onset. This exciting development opens new avenues for the diagnosis and treatment of neurodegenerative disorders.^[^ ^71^ ^]^ Therefore, maintaining healthy and diverse gut microbiota may be important for preserving optimal immune function and bone health.

**Figure 3:**
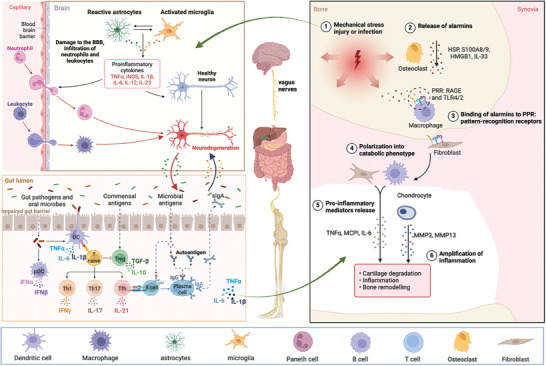
Immune regulation of brain–gut–bone axis. Neurodegenerative diseases are characterized by inflammation and immune system abnormalities that result in neuronal death and neuroinflammatory responses. This triggers the production of inflammatory mediators such as cytokines and chemokines. The inflammatory factors cause an imbalance in gut flora, which is home to a plethora of immune cells such as T cells, B cells, macrophages, dendritic cells, and innate lymphoid cells. Dysbiosis of the gut microbiota increases gut permeability and inflammation. Moreover, gut bacteria stimulate the differentiation of effector T cells, which migrates to the brain and disrupt the integrity of the BBB, leading to neuroinflammation and the development of neurological diseases. Gut microbes play a crucial role in regulating host health, particularly bone development. Dysbiosis of the gut inhibits the differentiation of Tregs and promote Th17 cell differentiation, leading to OC differentiation and bone destruction. Inflammation is a common feature of both neurodegenerative and bone‐related diseases, which exacerbates bone loss and impair bone formation by promoting OC activation and inhibiting OB. T cells and B cells can produce RANKL and OPG, a protein that inhibits RANKL and prevents osteoclast activation. Dysregulation of T and B cells in neurodegenerative diseases can, therefore, affect bone metabolism and contribute to bone loss. Figures were created with BioRender.com.

The immune system plays a crucial role in neurodegenerative diseases and the brain–gut–bone axis is instrumental in regulating the impact of the immune system on these diseases. Gut microbiota, immune cells, and inflammatory responses play significant roles in this axis, and their imbalance can contribute to the development and progression of diseases. The detailed mechanism of immune regulation via the brain–gut–bone axis is shown in **Figure** **3**. In summary, investigating the brain–gut–bone axis is of great significance for gaining a deeper understanding of the pathogenesis of neurodegenerative diseases and for developing potential therapeutic approaches.

### 2.3 Endocrine Regulation

#### 2.3.1 HPA Axis Regulation

The HPA axis is the main endocrine system of the body and is widely involved in the stress response.^[^ ^72^ ^]^ In response to stress, the hypothalamus releases a corticotropin‐releasing hormone (CRH), which stimulates the pituitary gland to release adrenocorticotropic hormones (ACTH) and Neuropeptide Y (NPY). ACTH stimulates the adrenal glands to release cortisol, which helps the body cope with stress by increasing blood sugar levels, suppressing the immune system, and altering metabolism.^[^ ^73^ ^]^

Dysregulation of the HPA axis can lead to increased stress and anxiety and has been implicated in the pathophysiology of neurodegenerative diseases.^[^ ^74^ ^]^ Cortisol levels are elevated in the cerebrospinal fluid of patients with AD, suggesting dysregulation of the HPA axis. This dysregulation contributes to the cognitive decline and neuronal damage observed in the disease.^[^ ^74b^ ^]^ Similarly, patients with PD show signs of HPA axis dysfunction, as indicated by disrupted cortisol rhythms and abnormal stress responses.^[^ ^75^ ^]^

Gut microbiology is closely related to the HPA axis. Huo et al. found that germ‐free mice exhibited stronger HPA axis activity under mild stress. However, the HPA axis returned to normal after proper supplementation with colonic bacteria.^[^ ^76^ ^]^ The gut barrier integrity is affected by the regulatory effects of cortisol on neuroimmune signaling.^[^ ^14^ ^]^ Activation of the HPA axis leads to the releases of cortisol to increase intestinal permeability and regulate endocrine cells, immune cells, cytokines, and other factors that affect the gastrointestinal microenvironment, thereby affecting the composition of intestinal bacteria.^[^ ^77^ ^]^

The HPA axis regulates bone metabolism and plays a crucial role in modulating bone density and strength. Dysregulation of the HPA axis leads to increased bone resorption and decreased bone density, which contribute to bone‐related diseases, such as OP.^[^ ^78^ ^]^ Elevated serotonin levels are also associated with bone loss.^[^ ^79^ ^]^ Overall, the HPA axis plays a critical role in the regulation of various physiological processes in the brain, gut, and bones, and its dysregulation leads to various disorders and diseases.

#### 2.3.2 Function of Neurotransmitters Derived from Gut Microbiota and Brain

The synthesis of neurotransmitters mediated by the gut microbiota has significant implications for cognition.^[^ ^80^ ^]^ The gut microbiota can produce precursor molecules for neurotransmitters and facilitate the synthesis of neurotransmitters through dietary metabolism, or a combination of both. Certain neurotransmitters precursors can traverse the BBB and actively contribute to the synthesis of neurotransmitters within the brain.^[^ ^81^ ^]^

Several hormones synthesized by the gut microbiota also serve as neurotransmitters, which are more significant than the influence of the intestinal microbiota in the CNS.^[^ ^82^ ^]^ Certain bacterial taxa can stimulate neurotransmitter synthesis, which is released by enteroendocrine cells.^[^ ^83^ ^]^ For instance, *Lactobacillus bacteria* produce gamma‐aminobutyric acid (GABA) and serotonin, which are crucial inhibitory neurotransmitters of the brain.^[^ ^81^ ^]^ Neurotransmitters like glutamate, GABA, dopamine, and serotonin, are synthesized in the brain from local pools of amino acid precursors derived from the diet, which are transported across the BBB and converted into functional neurotransmitters with the help of host enzymes.^[^ ^84^ ^]^ The intestinal microbiome can influence host behavior by regulating neurotransmitter precursors.^[^ ^81^ ^]^

Brain‐derived neurotrophic factor (BDNF), a critical neurotrophic factor that regulates neuronal regeneration, plays a crucial role in promoting learning, memory, and neurodegenerative diseases.^[^ ^85^ ^]^ The regenerative capacity of hippocampal neurons in patients with AD progressively declines with disease progression, which is characterized by the downregulation of genes involved in regulating plasticity, decreased dendritic density, impaired synaptic plasticity, and cognitive dysfunction.^[^ ^86^ ^]^ Therefore, enhancing the regenerative capacity of hippocampal neurons is important for improving cognition. BDNF is the most abundant neurotrophin in the brain and is a myokine. Exercise has been shown to increase BDNF mRNA levels in the muscles of healthy rodents and humans.^[^ ^87^ ^]^ Additionally, recent evidence suggests that BDNF produced by muscles may act as an endocrine signal for vascular cells and perivascular adipose depots.^[^ ^88^ ^]^ Hence, increasing the BDNF in the brain to promote hippocampal neuronal regeneration is an important approach to improving cognition.

Neurotransmitters derived from the gut microbiota signal to the brain via endocrine pathways, affecting neuronal activity and neurotransmitter balance in the brain. The brain can also influence gut microbiota composition and function through the autonomic nervous system and stress response. Alterations in the gut microbiota composition and neurotransmitter production may contribute to the development and progression of neurodegenerative diseases.

#### 2.3.3 Bone Homeostasis Modulated by Gut Microbiota and the Endocrine System

There exists a complex interplay between the gut microbiota and endocrine system that affects bone homeostasis. For example, hormones produced by the endocrine system such as leptin and adiponectin influence the composition of the gut microbiota, leading to changes in bone mass and density.^[^ ^89^ ^]^ The lack of these hormones contributes to intestinal permeability and osteoclastic bone resorption in a TNF‐ and RANKL‐ dependent manner.^[^ ^90^ ^]^ The gut microbiota also affects the production and secretion of hormones, such as insulin and glucagon, which can regulate bone metabolism.^[^ ^91^ ^]^ Yan et al. demonstrated that gut microbial colonization significantly increased serum insulin‐like growth hormone 1 (IGF‐1) levels and promoted bone growth and formation in germ‐free mice.^[^ ^92^ ^]^ In addition, increasing 5‐HT levels prevented OP caused by estrogen deficiency, suggesting that estrogen works synergistically with 5‐HT to regulate bone metabolism.^[^ ^78^ ^]^ These hormones regulate intestinal microbiota through different mechanisms, thereby affecting the secretion and metabolism of bone cells. Further human studies are essential to confirm whether the intestinal microbiota influence bone metabolism by modulating hormone production.

**Figure 4:**
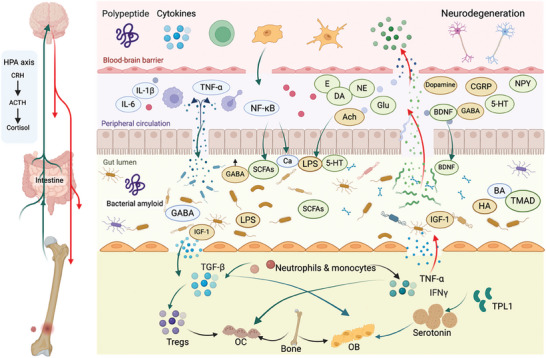
Endocrine regulation of the brain–gut–bone axis. Dysregulation of the HPA axis is implicated in contributing to the cognitive decline and neuronal damage observed in the neurodegenerative diseases. The brain regulates the composition of intestinal bacteria by activating the HPA axis to release cortisol, increasing intestinal permeability, and influencing the gastrointestinal microenvironment through the regulation of endocrine cells, immune cells, cytokines, and other factors. The neurotransmitters such as glutamate, GABA, dopamine, NPY, BDNF, and serotonin are synthesized in the brain from neurotransmitter precursors and affect the balance of intestinal microbiota. Several hormones synthesized by gut microbiota such as GABA and serotonin are crucial inhibitory neurotransmitters in the brain. Gut flora regulates the production of human hormones such as growth hormone, insulin‐like growth factor, and gonadal hormone, thereby affecting bone homeostasis. Cortisol can stimulate the production of receptor activator of NF‐κB ligand, a protein required for OC differentiation and activation. Elevated levels of serotonin have been linked to bone loss. Estrogen deficiency may enhance intestinal barrier permeability, promote CD4^+^ T cell activation, increase the levels of circulating LPS. And increasing the level of 5‐HT can prevent OP caused by estrogen deficiency. Some hormones such as leptin and adiponectin, can influence the composition of the gut microbiota, leading to changes in bone mass and density. The lack of these hormones contributes to intestinal permeability and OC bone resorption in a way that is dependent on TNF and RANKL. Unbalanced microbiota can lead to changes in LPS levels of inflammatory bacteria directly into the brain or induce elevated systemic immune responses. In addition, the gut microbiota can also affect the production and secretion of insulin and glucagon, which can regulate bone metabolism. Excessive glucocorticoids can inhibit the synthesis of bone matrix proteins and the differentiation of OB and its precursors. Figures were created with BioRender.com.

In addition, excessive glucocorticoids levels inhibit the synthesis of bone matrix proteins and the differentiation of OB and their precursors, reduce the synthesis of osteocollagen, and decrease the synthesis and secretion of osteocalcin, suppressing bone formation.^[^ ^93^ ^]^ More importantly, estrogen deficiency may enhance intestinal barrier permeability, promote CD4^+^ T cell activation, increase circulating LPS levels, and the production of proinflammatory factors.^[^ ^94^ ^]^ Overall, the gut microbiota can affect bone health by producing hormones that regulate bone formation and resorption. A schematic diagram of the endocrine regulation of the brain–gut–bone axis was shown in **Figure** **4**.

The gut microbiota can influence the synthesis and metabolism of neurotransmitters, such as serotonin, dopamine, and GABA, which are important signaling molecules in the nervous system. Changes in these neurotransmitters may be associated with pathological processes in neurodegenerative diseases. Overall, the brain–gut–bone axis is closely linked to the development and progression of neurodegenerative diseases through the regulation of the endocrine system and its impact on neurotransmitters. Studying the mechanisms of the brain–gut–bone axis will help us better understand the complexity of these diseases and provides a theoretical basis for the development of new therapeutic strategies. However, further research is needed to uncover the specific role and potential therapeutic approaches involving the brain–gut–bone axis in neurodegenerative diseases.

### 2.4 Metabolism System

#### 2.4.1 The Diverse Roles of Gut Microbiota Metabolism in the Brain

The gut microbiota affects the physiological, behavioral, and cognitive functions of the brain. The gut microbiota produce abundant metabolites in the intestine that can affect both intestinal and nonintestinal tissues.^[^ ^95^ ^]^ Common examples of these metabolites include dopamine, 5‐HT, glutamate, BDNF, NPY, γ‐aminobutyric acid, SCFAs, tryptophan (Trp), and neurotoxic metabolites such as ammonia, phenols, amines, phenolic acids, and d‐lactic acid.^[^ ^96^ ^]^

Gut microbiota metabolites regulate brain metabolism and function. For example, SCFAs interact with G protein‐coupled receptors (GPCRs) or histone deacetylases (HDACs) and act on the brain via body fluids, immune pathways, and other routes.^[^ ^97^ ^]^ SCFA stimulates the synthesis and secretion of 5‐HT in the gut, which binds to 5‐HT receptors and regulates BBB permeability, intervenes in neuronal development and differentiation, modulates emotions through neural signals, and regulates blood circulation to affect brain function.^[^ ^98^ ^]^ In addition, 5‐HT is an inhibitory neurotransmitter secreted by enterochromaffin cells in the gut, which plays an important role in neuronal development and synapse formation.^[^ ^99^ ^]^ 5‐HT is an important gastrointestinal signaling molecule that transmits signals from the gut to intrinsic or extrinsic neurons, thereby affecting intestinal motility, secretion reflexes, nutrient absorption, and other functions.^[^ ^82^ ^]^

Trp is an essential amino acid associated with the synthesis of key compounds such as 5‐HT and kynurenine (KYN), while aromatic hydrocarbon (AhR) receptors are critical for Trp metabolites in brain signaling.^[^ ^100^ ^]^ AhRs activated by Trp metabolites act as chemical messengers that mediate the intestinal and the bidirectional relationship between microbes and the CNS and can regulate host homeostasis in vivo through immune, metabolic, and vagal communication pathways.^[^ ^101^ ^]^ Therefore, these microbial metabolites play an important role in regulating the gut–brain axis.

Metabolites derived from gut bacteria have been shown to play a substantial role in mitigating the onset of life‐threatening brain disorders.^[^ ^102^ ^]^ Gut microbiota metabolites guide the development of immune cells and neuroinflammatory responses in the brain.^[^ ^103^ ^]^ Microglia are a group of immune cells in the CNS responsible for the maintenance of brain homeostasis and responses to injury or stress. Butyrate, an SCFA produced by gut bacteria, regulates the genes involved in the maturation of microglia, alters their morphology, promotes the development of anti‐inflammatory microglia, and reduces neuroinflammation in animal models of CNS injury.^[^ ^104^ ^]^ Metagenomics has been utilized to identify and characterize gut microbiota and microbiota‐derived metabolites. Additionally, these microbiota‐derived metabolites have been shown to play a significant role in neurodegenerative diseases such as AD, PD, and other neurological disorders, highlighting the importance of the microbiota–gut–brain axis in these conditions.^[^ ^105^ ^]^ The regulation of host physiology and behavior by the gut microbiota plays an important role, and the metabolites produced by the gut microbiota are important pathways in this process.

#### 2.4.2 The Effect of Gut Microbiota Metabolism on Bone Health

The interaction between the gut microbiota and bone, known as the gut microbiota–bone axis, plays a significant role in maintaining skeletal homeostasis and bone mineral density. This axis involves the production of diverse metabolites by the gut microbes, including SCFAs, Trp metabolites, and exogenous and endogenous polyamines, which have profound effects on bone health.^[^ ^106^ ^]^ A study of 426824 participants from the UK biobank cohort demonstrated a link between the growth of gut bacteria and bone mineral density using genome‐wide association study (GWAS) summary statistics and provided evidence of a causal relationship between microbiota and bone development.^[^ ^107^ ^]^ Senile OP is associated with changes in the microbiota, leading to a reduction in bone mineral density (BMD). The fecal microbiota profiles of 106 postmenopausal individuals (33 with osteopenia, 42 with osteoporosis, and 31 with normal BMD) showed that bacterial overgrowth in the gut could contribute to the development of OP.^[^ ^108^ ^]^

SCFAs have become a hotspot in gut–bone axis research over the past few decades. SCFAs, generated from the bacterial fermentation of complex carbohydrates, represent an important energy source for intestinal epithelial cells and facilitate intestinal barrier functions.^[^ ^109^ ^]^ Additionally, SCFAs directly affect the formation of OC and OB in addition to their regulatory roles in the immune system. SCFAs inhibit the differentiation of bone marrow cells into OC by inhibiting the activity of HDACs. The inhibition of HDACs is one of the mechanisms by which SCFAs suppress the differentiation of bone marrow cells into OC.^[^ ^110^ ^]^ SCFAs bind to G‐protein‐coupled receptors, and it has been shown that the free fatty acid G‐protein‐coupled receptors (GPR41 and GPR43) are responsible for suppressing OC.^[^ ^111^ ^]^ Moreover, regulation of the OPG and Wnt signaling pathways by SCFAs is crucial for bone formation and mineralization. A previous study demonstrated that administration of SCFAs to antibiotic‐treated mice enhanced insulin‐like growth factor 1 (IGF‐1) levels, a hormone that affects skeletal growth.^[^ ^92^ ^]^ The gut microbiota plays a significant role in bone remodeling by modulating SCFA production.^[^ ^112^ ^]^

Trp metabolites, particularly kynurenic (KYN), and serotonin, are closely associated with bone metabolism. Compounds produced in the KYN pathway, including kynurenic acid (KYNA), 3‐hydroxykynurenine (3‐HKYN), and anthranilic acid,^[^ ^16^ ^]^ have key effects on the promotion of bone‐aging phenotypes. KYNA has been shown to inhibit the differentiation of OB and RANKL‐induced OC genesis, and 3‐HKYN has been shown to reduce the viability of osteoblast‐like cells owing to its pro‐oxidative nature.^[^ ^113^ ^]^ KYN itself influences the proliferation of bone marrow mesenchymal stem cells into the osteoblastic cell lineage,^[^ ^114^ ^]^ elevated peripheral KYN levels lead to the destruction of bone structure via the aryl hydrocarbon receptor pathway.^[^ ^115^ ^]^

Evidence suggests that gut microbes produce both exogenous and endogenous polyamines. Exposure to warm conditions has been shown to alter the gut microbiota and increase gut polyamine biosynthesis, including acetylated spermidine and putrescine, which improves bone microarchitecture and strength in mice, protecting them against OVX‐induced bone loss.^[^ ^116^ ^]^ However, a cross‐sectional study revealed that elevated polyamine N‐acetyl‐putrescine levels were was negatively associated with spine BMD, demonstrating the double‐edged effect of polyamines, given that excess polyamine metabolism may impair osteoblast genesis.^[^ ^94^ ^]^

**Figure 5:**
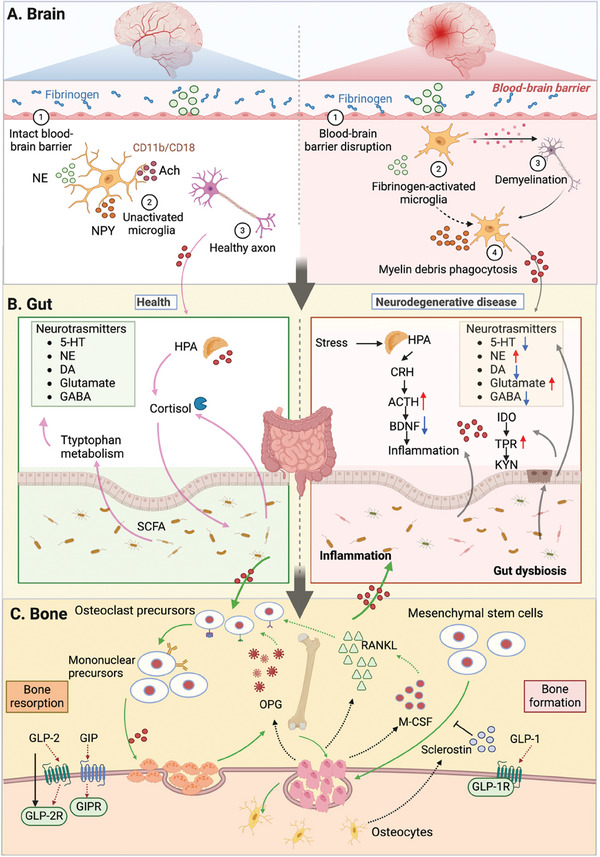
Metabolism in the brain–gut–bone axis. The response to CNS injury causes changes in gut microbiota and trigger the activation of inflammatory immune cells that migrate to the CNS and worsen neuroinflammation. The development of microglia can be influenced by metabolites produced by gut microbiota. One of the most significant impacts of gut flora is on the metabolism and absorption of neurotransmitters such as dopamine, 5‐HT, glutamate, BDNF, γ‐aminobutyric acid, SCFA, Trp, as well as neurotoxic metabolites such as ammonia, phenols, amines, phenolic acids, and d‐lactic acid. For instance, 5‐HT, an inhibitory neurotransmitter secreted by enterochromaffin cells in the gut, plays a crucial role in neuronal development and synapse formation. SCFA can stimulate the production and release of 5‐HT in the gut and also affect the formation of OC and OB, in addition to regulating the immune system. Studies have shown that SCFAs can suppress the differentiation of bone marrow cells into OC by inhibiting the activity of HDACs. Furthermore, experimental evidence indicates that gut microbiota has a significant impact on bone remodeling through the modulation of SCFA production. KYN and serotonin are closely related to bone metabolism. Microorganisms are capable of influencing the effects of microbial‐derived molecules on the skeleton. Figures were created with BioRender.com.

The gut microbiome plays a pivotal role in regulating bone health and influences postnatal skeletal development and involution. Disruptions in the microbiota composition and host responses to the microbiota contribute to pathological bone loss. Nutritional supplements containing prebiotics and probiotics have the potential to modulate microbiota composition, thereby preventing or reversing bone loss.^[^ ^117^ ^]^ Collectively, these observations will lead to a better understanding of the relationship between bone homeostasis and microbiota, thus providing new insights into the microbiota‐mediated bone development mechanisms. A diagram of the metabolic process along the brain–gut–bone axis is shown in **Figure** **5**.

#### 2.4.3 The Impact of Gut Microbiota‐Derived Vitamins on the Brain and Bones

The gut is a major source of nutrients, such as vitamins B and K, which are products of intestinal microorganisms, and these vitamins are known to play an important role in the development and function of the brain and bones.^[^ ^39^ ^]^ The metabolites produced by the gut microbiota have a significant impact on the nervous system of the host. Vitamin K2 prevents neuronal cell apoptosis, oxidative stress, and microglial activation, making it a promising therapeutic agent for AD.^[^ ^118^ ^]^ Vitamin K also slows down α‐synuclein fibrillation, leading to the formation of shorter fibrils and amorphous aggregates that are less prone to vesicle leakage, thus inhibiting the onset of PD.^[^ ^119^ ^]^ Studies have suggested that increasing dietary vitamin K intake in the diet can alleviate memory decline in older adults.^[^ ^120^ ^]^ Vitamins B9, B6, B12, and others have been found to alleviate brain atrophy in regions associated with cognitive decline in AD.^[^ ^121^ ^]^ Vitamin K supplementation has also been recommended for the treatment of patients with multiple sclerosis (MS) to reduce disease intensity and slow disease progression.^[^ ^118^ ^]^ Therefore, studying the relationship between vitamins and neurodegenerative diseases would be helpful for the prevention and treatment of these conditions, particularly by providing strong evidence for the use of vitamin K supplements in AD, PD, and MS.

Inadequate vitamin K levels are linked to bone and mineral abnormalities, specifically osteoporosis, bone fractures, and vascular calcification.^[^ ^122^ ^]^ Vitamin K is an essential component of osteocalcin and is abundant in the bone matrix.^[^ ^123^ ^]^ Compared with dietary intake, the production of vitamin K in the gut is an important contributor to vitamin supply. A decrease in the production of vitamin K by the gut microbiota can lead to an increase in the circulation of undercarboxylated osteocalcin.^[^ ^123^ ^]^ A lack of carboxylated osteocalcin in the bone matrix weakens the bone tissue and makes it more susceptible to fractures.^[^ ^89^ ^]^ In addition to potential malabsorption issues, bacteria overgrowth can affect the metabolism of essential nutrients, such as vitamin K and carbohydrates, potentially affecting the regulation of osteogenic processes.^[^ ^124^ ^]^ These metabolites, including SCFAs, Trp metabolites, and polyamines, contribute to brain and skeletal homeostasis and are influenced by the brain–gut microbiota–bone axis, which is also affected by essential nutrients such as vitamins B and K.

The brain–gut–bone axis influences the metabolism system in neurodegenerative diseases in several ways. First, the gut microbiota plays a crucial role in the digestion and absorption of nutrients. They participate in food breakdown and generate metabolites such as SCFAs and cholesterol metabolites. These metabolites can enter the brain through the bloodstream and affect neuronal function and metabolism. Second, the brain–gut–bone axis influences metabolic processes by regulating the endocrine system. Additionally, inflammatory reactions in the brain–gut–bone axis affect metabolic processes. An imbalance in the gut microbiota can lead to impaired intestinal barrier function, allowing bacteria and metabolites to enter the circulation and trigger an inflammatory response. Overall, the brain–gut–bone axis exerts significant effects on metabolism through gut microbiota, endocrine regulation, and inflammatory responses, which are closely associated with the development and progression of neurodegenerative diseases. In‐depth research into the metabolic mechanisms of the brain–gut–bone axis will help uncover the complexity of these diseases and provide a theoretical foundation for the development of new therapeutic strategies.

## 3 Brain–Gut–Bone Axis Imbalance: Pathology and Diseases

The roles of intestinal microbes and their host gut microecology in the maintenance of health and disease development cannot be ignored. Neurodegenerative diseases, which are associated with changes in bone mass and density, are also associated with an increased risk of OP and bone fractures.^[^ ^125^ ^]^ Collectively, dysregulation of the intestinal microecological environment and bone system are associated with AD,^[^ ^126^ ^]^ PD,^[^ ^127^ ^]^ HD,^[^ ^128^ ^]^ and MS,^[^ ^129^ ^]^ and other neurological dysfunctional diseases.

### 3.1 Alzheimer's Disease

AD is a CNS degenerative disorder characterized by memory loss, inability to perform daily activities, and behavioral disturbances and is the most common form of dementia in older adults.^[^ ^130^ ^]^ The pathological features of AD are mainly the formation of senile plaques composed of β‐amyloid protein (Aβ) deposits and the neurofibrillary tangles (NFTs) composed of hyperphosphorylated tau protein.^[^ ^131^ ^]^ Dysregulation of the Wnt/β‐catenin signaling pathway has been implicated in the development and progression of neurodegenerative diseases.^[^ ^132^ ^]^ Aberrant Wnt/β‐catenin signaling contributes to the accumulation of Aβ plaques and NFTs, which are hallmark pathological features of AD.^[^ ^133^ ^]^ Environmental and genetic factors are believed to contribute to the pathogenesis of AD, however, the fundamental cause of AD is remains unclear.

The gut microbiota is a key factor in the pathogenesis of AD. The gut microbiota can produce amyloid proteins, LPSs, and other immunogenic compounds that promote neuroinflammation and Aβ deposition in the brain, leading to the development of AD.^[^ ^134^ ^]^ Gut microbiota dysbiosis can cause intestinal epithelial cell damage, activate the TLR4/TNF‐α signaling, and induce neuroinflammation when TNF‐α and some proinflammatory factors cross the BBB.^[^ ^135^ ^]^ Additionally, the imbalance of the gut microbiota in patients with AD has been linked to increased intestinal permeability and inflammation, which can lead to the production of neurotoxic substances that contribute to disease progression.^[^ ^136^ ^]^

Recent studies have shown that the composition of the gut microbiota in patients with AD is altered, showing reduced bacterial diversity and an increased abundance of proinflammatory bacteria.^[^ ^137^ ^]^ A decrease in beneficial bacteria, such as *Bifidobacterium* and *Lactobacillus*, and an increase in harmful bacteria, such as *Escherichia coli* and *Helicobacter pylori* have been observed in patients with AD.^[^ ^138^ ^]^ Kim et al. found that transferring fecal microbiota from healthy mice to AD mice improved the deposition of Aβ plaques, tau protein pathology, glial reactions, and cognitive impairment in the brains of AD mice.^[^ ^139^ ^]^ Furthermore, the gut microbiota can influence the activity of intestinal macrophages and the expression of inflammatory monocyte‐related genes in the bloodstream. Inflammatory monocytes can directly affect the production of signaling molecules in the brain, thereby influencing the onset of AD.^[^ ^140^ ^]^ Thus, the regulation of the gut microbiota may provide new therapeutic targets for the treatment of AD.

Patients with AD are also considered to be at risk for OP. They contain excessive amyloid protein in the brain, which extends to peripheral organs and causes skeletal amyloid deposition, which will enhance the ligand signal of nuclear factor NF‐κB receptor activator and lead to enhanced osteoclast activity.^[^ ^141^ ^]^ Patients with AD have reduced hip bone density and a nearly twofold increased risk of hip fractures.^[^ ^142^ ^]^ Patients with OP may be deficient in vitamin D or have low levels of vitamin D‐binding protein, which prevents amyloid aggregation, thus linking vitamin D deficiency to AD and OP.^[^ ^141^, ^143^ ^]^ Postmenopausal women have a higher risk of AD and OP compared to the general population, suggesting that estrogen may affect brain aging and bone metabolism.^[^ ^141^, ^144^ ^]^ Hence, more clinical studies are needed to determine the role of estrogen in the risk of OP and AD in older adults.

The main neuropathological changes in AD are the extracellular deposition of Aβ as senile plaques and the intracellular accumulation of hyperphosphorylated tau protein as NFT,^[^ ^126^ ^]^ which lead to neuroinflammation, oxidative stress, mitochondrial dysfunction, enzyme system dysregulation, and neuronal death.^[^ ^145^ ^]^ Neuroinflammation is believed to play a critical role in the pathogenesis of AD.^[^ ^146^ ^]^ Neuroinflammation may lead to dysbiosis of the gut microbiota, resulting in changes in the gut microbiota of patients with AD.^[^ ^147^ ^]^ The gut microbiota also affects the production and secretion of hormones such as leptin and adiponectin, leading to changes in bone mass and density.^[^ ^89^ ^]^ The increased risk of fractures in patients with AD makes AD a risk factor for OP.^[^ ^148^ ^]^

**Figure 6:**
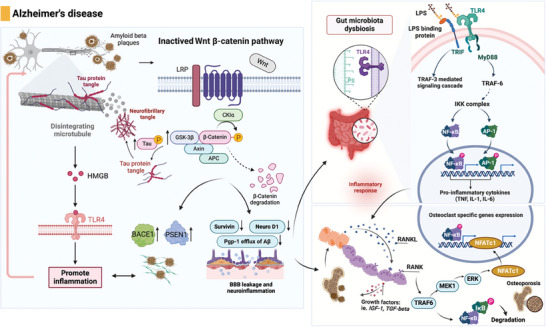
The regulatory mechanisms of AD through the brain–gut–bone axis. AD is characterized by the formation of senile plaques containing Aβ deposits and neurofibrillary tangles composed of hyperphosphorylated tau protein. The accumulation of beta‐amyloid plaques and neurofibrillary tangles, characteristic features of AD, is influenced by abnormal Wnt/β‐catenin signaling. Inactivation of Wnt/β‐catenin leads to decreased survivin levels and enhanced permeability of the BBB by reducing tight junction proteins. It also promotes Aβ production through the promotion of BACE1 and PSEN1 mutations, impairs the elimination of Aβ from the brain by reducing Pgp‐1 efflux, increases tau phosphorylation mediated by GSK‐3β, and triggers the formation of neurofibrillary tangles and neuroinflammation. Dysbiosis of gut microbiota can damage intestinal epithelial cells and activate the TLR4/TNF‐α signaling pathway. This activation induces neuroinflammation when TNF‐α and certain proinflammatory factors cross the BBB. In individuals with AD, excessive amyloid protein is present in the brain and can spread to peripheral organs, leading to amyloid deposition in skeletal tissues. This deposition enhances the ligand signal of the nuclear factor NF‐κB receptor activator, resulting in increased osteoclast activity. The binding of RANKL to RANK initiates a cascade involving TRAF‐6, which phosphorylates the inhibitor of NF‐κB kinase, leading to the degradation of IκB. This release of NF‐κB allows it to translocate to the nucleus and initiate the transcription of osteoclast‐specific genes. Additionally, the RANK signaling pathway activates ERK through MEK1, leading to the phosphorylation and activation of ERK, which is involved in osteoclast survival, motility, and cytoskeletal rearrangement. BACE 1: β‐site APP cleaving enzyme 1; PSEN1: presenilin 1; GSK‐3β: glycogen synthase kinase‐3β; TLR4: toll‐like receptor 4; TNF‐α: tumor necrosis factor‐α; TRAF‐6: tumor necrosis factor receptor‐associated factor‐6; NF‐κB: nuclear factor‐kappa B; MEK1: mitogen‐activated protein kinase‐1; ERK: extracellular signal‐regulated kinase; RANK: receptor activator NF‐κB; RANKL: receptor activator NF‐κB ligand; IκB: inhibitor of NF‐κB; NFATc1: nuclear factor of activated T‐cells‐cytoplasmic 1. Figures were created with BioRender.com.

Therefore, the brain–gut–bone axis exhibits complex interactions in AD and it has been shown in **Figure** **6**. Neuroinflammation, gut microbiota dysbiosis, and bone health are correlated. In‐depth research on the molecular mechanisms of the brain–gut–bone axis is important for understanding the pathological mechanisms of AD, developing new treatment approaches, and formulating preventive strategies. This may involve interventions and the management of AD by balancing the gut microbiota, controlling neuroinflammatory responses, and maintaining bone health. However, further research is needed to enhance our understanding of the brain–gut–bone axis in AD.

### 3.2 Parkinson's Disease

PD is the second most common progressive neurodegenerative disease and is clinically characterized by bradykinesia, muscle rigidity, resting tremors, and postural gait disorders.^[^ ^149^ ^]^ Its hallmark is the permanent loss of dopaminergic neurons in the substantia nigra and cholinergic neurons in the meynert basal nucleus in the CNS, as well as the continuous accumulation and aggregation of α‐synuclein (α‐Syn) in affected neurons, leading to the formation of Lewy bodies and Lewy neurites.^[^ ^149^, ^150^ ^]^ α‐Syn is transported across the BBB to the mouse brain. Changes in the composition of the gut microbiota promote the spread of α‐Syn aggregates from the ENS to the brain, leading to characteristic neurodegenerative changes.^[^ ^151^ ^]^ Altered Wnt/β‐catenin signaling has been associated with the loss of dopaminergic neurons in the substantia nigra in PD.^[^ ^152^ ^]^ Given the involvement of Wnt/β‐catenin signaling in the pathogenesis of these neurodegenerative diseases, targeting and modulating this signaling pathway has emerged as a potential therapeutic approach.^[^ ^132^ ^]^

Certain herbal plants have the potential to alleviate the symptoms of PD. Studies have shown that herbal plants such as the extract of Mucuna pruriens (Mp) can reduce the levels of nitric oxide (NO) and lipid peroxidation induced by paraquat in PD. This demonstrates that Mp protects dopaminergic neurons in the substantia nigra against NO‐induced damage.^[^ ^153^ ^]^ Furthermore, the herbal plant Withania somnifera (Ws) protects dopaminergic neurons in the substantia nigra against MB‐PQ‐induced PD by regulating oxidative stress and apoptotic mechanisms.^[^ ^154^ ^]^ Overall, these findings highlight the potential therapeutic benefits of herbal plants such as Mp and Ws in mitigating symptoms and protecting against neurodegeneration in patients with PD.

Gastrointestinal dysfunction is common in patients with PD and is the initial symptom before the onset of motor symptoms.^[^ ^155^ ^]^ Neuroinflammation is considered a factor in the pathogenesis of PD and potentially affects the dysbiosis of the gut microbiota.^[^ ^156^ ^]^ Compared to healthy individuals, changes in the gut microbiota in patients with PD include a decrease in the genera *Bacteroides fragilis* and *Roseburia* and in the family *Ruminococcaceae*, as well as an increase in the families *Lactobacillaceae*, *Akkermansia*, and *Bifidobacterium*,^[^ ^157^ ^]^ which lead to a pro‐inflammatory state and are associated with the recurrent gastrointestinal symptoms of PD patients.^[^ ^158^ ^]^

Researchers found that transferring the gut microbiota from normal mice to PD mice using fecal microbiota transplantation (FMT) reduced the expression of the TLR4/TNF‐α signaling pathway molecules in the gut and brain, as well as the content of SCFAs in PD mice, resulting in improved symptoms.^[^ ^159^ ^]^ Therefore, the gut microbiota can play a neuroprotective role by inhibiting the expression of the TLR4/TNF‐α signaling pathway via the microbiota–gut–brain axis.^[^ ^160^ ^]^ PD model mice receiving fecal transplants from healthy mice exhibit improved motor function, increased neurotransmitters in the striatum, and reduced neuroinflammation.^[^ ^160a^ ^]^ Healthy mice receiving fecal transplants from patients with PD exhibit worsened motor function and reduced neurotransmitters levels in the striatum.^[^ ^161^ ^]^ Clinical case reports of patients with PD receiving FMT have shown improvement in leg tremors and other PD symptoms, with improved constipation lasting for up to 3 months after surgery.^[^ ^162^ ^]^ There is a possible link between the gut microbiota and the pathogenesis of PD because patients with PD exhibit dysbiosis of the gut microbiota, and probiotics can improve symptoms.^[^ ^163^ ^]^

OP and osteopenia are common in patients with PD, particularly in older populations. Patients with PD show a higher prevalence of insufficiency or reduction in 25‐hydroxyvitamin D (25(OH)D) than patients with AD or healthy controls.^[^ ^164^ ^]^ Studies suggest that several mechanisms could contribute to the loss of bone density in patients with PD.^[^ ^165^ ^]^ Patients with PD exhibit elevated levels of α‐synuclein in their bloodstream and the presence of Lewy bodies in the midbrain and enteric terminal nerves. Based on this, Figueroa and Rosen hypothesized that the aggregation of α‐synuclein may lead to changes in the cellular dynamics of α‐synuclein within bone cells, potentially contributing to bone dysfunction in individuals with PD.^[^ ^166^ ^]^ Furthermore, the gut microbiota can influence inflammation and hormone regulation, leading to bone loss and an increased risk of OP and osteopenia.^[^ ^165^, ^167^ ^]^ Analysis via a multivariate methodology revealed that women with PD had a markedly lower BMD of 2.1% less than the others in the study.^[^ ^166^ ^]^

**Figure 7:**
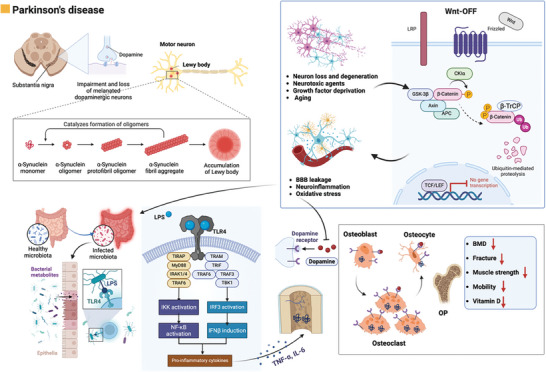
The brain–gut–bone axis may play a significant role in PD. PD is characterized by the permanent loss of dopaminergic neurons in the substantia nigra and cholinergic neurons in the meynert basal nucleus in the central nervous system. It is also characterized by the accumulation and aggregation of α‐synuclein in affected neurons, leading to the formation of Lewy bodies and Lewy neurites. Negative regulators of Wnt signaling, such as oxidative stress, inflammation, neurotoxic agents, growth factor deprivation, or aging, antagonize the Wnt/β‐catenin signaling in neurons. In this state of Wnt‐OFF, excess β‐catenin is rapidly phosphorylated by GSK‐3β at the APC/axin/GSK‐3β destruction complex and subsequently undergoes ubiquitin‐proteasomal degradation. Consequently, the transcription of Wnt target genes involved in neuron survival is inhibited. Neuroinflammation is considered a contributing factor to the pathogenesis of PD and may potentially affect the dysbiosis of gut microbiota. The gut microbiota can exert a neuroprotective role by inhibiting the expression of the TLR4/TNF‐α signaling pathway through the microbiota–gut–brain axis. PD patients exhibit higher circulating levels of α‐synuclein and the presence of Lewy bodies in the midbrain and enteric terminal nerves. It is postulated that α‐synuclein aggregates could lead to alterations in the cellular dynamics of α‐synuclein in bone cells, potentially contributing to bone impairment in PD patients. Individuals diagnosed with PD commonly experience symptoms such as decreased BMD, increased fracture risk, insufficient levels of vitamin D, and OP. Figures were created with BioRender.com.

The brain–gut–bone axis also plays a role in PD, however, its exact function and relationship are still being investigated. Several mechanisms, including α‐synuclein pathology, autonomic dysfunction, neuroinflammation, gut microbiota and metabolites, and intestinal permeability have been proposed to clarify the involvement of the brain–gut–bone axis in PD (**Figure** **7**). In summary, further research is needed to elucidate the precise functions and relationships of the brain–gut–bone axis in PD. Understanding these mechanisms can provide insights into disease progression, potential therapeutic targets, and the development of novel treatment strategies.

### 3.3 Huntington's Disease

HD is a genetic disorder characterized by the expansion of cytosine–adenine–guanine trinucleotide repeats in the huntingtin gene (HTT), leading to the expression of a mutant form of HTT.^[^ ^168^ ^]^ The clinical manifestations of HD include chorea, psychiatric disorders, and progressive dementia, which is a covert, slow, and progressive process. The occurrence and severity of symptoms are influenced by environmental factors including diet, physical activity, and stress. Several dysregulated ubiquitin/proteasome and autophagy systems contribute to the development of HD.^[^ ^169^ ^]^ Additionally, abnormalities in Wnt/β‐catenin signaling have been implicated in the aggregation of mutant HTT protein and neuronal cell death in HD. In contrast to other neurodegenerative diseases, inhibiting Wnt/β‐catenin signaling proves advantageous in the treatment of HD.^[^ ^132^, ^170^ ^]^ Therefore, the alteration of Wnt/β‐catenin signaling is considered a promising therapeutic target for the treatment of neurodegenerative diseases. Currently, no disease‐modifying treatments for HD have been approved.^[^ ^171^ ^]^

There is an imbalance in the gut microbiota metabolism in HD. An HD R6/2 mouse model showed increased intestinal permeability, decreased tight junction protein expression, and disordered intestinal microflora. Compared with wild‐type mice, R6/2 mice had a higher relative abundance of *mycobacterium* and a lower relative abundance of *firmicutes*.^[^ ^172^ ^]^ Compared with the wild‐type control group, the microbiota composition of 12‐week‐old HD mice was significantly different, with an increase in *bacterioid* and a decrease in *firmicus*, and the microbial diversity of male HD mice was increased. However, there was no difference in the diversity of female HD mice.^[^ ^173^ ^]^ The production of the protective SCFA butyrate in the gut increases with an increase in the butyrate substitution pathway.^[^ ^174^ ^]^ Clinical studies showed that there were significant differences in β diversity of HD intestinal microbiota, and α diversity decreased significantly.^[^ ^175^ ^]^ Patients with HD exhibit metabolic imbalances in the gut microbiota, which can lead to increased intestinal permeability and decreased expression of tight junction proteins.^[^ ^176^ ^]^ Therefore, change in the intestinal microbiota and its function is one of the factors influencing HD.

Muscle atrophy in the skeletal system is believed to have a significant impact on HD pathogenesis.^[^ ^177^ ^]^ In the late phase of HD, obvious reductions in muscle mass, such as sarcopenia,^[^ ^178^ ^]^ and reductions in bone demineralization, such as OP. Body composition alterations is prevalent in HD and closely associated with disease progression. Studies have demonstrated that striatal degeneration becomes apparent during the premanifest stages of HD and is associated with BMD.^[^ ^179^ ^]^ BMD and T‐scores were lower in patients on HD. The initial phases of bone and muscle wasting are important clinical signs that allow for timely and effective treatment to prevent disability and fragility fractures.^[^ ^180^ ^]^ Bone mass, lean body mass, and fat mass could be used as early and reliable prognostic indices, in agreement with other studies.^[^ ^180^ ^]^

**Figure 8:**
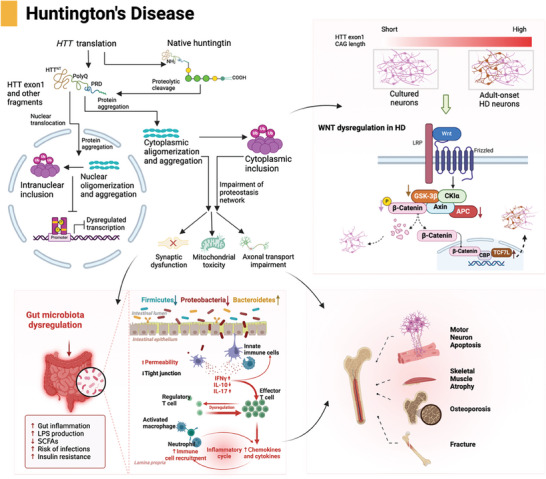
Regulation mechanisms of HD by the brain–gut–bone axis. The HTT gene produces both the full‐length huntingtin protein and an amino‐terminal HTT exon1 fragment, resulting from abnormal splicing. The full‐length huntingtin protein undergoes proteolytic cleavage, generating additional protein fragments. These fragments enter the nucleus and are retained through self‐association, oligomerization, and aggregation. Simultaneously, huntingtin fragments also oligomerize and aggregate in the cytoplasm. The aggregation of huntingtin is worsened by the disease‐related impairment of the proteostasis network, leading to widespread cellular dysfunction. The abnormal forms of huntingtin cause various cellular impairments, including synaptic dysfunction, mitochondrial toxicity, and a decrease in axonal transport rate. In HD, the Wnt/β‐catenin signaling pathway is dysregulated. Frizzled receptors are upregulated, while members of the β‐catenin destruction complex, particularly APC and GSK3β, are downregulated. Additionally, GSK‐3β phosphorylation inhibits the Wnt/β‐catenin signaling in HD. Metabolic imbalances in the gut microbiota are observed in HD patients, resulting in increased intestinal permeability and reduced expression of tight junction proteins. HD is associated with an imbalance in gut microbiota metabolism, with an increase in Bacteroides and a decrease in *Firmicutes*. Furthermore, HD mice display increased microbial diversity. During the advanced stages of HD, significant muscle mass reduction, including muscle atrophy and sarcopenia, becomes apparent. Additionally, there is a decrease in bone mineral density, which can lead to OP. HTT: huntingtin gene; polyQ: polyglutamine; GSK3β: glycogen synthase kinase 3β; APC: APC regulator of Wnt signaling pathway. Figures were created with BioRender.com.

In conclusion, HD is a neurodegenerative disorder in which the function and relationship of the brain–gut–bone axis is being explored. Bidirectional communication between the brain, gut, and bone, is disrupted in HD and illustrated in **Figure** **8**. Pathological changes in the brain can affect gut functions, including motility, permeability, and the ability of the gut to regulate immune responses. The gut microbiota is involved in nutrient metabolism and absorption, and its dysregulation in HD may further affect nutritional status and bone health. Alterations in the gut can affect brain function and contribute to disease progression. The mechanism behind gut microbiota and bone loss in HD is not yet fully understood but may involve dysregulation of the brain–gut–bone axis, inflammation, and changes in hormone levels.

### 3.4 Amyotrophic Lateral Sclerosis

ALS is a neurodegenerative disease characterized by progressive muscle paralysis.^[^ ^181^ ^]^ As the most common motor neuron disease in adults, ALS causes progressive muscle weakness and, ultimately, respiratory failure due to degeneration of the upper and lower motor neurons.^[^ ^182^ ^]^ ALS pathology arises from an intricate combinations of molecular mechanisms, including neuroinflammation, impaired RNA metabolism, oxidative stress, mitochondrial dysfunction, disruptions of cytoskeletal integrity, altered exon splicing, impaired nucleocytoplasmic and axonal transport, accumulation of toxic protein aggregates, disturbances in autophagy, and excitotoxicity caused by excessive glutamate levels.^[^ ^183^ ^]^ To date, mutations in ≈30 genes have been documented to trigger ALS.^[^ ^183a^ ^]^ After decades of research and more than 120 clinical trials,^[^ ^184^ ^]^ only two therapeutic medicines have been approved by the Food and Drug Administration. A modest slowdown of disease progression was achieved with riluzole^[^ ^185^ ^]^ and edaravone.^[^ ^183a^ ^]^ Motor neurons, astrocytes, microglia, and oligodendrocytes exhibit elevated levels of Wnt/β‐catenin signaling, leading to the impairment of the neuromuscular junction in individuals with ALS. The clusters of acetylcholine receptors (AchR) formation holds significant importance in the development of the neuromuscular junction.^[^ ^132^, ^186^ ^]^

An imbalance in the composition of intestinal microbiota may be an environmental factors contributing to the development of ALS.^[^ ^187^ ^]^ Research has demonstrated that the levels of several butyricogenic bacteria that are important for intestinal completion and inflammation regulation are lower in patients with ALS.^[^ ^188^ ^]^ Experimental evidence using ALS‐susceptible *Sod1* transgenic mice has demonstrated that *Akkermansia muciniphila* can improve the symptoms of ALS, whereas *Ruminococcus* and *Desulfovibrio* exacerbate the disease.^[^ ^189^ ^]^

Neurological diseases may lead to rapid loss of BMD due to physical inactivity and reduced muscle contractions.^[^ ^190^ ^]^ Musculoskeletal functional deterioration in ALS is associated with an increase in pathological bone fractures (induced by low‐energy traumatic events) owing to a decrease in bone density.^[^ ^191^ ^]^ Among other factors, ALS may be associated with malnutrition and changes in energy expenditure, which may lead to further muscle fiber loss and bone abnormalities.^[^ ^192^ ^]^ Morini et al. investigated the bone health status in a sample of ALS inpatients attending neurorehabilitation using BMD and trabecular bone score and confirmed the hypothesis that ALS patients may exhibit deteriorated bone health with lower bone density.^[^ ^192b^ ^]^

**Figure 9:**
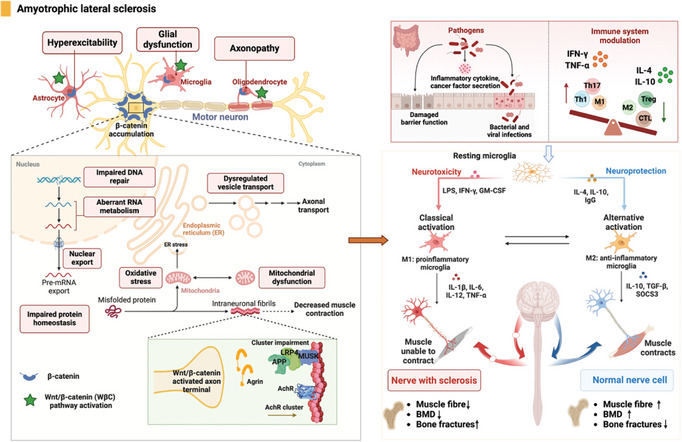
Schematic of regulation mechanisms of ALS through the brain–gut–bone axis. ALS develops due to a complex interplay of molecular mechanisms, including neuroinflammation, disrupted RNA metabolism, oxidative stress, mitochondrial dysfunction, disturbances in cytoskeletal integrity, altered exon splicing, impaired nucleocytoplasmic and axonal transport, accumulation of toxic protein aggregates, disruptions in autophagy, and excitotoxicity caused by excessive glutamate. In ALS, the Wnt/β‐catenin signaling pathway is upregulated. Microglia, astrocytes, and oligodendrocytes contribute to demyelination, inflammation, and dysfunction of the neuromuscular junction in motor neurons. The formation of clustered AchR is crucial for the proper functioning of the neuromuscular junction. Elevated Wnt/β‐catenin signaling impairs the Agrin‐LRP4‐MUSK pathway, leading to impaired AchR clustering at the neuromuscular junction. Toxins and neuroactive metabolites produced by damaged intestinal epithelial barriers or enteric bacteria can cross the BBB, enter the systemic circulation, and impact ALS pathogenesis. Alternatively, microbial metabolic products may indirectly affect the central nervous system through modulation of the immune system. Peripheral immune T lymphocytes play a role in regulating the fate of microglia, which in turn influences neuron degeneration or survival. Th1, Th17, and GM‐CSF producing CD4^+^ T lymphocytes promote a neurotoxic M1‐like phenotype in microglia, while Th2, Treg, and certain CD8^+^ T cell types may contribute to the promotion of a neurosupportive M2‐like phenotype. ALS is associated with musculoskeletal functional deterioration, which includes an increased risk of pathological bone fractures due to loss of BMD. Malnutrition, changes in energy expenditure, and other factors may also be associated with ALS and contribute to muscle fiber loss and bone abnormalities. AchR: aryl hydrocarbon receptor; MuSK: muscle, skeletal receptor tyrosine‐protein kinase; LRP4: low‐density lipoprotein receptor‐related protein 4; BBB, blood–brain barrier. Figures were created with BioRender.com.

As mentioned above, chronic neuroinflammation is prevalent in patients with ALS and may be related to the occurrence and development of the disease. ALS has also been associated with dysbiosis of the gut microbiota, which may contribute to inflammation and bone loss in ALS patients. **Figure** **9** has briefly clarified the connection between the brain, gut, and bone in the progress of ALS. However, the exact underlying mechanism is not fully understood. Therefore, elucidating its relationship with the brain–gut–bone axis may lead to new therapeutic approaches for ALS.

### 3.5 Multiple Sclerosis

MS is an autoimmune disease affecting the brain and spinal cord. It is a neurodegenerative, inflammatory disease of the CNS. Globally, MS affects ≈2.8 million people, including the young. It is also a leading cause of disability among young people.^[^ ^193^ ^]^ It is commonly associated with symptoms such as tingling sensations, limb weakness, balance problems, fatigue, dizziness, vision disorders, and dysesthesia.^[^ ^194^ ^]^ MS is a chronic autoimmune demyelinating disease of the CNS characterized by inflammation and white matter lesions composed of astrocytes, microglia, and activated immune cells.^[^ ^195^ ^]^ There is a breakdown of the unique BBB formed by endothelial cells in the CNS of patients with MS. The formation of the barrier in the gut immune system relies on the Wnt/β‐catenin pathway, and inhibiting this pathway in endothelial cells prior to the disease onset leads to more severe symptoms in mice due to increased infiltration of immune cells into the CNS.^[^ ^196^ ^]^ Consequently, reactivation of the Wnt/β‐catenin signaling pathway partially restores endothelial function and inhibits the infiltration of immune cells into the CNS in the context of MS.^[^ ^132^ ^]^

There is an altered gut microbiota in individuals with MS.^[^ ^197^ ^]^ A comparison of the gut microbiota in fecal samples of patients with MS and healthy individuals revealed that patients with MS have abundant *Pseudomonas*, *Chlamydia*, *Hemophilus*, *Brucella*, *Dorea*, *Enterobacter*, and *Bacteroides*, and are less abundant in *Prevotella*, *Parabacteroides*, *Adlercreutzia*, *Collinsella*, *Lactobacillus*, *Bifidobacterium*, and *Hemophilus*.^[^ ^198^ ^]^ Russian patients with MS showed a significant increase in the relative abundance of the genera *Ruminococcus* and unclassified *Ruminococcaceae*.^[^ ^199^ ^]^ These results indicate the presence of abnormal intestinal microbiota in patients with MS, which can be improved through intestinal microbiota intervention measures such as FMT, which may benefit patients with MS.^[^ ^200^ ^]^ In one case series, a patient with secondary progressive MS with recurrent *Clostridium difficile* infection received a single FMT treatment, which resolved recurrent *Clostridium difficile* infection and prevented MS disease progression for over 10 years; repeated FMT also improved MS symptoms in three patients.^[^ ^201^ ^]^ Hence, improving dysbiosis of the gut microbiota through the intake of probiotics, prebiotics, and FMT can alleviate systemic inflammation, regulate metabolic balance, and thereby treat MS.

MS‐associated gut dysbiosis primarily exacerbates OP by disrupting intestinal permeability and causing low‐grade systemic inflammation.^[^ ^202^ ^]^ Patients with MS have an increased risk of fragility fractures.^[^ ^203^ ^]^ Primary hip fragility fractures were significantly more common in patients with MS than in the matched controls. Following an initial fracture, patients with MS exhibited a significantly higher rate of falls but were more likely to be diagnosed with OP and treated with medications.^[^ ^204^ ^]^ A control study evaluated the BMD of 91 MS patients with disease progression for at least 10 years and found that OP was present in 44.0% of the patients.^[^ ^205^ ^]^ The etiology of OP in individuals with MS is multifactorial and includes a lack of weight‐bearing exercise secondary to disability, chronic inflammation, and glucocorticoid‐induced bone loss.^[^ ^206^ ^]^

**Figure 10:**
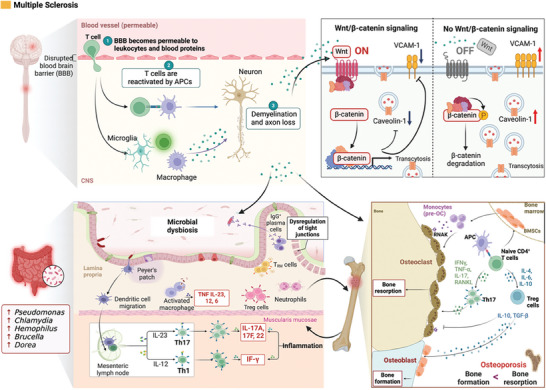
Schematic diagram summarizing the roles of the brain–gut–bone axis in MS. MS is a chronic autoimmune disease that affects the CNS and is characterized by inflammation and the formation of white matter lesions involving astrocytes, microglia, and activated immune cells. Dysregulation of the BBB increases its permeability, allowing neutrophil infiltration into the CNS. In the absence of Wnt ligands, inflammatory cytokines in the CNS promote the high expression of Caveolin‐1, which regulates endothelial transcytosis, and VCAM‐1, which facilitates the interaction of immune cells with blood vessels in the CNS. Activation of the Wnt/β‐catenin signaling pathway in endothelial cells during the peak of EAE inhibits the expression of Caveolin‐1 and VCAM‐1, thereby reducing the infiltration of CD4^+^ T cells into the CNS. Autoimmune diseases like MS may be associated with gut dysbiosis, increased intestinal permeability, microbial translocation, and local and systemic inflammation. Inflammatory mediators, such as TNF‐α, can decrease the expression of tight junction proteins, leading to increased permeability of the intestinal barrier. Experimental evidence suggests that interventions aimed at shifting immune responses toward immunoregulatory pathways, involving regulatory cells that produce anti‐inflammatory cytokines like IL‐10, TGF‐β, or IL‐35, can restore immune homeostasis and protect against inflammatory demyelination in the CNS. The gut microbiota influences bone remodeling by modulating the balance between Th17 and Treg cells. Th17 cells promote the differentiation of osteoclasts, leading to increased bone resorption. On the other hand, Treg cells inhibit osteoclastogenesis and enhance bone formation by secreting anti‐inflammatory cytokines such as IL‐4, IL‐10, and TGF‐β. VCAM‐1: vascular cell adhesion molecule‐1; CNS: central nervous system; EAE: experimental autoimmune encephalomyelitis; CD: cluster of differentiation; Th: T helper; IL: interleukin; TNF: tumor necrosis factor; TGF: transforming growth factor. Figures were created with BioRender.com.

In summary, MS may alter the composition and diversity of gut microbiota. These changes can affect immune regulation and systemic inflammation, potentially influencing the progression and severity of MS. Patients with MS are at a higher risk of developing OP and fractures. The brain–gut–bone axis exhibits complex interactions in MS and it has been shown in **Figure** **10**. Further research is needed to elucidate the specific functions and relationships of the brain–gut–bone axis in MS.

Overall, the investigation of the function and relationship of the brain–gut–bone axis in neurodegenerative diseases is an emerging area of research. The brain–gut–bone axis exhibits complex interactions in neurodegenerative diseases. Although our understanding is still developing, various factors, such as neuroinflammation, gut microbiota dysbiosis, intestinal permeability, and bone health, have been investigated to shed light on their roles in neurodegenerative diseases. Further research is needed to elucidate the specific functions and relationships of the brain–gut–bone axis in neurodegenerative diseases. Understanding these mechanisms can provide insights into disease progression, potential therapeutic targets, and the development of interventions to manage MS and its associated complications.

## 4 Therapeutics for Maintaining Brain–Gut–Bone Axis Balance

In‐depth research on the molecular mechanisms of the brain–gut–bone axis is important for understanding the pathological mechanisms of neurodegenerative diseases, developing new treatment approaches, and formulating preventive strategies. Possible approaches to intervene in and manage neurodegenerative diseases include modulating the gut microbiota, enhancing skeletal health, implementing therapeutic strategies to regulate neuroinflammatory responses, and preserving bone health. However, further research is needed to enhance our understanding of the brain–gut–bone axis in neurodegenerative diseases.

### 4.1 Intestinal Microbiota Regulation and Remodeling

#### 4.1.1 Probiotics and Prebiotics Modulation

Probiotics are primarily active microorganisms that are beneficial to human health when administered in sufficient quantities.^[^ ^207^ ^]^ In contrast, prebiotics are nondigestible and fermentable food components that promote the growth of beneficial microorganisms and/or facilitate the beneficial regulation of microbial metabolites.^[^ ^208^ ^]^ A diet rich in prebiotics, such as galacto‐oligosaccharides, fructo‐oligosaccharides, insulin, and oligofructose, may modulate the microbiota ecosystem.^[^ ^209^ ^]^ Currently, some probiotics, including *Lactobacillus*, *Bifidobacteria*, *Reuteria*, have been widely used in daily industrial and agricultural production, food, and medicine.^[^ ^210^ ^]^ Probiotics and prebiotics can positively influence the composition and function of gut microbiota, leading to improvements in gastrointestinal symptoms, inflammation, and cognitive function in certain populations. In particular, individuals who are obese and have neurodegenerative diseases may benefit from these interventions, potentially mitigating some of the symptoms associated with these conditions, as they have proven regulatory effects on the gut–brain axis.^[^ ^211^ ^]^

Research on the effects of probiotics and prebiotics has revealed that their intake can restore gut microbial homeostasis and improve the behaviors or diseases related to the regulation of the brain–gut–bone axis. Recent research indicates that intervention with prebiotic fibers can lead to the production of beneficial metabolites in individuals with PD and can induce changes in the gut microbiota.^[^ ^212^ ^]^ In participants with PD, prebiotic intervention has shown good tolerability and safety, and it is associated with beneficial changes in the gut microbiota, SCFA levels, inflammation, and neurofilament light chain, thereby improving related clinical symptoms.^[^ ^212^ ^]^ A study showed that the long‐term administration of a probiotic composed of six specific bacterial strains alleviated motor impairments in a genetic mouse model of PD and exerted neuroprotective effects on dopaminergic neurons.^[^ ^213^ ^]^ Another study showed that the probiotic *Bifidobacterium* breve CCFM1067 may prevent or treat PD by modulating the microbiota–gut–brain axis and can improve motor impairments, dopaminergic neuron death, and neurotransmitter depletion caused by MPTP in mice.^[^ ^214^ ^]^ The combination of Probio‐M8 therapy with conventional approaches has shown enhanced efficacy in the clinical treatment of PD.^[^ ^215^ ^]^ Therefore, probiotic supplementation and nutritional interventions may emerge as promising therapeutic approaches to prevent the progression of PD.

Studies have shown that the daily intake of prebiotics and probiotics can delay neurocognitive decline and reduce the risk of developing AD. The supplementation of *Lactobacillus plantarum* can reshape and regulate the gut microbiota and glucose metabolism and alleviate amyloid protein accumulation and cognitive impairments in AD rats.^[^ ^216^ ^]^ The study reported a significant improvement in cognitive impairment in AD mice after a 10‐week intervention with the probiotic *Lactobacillus plantarum* DP189, evidenced by a significant increase in serum levels of 5‐HT, dopamine, and GABA. Additionally, it reduced neuronal damage, Aβ deposition, and tau protein pathology associated with microtubules, demonstrating its beneficial effect on AD.^[^ ^217^ ^]^ Additionally, *Lactobacillus plantarum MTCC1325* demonstrated the ability to alleviate behavioral impairments in an AD rat model, reduce cerebral Aβ deposition, and increase acetylcholine levels.^[^ ^218^ ^]^ After a 12‐week probiotic intervention, the experimental group receiving probiotics supplementation showed significant improvements in learning and memory abilities compared to the control group.^[^ ^219^ ^]^ Compared with single strains, supplementation with multistrain probiotic formulations has shown more pronounced effects on reshaping the gut microbiota in AD rats, particularly by increasing the diversity of the gut microbial community and its community structure.^[^ ^220^ ^]^ Therefore, modulating the gut microbiota to alleviate brain inflammation and affect neurotransmitter production may have a therapeutic effect on AD and has broad potential applications.^[^ ^221^ ^]^

Recent studies have indicated that gut microbiota may be associated with the occurrence and progression of ALS disease. The strain *Akkermansia muciniphila*, which is associated with nicotinamide, significantly slows disease progression and prolongs the survival of ALS‐prone mice.^[^ ^189^ ^]^ Furthermore, studies have shown that *Lacticaseibacillus rhamnosus* HA‐114 exhibits neuroprotective effects in *Caenorhabditis elegans* models of ALS and HD. It has been suggested that impaired lipid metabolism plays a role in neurodegeneration, and dietary intervention with *L. rhamnosus* HA‐114 restores lipid homeostasis and energy balance by promoting mitochondrial β‐oxidation. These findings support the investigation of interventions derived from *L. rhamnosus* HA‐114 to modify the progression of neurodegenerative diseases.^[^ ^222^ ^]^ These findings represent the initial step in comprehensively understanding the potential impact of microbiota on ALS. In the future, methods aimed at altering microbiota composition may be employed to develop novel approaches for the treatment of ALS.

HD is characterized by gastrointestinal symptoms that significantly affect the patients' quality of life. Some studies have demonstrated the neuroprotective effects of *Lacticaseibacillus rhamnosus* HA‐114 in *C. elegans* models of HD.^[^ ^222^ ^]^ Various probiotic strains, including *Lactobacillus* and *Bifidobacterium*, have been extensively studied for their potential neuroprotective effects in animal models of neurodegenerative diseases such as HD.^[^ ^102^ ^]^ Specifically, microorganisms that have shown promising results include *Lactobacillus rhamnosus*, *Lactobacillus reuteri*, *Lactobacillus casei*, *Lactobacillus acidophilus*, *Bifidobacterium*, and *Saccharomyces*.^[^ ^223^ ^]^ Considering the clinical symptoms, dysbiosis of the gut microbiota, and positive results of probiotics and other gut interventions for similar neurodegenerative diseases, the potential of the gut as a therapeutic target in HD must be further explored.

An increasing body of evidence suggests that the gut microbiota can serve as a therapeutic target for MS. In a study involving patients with MS, the administration of probiotics was found to enhance the presence of specific taxonomic groups, such as *Lactobacillus species*, which are known to be diminished in individuals with MS.^[^ ^224^ ^]^ Additionally, the abundance of other taxonomic groups associated with MS, including *Akkermansia* and *Blautia species*, was reduced following probiotic supplementation.^[^ ^224^ ^]^ Preclinical studies have shown that the administration of probiotics can significantly reduce the incidence and delay the progression of MS. These effects are achieved by modulating immune and inflammatory markers and altering the composition of the gut microbiota.^[^ ^225^ ^]^ Furthermore, probiotic supplementation has been shown to improve in associated motor impairments. *Bifidobacterium* increases the expression of tight junction proteins, thereby enhancing the intestinal epithelial barrier function in patients with MS.^[^ ^226^ ^]^ A substantial body of evidence from studies conducted in mice and humans indicates that the microbiota plays a significant role in the pathogenesis and progression of MS.^[^ ^227^ ^]^ Therefore, interventions aimed at controlling the gut microbiota and correcting gut dysbiosis hold potential as therapeutic approaches for MS.

In addition, the gut microbiota has the potential to regulate bone health. Probiotic supplements can increase bone density in healthy individuals and prevent primary (estrogen deficiency‐related) and secondary OP.^[^ ^228^ ^]^ Recent investigations have provided evidence for the positive effects of probiotic supplementation on bone metabolism. In a randomized controlled trial, the addition of *Lactobacillus reuteri* ATCC PTA 6475 effectively mitigated bone loss in older women with low bone mineral density.^[^ ^229^ ^]^ A study reported that probiotic supplementation led to increased BMD in postmenopausal women.^[^ ^230^ ^]^ Another study showed that probiotics can improve bone density and reduce the risk of OP in animal models by regulating immune cells and inflammatory factors in the gut.^[^ ^231^ ^]^ These studies suggest that probiotics and probiotic supplements may be a promising approaches for improving bone health and preventing neurodegenerative diseases and OP by modulating the gut microbiota. Changes in the gut microbiota after probiotics supplementation in neurodegenerative diseases are consistent with varying degrees of improvement, as shown in **Table** **1**.

**Table 1:** Examples of changes in the composition of gut microbiota and therapeutics with neurodegenerative diseases.

Disease	GM alterations associated with the disease	Probiotic therapy used	Effects of probiotic therapy in studies	Refs.
PD	Increase: *Proteus, Selenomonas, Tenericutes, Christensenellaceae, Tissierellaceae* Decrease: *Bifidobacteriaceae, Lachnospiraceae, Lactobacillaceae, Pasteurellaceae, Verrucomicrobiaceae*, *Prevotellaceae*	Fermented milk, containing multistrain probiotic and prebiotic fiber	Brain: increased neurotransmitters in the striatum, reduced neuroinflammation, ameliorates dopaminergic neuron death and neurotransmitter depletion Gut: beneficial metabolites production, induced changes in the gut microbiota Bone: improved motor function, decreased risk of OP and osteopenia	[160, 167, 212, 214]
		Multistrain probiotic containing *Lactobacillus acidophilus*, *Bifidobacterium bifidum*, *Lactobacillus reuteri*, and *Lactobacillus fermentum*		
AD	Increase: *Bacteroides, Actinomyces, Ruminant coccus, Proteus, Selenomonas, Tenericutes* Decrease: *Ruminococcus, butyricococcus, Lactobacillus, Bifidobacterium, Firmicutes, Verrucobacteria, Proteobacteria*, and *Actinomyces*	Multistrain probiotic containing *L. acidophilus, L. casei, B. bifidum, L. fermentum*	Brain: improved the deposition of β‐amyloid plaques, Tau protein pathology, glial reactions, and cognitive impairment in the brain Gut: remodeling and regulating gut microbiota and glucose metabolism Bone: secretion of leptin and adiponectin leading to changes in bone mass and density	[89, 139, 216]
		Multistrain probiotic (*Lactobacillus acidophilus, Bifidobacterium bifidum*, and *Bifidobacterium longum*)		
		*Acetobacter aceti, Acetobacter sp., Lactobacillus delbrueckii, Lactobacillus fermentum, Lactobacillus fructivorans, Enterococcus faecium, Leuconostoc spp., Lactobacillus kefiranofaciens, Candida famata*, and *Candida krusei*		
HD	Increase: *Bacteroides, β‐diversity* Decrease: *Firmicutes, α‐diversity*	*Lactobacillus, Bifidobacterium*, *Lacticaseibacillus rhamnosus HA‐114*	Brain: play a neuroprotective role; reduce striatal degeneration Gut: stimulate the intestinal microbiota to produce mucin to prevent the introduction of pathogens, enhance the immune regulation of the intestinal system, and inhibit the production of bacterial toxins Bone: increase muscle mass reduction, prevent fragility fractures	[128, 180, 222, 298]
ALS	Increase: *Bacteroidetes, Dorea, Citrobacter*, *Eubacterium eligens* Decrease: *Oscillibacter, Anaerostipes, Lachnospira, Ruminococcus, and Subdoligranulum genera*	*Streptococcus thermophilus, Lactobacillus fermentum*, *Lactobacillus delbrueckiisubsp, Lactobacillus plantarum, and Lactobacillus salivarius*	Brain: play a neuroprotective role, improve symptoms of ALS; promote mitochondrial β oxidation to restore lipid homeostasis and energy balance Gut: improved their capabilities to counteract gut pathogens and their anti‐inflammatory properties and restoring gut barrier Bone: improve BMD and trabecular bone score	[187, 192, 222, 299]
MS	Increase: *Psuedomonas, Mycoplana, Haemophilus, Blautia, and Dorea* Decrease: *Parabacteroides, Prevotella, Adlercreutzia, Collinsella, Erysipelotrichaceae*	*VLS #3, Lactobacillus paracasei, Bifidobacterium animalis, E. coli Nissle 1917, and Prevotella histicola*	Brain: alleviate systemic inflammation, regulate metabolic balance Gut: synthesizing nutrients and vitamins, strengthening the intestinal barrier, and regulating the immune system to reduce inflammation Bone: alleviating OP, reduces fragility fractures and related sports injuries	[201, 202, 225, 300]

In summary, probiotics and prebiotics exert positive effects on neurodegenerative diseases by regulating the gut microbiota, enhancing gut barrier function, modulating neuroinflammation, improving cognition, and enhancing bone health. These mechanisms contribute to the maintenance of immune homeostasis and potentially alleviate immune dysregulation associated with various diseases within the brain–gut–bone axis.

#### 4.1.2 Fecal Microbiota Transplantation

Currently, multiple therapeutic approaches targeting the gut microbiota have been proposed, with FMT being a research hotspot. FMT is the process of transferring feces from a healthy donor to the gut of a recipient to restore damaged gut microbiota.^[^ ^232^ ^]^ Transplanting feces rich in SCFAs (especially butyrate) leads to microbial remodeling, increases the number of *lactobacilli*, and improves the gut microbiota.^[^ ^233^ ^]^ FMT is now considered an effective method for regulating gut microbiota and preventing the progression of clinical diseases.

FMT can be used to treat CNS diseases, such as PD. Patients with PD who received FMT showed improvement in leg tremors and other PD symptoms as well as relief from constipation during their 12‐week follow‐up.^[^ ^162^ ^]^ In an AD animal experiment using a germ‐free AD mouse model, transplantation of both normal and AD gut microbiota caused an increase in pathology, with the latter being more significant.^[^ ^139^ ^]^ Emerging evidence suggests that FMT may be a potential therapeutic approach for treating neurodegenerative diseases, including HD.^[^ ^223^ ^]^ Clinical studies and randomized controlled trials have provided evidence that FMT from healthy donors to patients with MS results in improved motor symptoms and halts disease progression in MS patients.^[^ ^201^, ^234^ ^]^ A groundbreaking clinical trial investigating FMT in patients with ALS has been conducted. This trial provides valuable insights into the biological effects of FMT in ALS and offers preliminary clinical data regarding the potential of microbiota‐based treatment approaches as novel therapeutic targets for ALS.^[^ ^235^ ^]^

Probiotic/prebiotic therapy and FMT can not only enhance gut barrier function and regulate intestinal motility, but also increase bone density, preventing primary (estrogen deficiency) and secondary OP.^[^ ^13b^ ^]^ Therefore, creating a healthy gut environment based on gut microbiota therapy has far‐reaching implications for brain health, peripheral metabolism, and preventing bone‐related diseases. It can also promote an auxiliary therapeutic role in neurodegenerative diseases by regulating the brain–gut–bone axis.

#### 4.1.3 Dietary Interventions

Recent research suggests that the effects of diet on the modulation of neuroinflammatory and neurodegenerative mechanisms contribute to the development of some of the most prevalent chronic CNS diseases.^[^ ^236^ ^]^ Dietary interventions can increase the production of SCFAs, affect body weight, composition, and glucose homeostasis, reduce neuroinflammation, regulate the gut microbiota, and reduce the incidence of neurodegenerative diseases. A prospective study of a large cohort of elderly individuals free of dementia at baseline provided evidence that those with lower calorie intake had a reduced risk of developing AD.^[^ ^237^ ^]^

Insoluble dietary fiber provides more fermentable fiber in the gut, which favors the abundance of SCFA‐producing bacteria such as *Prevotella* and *Lactobacillus*.^[^ ^238^ ^]^ Additionally, the dietary fiber provided by a high‐fiber diet is mainly fermented by *Firmicutes* and other bacteria, thereby increasing the production of SCFAs such as acetate, propionate, and butyrate.^[^ ^239^ ^]^ Consumption of olive oil rich in omega‐6 polyunsaturated fatty acids has been associated with increased SCFA production, whereas a greater intake of n6 polyunsaturated fatty acids (n6‐PUFA) has been negatively associated with the development of AD. Omega‐3 polyunsaturated fatty acids (n3‐PUFA) obtained from fish are of interest because of their ability to modulate intestinal communities.^[^ ^240^ ^]^ In addition, complex polysaccharides are fermented by the microbiota into SCFAs, which contribute to decreased neuroinflammation by inhibiting HDAC signaling and GPCR signaling and lowering systemic inflammation.^[^ ^22^ ^]^

Plant‐based dietary nutrients mainly include dietary fiber, antioxidants, and vitamins A, C, and E, and are usually low in fat and simple sugars. PD prevalence is significantly lower in people of different ethnicities after consuming plant‐based diets.^[^ ^241^ ^]^ Accordingly, individuals following a vegan or vegetarian diet, or those on a Mediterranean diet, have higher levels of SCFAs; these diets are also beneficial for bone health.^[^ ^242^ ^]^

Adequate calcium and vitamin D intake through diet or supplements is important for bone health and can have protective and therapeutic effects against neurodegenerative diseases by regulating the immune system and promoting anti‐inflammatory responses.^[^ ^243^ ^]^ The body requires extra calcium for sufficient raw material for bone formation.^[^ ^244^ ^]^ Vitamin D promotes calcium absorption in the intestine; therefore, vitamin D supplementation provides sufficient calcium to maintain bone structure.^[^ ^245^ ^]^ Moreover, vitamin D and calcium interact with each other to regulate the immune system and induce anti‐inflammatory responses.^[^ ^246^ ^]^

Vitamin K significantly affects the nervous system. Recent research has indicated that vitamin K2 holds great promise for the treatment of AD by preventing neuronal cell apoptosis, oxidative stress, and microglial activation through its role in electron transfer.^[^ ^247^ ^]^ These findings contribute to the growing body of research that suggests a protective association between dietary intake of vitamin K and age‐related cognitive decline.^[^ ^120^ ^]^ In addition to its protective effects on cognitive function, vitamin K2 has also shown significant inhibitory effects on inflammation and α‐synuclein fibrillation in PD.^[^ ^119^ ^]^ A clinical study provided evidence that patients with PD exhibit significantly lower levels of serum vitamin K2. Consequently, vitamin K2 has been proposed to be associated with the onset and progression of PD and to serve as a biomarker for the diagnosis and prognosis of PD.^[^ ^248^ ^]^ Vitamin K has also been suggested for the treatment of MS to reduce intensity or slow disease progression.^[^ ^118^ ^]^ An observational study linked lower levels of vitamin K2 to a higher frequency of MS attacks in individuals diagnosed with MS.^[^ ^249^ ^]^ Hence, vitamin K2 has been demonstrated to be associated with the progression of PD, AD, and MS, indicating its significant potential as a treatment for age‐related neurodegenerative disorders.

Insufficient levels of vitamins D and K resulting from malnutrition or lack of sunlight exposure, along with compensatory hyperparathyroidism, heightened bone resorption, low BMD, and an elevated risk of falls, may contribute to an increased susceptibility to hip fractures in individuals with AD. The effectiveness of three interventions, namely, sunlight exposure, menatetrenone supplementation, and risedronate in combination with calcium and/or vitamin D supplementation in preventing hip fractures in patients with AD has been shown.^[^ ^250^ ^]^ The majority of patients with OP are older than 50 years of age. This is because the degeneration of bone tissue accelerates bone loss, making the bones of older people generally weaker than those of younger people.^[^ ^251^ ^]^ Falls can easily increase fracture risk; therefore, it is necessary to implement measures to prevent falls in the elderly.

**Figure 11:**
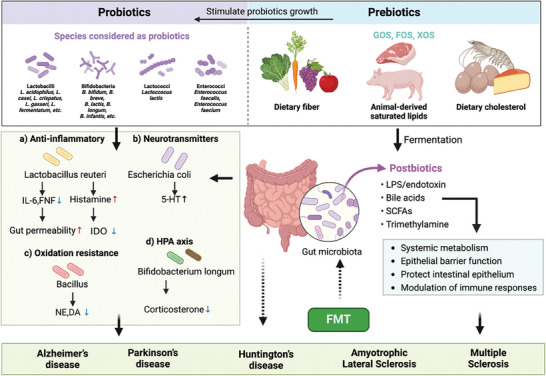
Diagram illustration of probiotics, prebiotics, and FMT therapeutic strategies for intestinal microbiota regulation and remodeling in neurodegenerative diseases. Probiotics and prebiotics can promote the growth of beneficial gut bacteria, which may reduce gut inflammation and improve intestinal barrier function. FMT involves transferring fecal matter from a healthy donor to a patient's gut in order to restore a healthy gut microbiota profile and improve gut function, which may also have anti‐inflammatory effects and improve neurological function. These strategies aim to restore a healthy balance of gut microbiota, which may have anti‐inflammatory effects and promote the production of beneficial neurotransmitters in the brain. Figures were created with BioRender.com.

In summary, gut microbiota has become a therapeutic target for the treatment of neurodegenerative diseases. Gut microbiota intervention measures mainly included probiotics, prebiotics, FMT, and dietary interventions (**Figure** **11**). Dietary nutrients not only regulate the gut microbiota but also improve the metabolism of gut microbiota metabolites. These metabolic changes can enhance brain cognition in neurodegenerative diseases, such as AD. Additionally, supplementation with specific nutrients can improve bone density and quality in patients with neurodegenerative diseases, thereby addressing conditions such as osteoporosis and fractures. These findings further elucidate the therapeutic potential of targeting the brain–gut–bone axis for the treatment of neurodegenerative diseases.

### 4.2 Skeletal System Improvement

#### 4.2.1 Exercise Regulates BDNF Expression in the Brain through the Secretion of Skeletal Muscle Factors

Exercise is an important nonpharmacological approach to regulate BDNF expression, playing a positive role in promoting learning and memory, and improving neurodegenerative diseases.^[^ ^252^ ^]^ Exercise can increase BDNF mRNA levels in multiple regions of the brain, primarily in the hippocampus, leading to hippocampal cell proliferation and improved cognitive function.^[^ ^253^ ^]^ Exercise protects the CNS; however, until recently, it was found to depend on the endocrine capacity of the skeletal muscle.^[^ ^254^ ^]^ During exercise, the metabolic demands of the skeletal muscle stimulate the liver to synthesize and release ketone bodies, such as acetoacetate and d‐β‐hydroxybutyrate, into the bloodstream, which crosses the BBB and accumulates in the hippocampus, where it stimulates histone acetylation at BDNF promoters, leading to increased BDNF expression.^[^ ^255^ ^]^ Physical performance enhances cognitive function and memory, which are mainly mediated by increased BDNF levels in the brain.^[^ ^256^ ^]^

Reduced regenerative capacity of hippocampal neurons and decreased BDNF levels are commonly observed in patients with moderate‐to‐severe AD.^[^ ^257^ ^]^ However, exercise can increase BDNF levels and improve cognitive impairments in patients with AD.^[^ ^258^ ^]^ Therefore, exercise plays a crucial role in promoting BDNF expression and improving cognition. A controlled clinical trial demonstrated that a multimodal physical exercise program effectively reduced the risk of falls and led to improvements in gait, balance, and bone mineral density in the short and medium term among institutionalized patients with AD.^[^ ^259^ ^]^ Exercise science studies have confirmed that scientific exercise interventions can promote higher brain functions; delay neurological dysfunction,^[^ ^260^ ^]^ improve learning memory, reduce the risk of disease in patients with AD,^[^ ^261^ ^]^ optimize memory capacity, gait, and muscle strength, and improve locomotor performance in patients with PD^[^ ^262^ ^]^ and HD.^[^ ^263^ ^]^

Exercise induces the production of various skeletal muscle factors including irisin, cathepsin B, and IGF‐1. These factors enter the brain from the periphery via the bloodstream and regulate BDNF expression, promoting learning and memory. The hormone irisin, released during muscle contraction, is a key regulatory factor in enhancing cognitive function in the brain through exercise, and holds promise for the treatment of cognitive decline caused by aging or AD.^[^ ^264^ ^]^ Furthermore, patients with moderate‐to‐severe AD show reduced levels of FNDC5/irisin in the brain and cerebrospinal fluid, whereas high FNDC5/irisin levels in the brain or periphery improve synaptic and memory impairments in AD mouse models.^[^ ^265^ ^]^ Therefore, certain exercise intensity is a prerequisite for elevating peripheral irisin levels in the bloodstream.

Cathepsin B, a myokine, exerts potent neuroprotective effects. Cathepsin B is a promising therapeutic targets for delaying the onset and progression of cognitive impairment.^[^ ^266^ ^]^ Four weeks of exercise has been shown to increase the peripheral levels of cathepsin B in healthy young individuals and is associated with improved hippocampal‐dependent memory function. Cathepsin B can cross the BBB and upregulate BDNF, thus promoting cognitive function in the brain.^[^ ^267^ ^]^ IGF‐1 is an important factor that promotes growth and neurogenesis and regulates energy metabolism in the body. Exercise can increase IGF‐1 levels and positively affect learning and memory by regulating BDNF expression.^[^ ^268^ ^]^ Hence, exercise can regulate cognitive function in the brain by increasing the levels of skeletal muscle factors such as irisin and cathepsin B in the bloodstream, which transmit information to the brain.

#### 4.2.2 Exercise Regulates Muscle Metabolites and Affects Cognitive Function

Exercise has been found to have a significant impact on both muscle metabolites levels and cognitive function. Exercise‐induced metabolites, such as β‐hydroxybutyrate, lactate, and potentially α‐ketoglutarate, regulate BDNF expression, and improve cognitive function. β‐hydroxybutyrate not only serves as an energy substrate but also has therapeutic effects in neurodegenerative disorders.^[^ ^269^ ^]^ Sleiman et al. discovered that the fatty acid metabolite β‐hydroxybutyrate, generated through exercise, can regulate BDNF expression via HDAC2/3, thereby enhancing cognitive function.^[^ ^255^ ^]^ El Hayek et al. found that lactate generated during exercise can regulate BDNF expression through SIRT1, thereby improving learning and memory in the brain.^[^ ^270^ ^]^ In addition to β‐hydroxybutyrate and lactate, α‐ketoglutarate is also believed to play a role in regulating BDNF expression.^[^ ^145^ ^]^ In addition to factors derived from skeletal muscles, energy metabolites can regulate BDNF expression in the brain through peripheral pathways.^[^ ^256^ ^]^ Understanding these mechanisms may facilitate the development of targeted interventions to enhance brain health and alleviate neurodegenerative disorders.

Exercise provides a new direction for exploring neuroinflammatory activity in AD by directly and indirectly regulating the immune response of the CNS and promoting hippocampal neurogenesis.^[^ ^271^ ^]^ A well‐designed exercise intervention has been shown to promote higher brain functions, delay neurofunctional impairments,^[^ ^260^ ^]^ and improve learning and memory in patients with AD.^[^ ^261^ ^]^ It can also optimize memory, gait, and muscle strength in patients with PD^[^ ^262^ ^]^ and improve motor abilities in HD patients.^[^ ^272^ ^]^

Modulating muscle signals in the CNS by regulating muscle factors and metabolites may help combat age‐related neurodegeneration and brain diseases influenced by systemic signals.^[^ ^273^ ^]^ Increasing studies have suggested that impaired skeletal muscle homeostasis affects brain metabolism and physiology. Understanding the complex interactions between skeletal muscles and the brain may lead to more effective therapeutic strategies for an expanded healthy lifespan and brain disease prevention.^[^ ^274^ ^]^

In conclusion, exercise exerts a positive influence on muscle metabolites, which in turn affect cognitive function. The release of metabolites, such as lactate, ATP, and BDNF, during exercise contributes to improved brain health, neuroplasticity, and cognitive performance. Regular physical activity can have beneficial effects on both muscle and brain health, emphasizing the importance of exercise as a holistic approach to maintaining cognitive function.

#### 4.2.3 Exercise Improves Cognition and Health by Regulating the Gut Microbiota

Exercise plays a role in regulating the gut microbiota, which, in turn, regulates various life activities. Dong et al. found that after training, divers experienced an increase in *Lactobacilli* and *Bifidobacteria* levels, accompanied by significant changes in the diversity of the gut microbiota.^[^ ^275^ ^]^ Different exercise intensities have distinct effects on the gut microbiota. Low‐to‐moderate intensity exercise interventions can improve the abundance of beneficial gut bacteria, promote gut homeostasis, and exert beneficial effects on overall health.^[^ ^276^ ^]^

The gut microbiota and its metabolites can activate sensory neurons in the gut, transmitting information to the brain and leading to an exercise‐induced increase in dopamine levels in the striatum, enhancing motivation for physical activity. Supplementation with the gut microbiota or its metabolites can improve exercise capacity in mice.^[^ ^277^ ^]^ Exercise can regulate the gut microbiota by modulating the vagus nerve‐mediated HPA axis.^[^ ^278^ ^]^ Aerobic exercise can enhance VN activity by regulating gut metabolites diversity.^[^ ^279^ ^]^ Exercise promotes the production of SCFAs via gut microbiota metabolism, thereby enhancing the information transmission effect of the vagus nerve,^[^ ^280^ ^]^ suggesting that exercise‐mediated neural communication between the ENS and CNS can occur both through the direct regulation of VN activity and indirect effects mediated by gut microbiota metabolites and neurotransmitters.

Furthermore, exercise can promote SCFAs secretion and increase the levels of anti‐inflammatory cytokine IL‐10 by regulating the gut microbiota, thereby reducing the production of inflammatory factors induced by lipopolysaccharides and enhancing the body's anti‐inflammatory capacity, thereby promoting the health of patients with MS.^[^ ^281^ ^]^ Therefore, the gut microbiota produces a large number of metabolites, mainly neurotransmitters and SCFAs, which in turn influence the physiology and behavior of the brain.

The gut microbiota also influences skeletal health.^[^ ^94^ ^]^ Exercise regulates the gut microbiota, improving cognition and behavior, while the gut microbiota affects bone regulation and the development of skeletal diseases, such as OP and inflammatory joint diseases characterized by bone loss.^[^ ^66^ ^]^ The gut microbiota also regulates bone mass likely mediated through its effects on the immune system, which in turn regulates the formation of OC.^[^ ^282^ ^]^ The gut microbiota may serve as a new therapeutic target for the prevention of OP and fractures.

An increase in gut microbiota diversity, reduction in pathogenic bacterial populations, and improvement in exercise capacity can effectively reduce the risk of neurodegenerative diseases. Overall, these three factors create a beneficial cycle, where exercise inhibits pathogenic bacteria and promotes the growth of beneficial bacterial populations, which, in turn, secrete various factors that are beneficial to the brain and skeletal system, continuously improving physical performance.

Therefore, we believe that exercise plays a regulatory role, through the brain–gut–bone axis, in improving overall body function, enhancing cognition, and promoting skeletal health in patients with neurodegenerative diseases. However, the mechanism by which exercise‐mediated gut microbiota regulates neurological function remains unclear, and exploring the mechanism of exercise‐mediated gut microbiota regulation of neurological function is of great significance for clarifying the mechanism of exercise intervention for neurodegenerative diseases.

### 4.3 Emerging and Promising Therapeutic Modalities

#### 4.3.1 Microbial‐Directed Approaches

Antibiotics are closely associated with changes in the gastrointestinal microbiota. Alterations in the gut microbiota can induce changes in brain activity, increasing the potential for therapeutic manipulation of the microbiota in AD and other neurological disorders.^[^ ^126^ ^]^ A small (*n*  =  14), unblinded, nonrandomized clinical study showed that treatment with rifaximin, a nonabsorbable antibiotic, improved motor fluctuations in patients with PD.^[^ ^283^ ^]^ Minocycline has a beneficial effect on the gut microbiota and exhibits a neuroprotective role in PD.^[^ ^284^ ^]^ Minocycline has shown neuroprotective properties in experimental models of ALS, PD, HD, and MS.^[^ ^285^ ^]^

Sodium oligomannate (GV‐971), a marine‐derived oligosaccharide, is a novel agent that may improve cognition in patients with AD.^[^ ^286^ ^]^ Additionally, a study found that GV‐971 significantly reduced the accumulation and aggregation of α‐synuclein in Prnp‐SNCAA53T mice when administered in the relatively early stages of the disease. Furthermore, GV‐971 corrected the inhibitory effect of α‐synuclein‐induced neuronal extracellular vesicle release, thereby providing neuroprotection.^[^ ^287^ ^]^ By targeting the brain–gut axis, regulating the imbalanced gut microbiota, reshaping immune homeostasis, reducing brain inflammation, and improving cognitive function, it is possible to achieve therapeutic effects in the treatment of AD.^[^ ^288^ ^]^

Novel therapeutic strategies based on the modulation of the gut microbiota within the brain–gut–bone axis have shown promise for the treatment of neurodegenerative diseases. By regulating the gut microbiota, it is possible to influence neuroinflammatory responses, improve gut function, and positively impact brain health. This comprehensive treatment approach offers an innovative avenue with the potential to improve the symptoms and quality of life in patients with neurodegenerative diseases. However, further research and clinical trials are necessary to confirm its safety and efficacy and to provide more support and guidance for its integration into clinical practice.

#### 4.3.2 Treatments Targeting the Osteoblasts

Recently, the relationship between neurodegenerative diseases and skeletal disorders has received increasing attention. Epidemiological studies have shown that patients with AD or PD are more prone to developing OP. Early detection of reduced bone density in patients with PD through active treatment can help reduce disability rates, extend the lifespan, and improve the prognosis of patients with AD and PD. Studies have shown that extracellular vesicles derived from young OBs can enter the brain and improve cognitive function in AD mice. Extracellular vesicles derived from young OBs modulate the bone–brain axis to exert anti‐AD effects, revealing a novel bone–brain information exchange mechanism that provides new targets and perspectives for AD prevention and treatment.^[^ ^289^ ^]^

Osteocalcin is a noncollagen protein produced by OBs. Changes in the levels of osteocalcin secreted by OBs may be associated with age‐related cognitive decline.^[^ ^290^ ^]^ Osteocalcin can alleviate CNS lesions, protect damaged neurons, and mitigate central lesions in AD mice by regulating lipid metabolism.^[^ ^291^ ^]^ Osteocalcin binds to midbrain neurons and promotes the production of dopamine neurotransmitters,^[^ ^292^ ^]^ suggesting a relationship between PD and bone metabolism. The levels of osteocalcin in the cerebrospinal fluid of PD model rats are lower than those in the control group. Direct injection of osteocalcin into the right striatum of PD rats improves motor dysfunction induced by 6‐hydroxydopamine and reduces the depletion of dopamine neurons through the AKT/gsk3β signaling pathway.^[^ ^293^ ^]^ Additionally, osteocalcin can improve PD symptoms by reducing the expression of inflammatory factors and inhibiting the proliferation of astrocytes and microglia.^[^ ^294^ ^]^

Furthermore, osteocalcin influences PD via gut microbiota; Hou et al. showed osteocalcin‐induced alteration in the gut microbiota of PD mice compared with that of the control group, with a significant reduction in *Firmicutes* and *Bacteroidetes*. Osteocalcin improved motor defects and dopaminergic neuron loss in PD mice. Modulation of the gut microbiota and increased propionic acid levels may be potential mechanisms underlying the neuroprotective effects of osteocalcin in PD.^[^ ^295^ ^]^

Therefore, osteocalcin can improve the gut microbiota composition in PD mice and may have a therapeutic effect on patients with PD. In the future, it will be necessary to further investigate the mechanisms of osteocalcin in neurodegenerative diseases and provide more experimental evidence for the use of osteocalcin as a potential peripheral target for the treatment of neurodegenerative diseases.

#### 4.3.3 Combining Brain–Gut–Bone Targeted Approaches

Combining microbiota‐targeted approaches (such as prebiotics, probiotics, dietary adjustments, or FMT) with traditional treatments for neurodegenerative diseases can provide synergistic benefits in multiple aspects of these diseases. For example, microbiota interventions can be used in conjunction with drug therapies, such as tetrabenazine, to treat chorea with HD.^[^ ^296^ ^]^

Microbiota‐targeted approaches can complement emerging disease‐modifying therapies by combining targeted microbiota interventions with traditional HD treatments. This can improve treatment outcomes by modulating the composition and function of the gut microbiota, reducing neuroinflammation, enhancing neurotransmitter production and signaling, and promoting neuronal survival and function.^[^ ^297^ ^]^ Furthermore, exploring the combination of gut microbiota‐targeted therapy with foundational therapy and therapies targeting the skeletal system to further improve motor and cognitive symptoms, may be worthwhile. Therefore, combination therapies may control motor, cognitive, and psychiatric symptoms and improve the quality of life of patients, as conventional treatment methods often fail to manage these symptoms adequately.^[^ ^223^ ^]^

While studying the heterogeneity and complexity of the gut microbiota poses challenges, advancements in microbiome research have provided valuable insights into potential interactions between the gut, brain, and skeletal systems in patients with neurodegenerative disease. These findings have led to the development of novel treatment approaches, such as prebiotics, probiotics, dietary adjustments, and FMT, which play a crucial role in modulating the gut microbiota to improve symptoms and enhance the quality of life. Further research is required to enhance our knowledge and translate these findings into effective and safe clinical applications.

Based on the above discussion, the gut microbiota has emerged as a target for bacterial‐ or cellular‐based therapies. The most promising areas of research are FMT and probiotics and prebiotics are promising adjunctive treatments. Probiotics and prebiotics create healthy gut environments by promoting the proliferation and metabolism of beneficial bacteria. Clinical trials have demonstrated that probiotics, prebiotics, and FMT can modulate the composition of the gut microbiota and improve neurological disorders. Furthermore, external influences in daily life, such as exercise, diet, smoking, and alcohol consumption habits, can affect the gut microbiota, exacerbating or ameliorating symptoms and progression of neurodegenerative diseases. One potential approach to alleviate dysbiosis is to adopt a more comprehensive dietary strategy that provides adequate nutrition to promote healthy and diverse gut microbiota.

**Figure 12:**
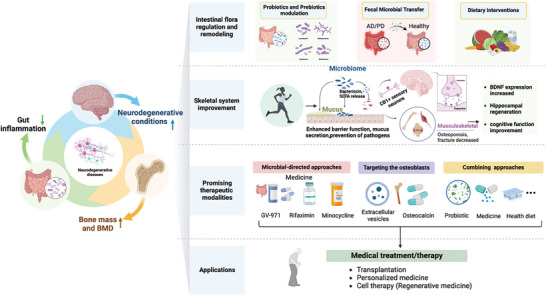
Therapeutics for neurodegenerative diseases with brain–gut–bone axis balance maintaining, which combined with intestinal microbiota regulation and remodeling (probiotics, prebiotics, and FMT), skeletal system improvement (exercise) and emerging and promising therapeutic modalities. The integration of a nutritious diet with additional interventions like FMT, exercise, and probiotics has the potential to influence the gut microbiota and decelerate the advancement of neurodegenerative diseases. It will offer new strategies for improving and treating these diseases with the balance of brain–gut–bone axis.

In summary, combining a healthy diet with other forms of intervention, such as FMT, exercise, probiotics, antibiotics, and osteocalcin may affect the gut microbiota and slow the progression of neurodegenerative diseases (**Figure** **12**). Therefore, the concept of the brain–gut–bone axis provides a novel perspective for understanding neurodegenerative diseases, aiding in the identification of underlying causes and offering new strategies for diseases prevention and treatment.

## 5 Challenges and Outlooks

Neurodegenerative diseases present a formidable global health challenge marked by the gradual deterioration of nerve cells and the subsequent manifestation of cognitive and motor impairments. These include AD, PD, HD, and MS, among others. The pathogenesis of these diseases is complex and involves the disruptions of multiple physiological systems. To date, there is a lack of comprehensive reviews exploring the intricate relationship between the brain–gut–bone axis and its potential for the development of groundbreaking therapies in the treatment of neurodegenerative disorders.

In this context, our review proposes a hypothesis regarding the involvement of the brain–gut–bone axis in neurodegenerative diseases. The brain–gut–bone axis is a complex network that connects the brain, gut, and skeletal systems, facilitating dynamic information exchange through various means, such as neuronal pathways, the immune system, endocrine regulation, and metabolic systems. The importance of this axis has been increasingly recognized as it has profound implications for the occurrence and development of neurodegenerative diseases. Amidst this complex landscape, the brain–gut–bone axis has emerged as a crucial network orchestrating the modulation of multiple physiological systems and garnering significant attention for its profound impact on the onset and progression of neurodegenerative diseases.

Specifically, the brain–gut–bone axis plays a crucial regulatory role in neurodegenerative diseases. First, it enables interactions between the brain, gut, and bone through neuronal pathways. The complex connection between the nervous system (including the VN and ANS) and the gut involves communication with the gut microbiota, inflammation, and skeletal nerves. Disruption of this communication can lead to increased inflammation, gut permeability, and neuronal damage, accelerating the progression of neurodegenerative diseases. Second, the immune system plays an important regulatory role within the brain–gut–bone axis, which is important for the development of neurodegenerative diseases and the control of inflammatory responses. Endocrine regulation is a key component of the brain–gut–bone axis. Hormones and neurotransmitters transmit signals between the brain, gut, and skeletal systems through axis regulation. This endocrine regulation is essential for maintaining overall metabolic balance, skeletal health, and normal functioning of the nervous system. Finally, the metabolic system plays an important role in the brain–gut–bone axis. The gut microbiota influences brain function and skeletal health by secreting metabolic products. Metabolic regulation is associated with the occurrence and development of neurodegenerative diseases.

The brain–gut–bone axis plays a crucial role in maintaining health and influencing disease development, particularly through interactions between intestinal microbes and the host gut microecology. It is becoming increasingly evident that neurodegenerative diseases are not solely confined to the brain but are associated with an elevated risk of OP and bone fractures, accompanied by changes in bone mass and density. This suggests a close relationship between dysregulation of the intestinal microecological environment, bone system, and conditions such as AD, PD, HD, MS, and other neurological disorders.

In recent years, there has been a growing focus on unraveling the interconnectedness involved in the pathogenesis of neurodegenerative diseases and developing rational and effective intervention strategies. A key area of exploration is gut microbiota, which has provided further insights into potential targets within the brain–gut–bone axis that may influence the onset and progression of these diseases. By understanding the intricate interplay between the brain, gut microbiota, and bone system, researchers aim to identify novel therapeutic approaches that can modulate this axis and mitigate the impact of neurodegenerative diseases. This emerging research trend holds promise for advancing our understanding of these complex disorders and for developing targeted interventions that could improve patient outcomes.

In addition, this review delves into the therapeutic strategies aimed at preserving the equilibrium of the brain–gut–bone axis. These strategies include interventions targeting brain health, intestinal microbiota modulation, and skeletal system well‐being. The objective of neurodegenerative disease treatments is to restore balance within the brain–gut–bone axis by employing a combination of interventions such as the regulation and reshaping of intestinal microbiota through the use of probiotics, prebiotics, and FMT. Enhancing the skeletal system through exercise and incorporating emerging therapeutic approaches are some of these strategies. By implementing these interventions, there is a potential for positive effects on the gut microbiota and the potential to slow down the progression of neurodegenerative diseases.

In conclusion, the study of the brain–gut–bone axis provides us with a novel perspective that helps us to better understand the mechanisms underlying the occurrence and development of neurodegenerative diseases. By exploring the interactions between various components of the axis, we can seek new therapeutic strategies to maintain axis balance and provide new insights for the prevention and treatment of neurodegenerative diseases. This is of importance in clinical practice and for the overall health of patients. However, research on the brain–gut–bone axis in neurodegenerative diseases still faces challenges. First, the current understanding of the complex mechanisms and interactions within the axis is still limited, requiring further research to establish causal relationships between the gut microbiota and neurodegenerative diseases. Second, the existing treatment methods mainly focus on specific aspects of the axis, necessitating the exploration of more comprehensive and effective treatment approaches. Moreover, no drugs have been developed to comprehensively target the entire gut–brain–bone axis owing to the involvement of multiple complex physiological processes and interactions. In addition, the safety and efficacy of the treatments needs to be evaluated further. It is difficult to control for factors such as medication and diet, and the causes of neurological disorders are extremely complex and vary across different age groups and disease stages. Although new approaches such as FMT and probiotics have been proposed, FMT requires strict aseptic procedures, and there are challenges regarding the specificity of probiotic strains, optimal dosages, and intervention timing. Controlling and managing neurodegenerative diseases has becomes more complicated owing to factors such as medications, diet, age groups, and disease stages.

Therefore, measures must be implemented to address these challenges. First, research on the mechanisms and interactions of the brain–gut–bone axis must be strengthened to better understand its role in neurodegenerative diseases. Second, treatment methods targeting the entire axis, including combination therapy and multidimensional interventions, must be further developed and studied. Finally, more in‐depth clinical trials are needed to evaluate the safety and efficacy of treatments and to determine the optimal treatment approach and dosage. Therefore, it is essential to strengthen research on the mechanisms and interactions of the brain–gut–bone axis, develop treatment methods targeting the entire axis, and conduct in‐depth clinical trials to assess its safety and efficacy.

In summary, this review discusses the important role of the brain–gut–bone axis in the prevention and treatment of neurodegenerative diseases. By providing in‐depth insights, this study emphasizes the significance of considering the interrelationships among the brain, gut microbiota, and skeletal system in managing these complex diseases. Future research efforts should focus on deepening our understanding of the brain–gut–bone axis, developing comprehensive treatment approaches, and conducting rigorous clinical trials to optimize treatment strategies and improve the prognosis of patients with neurodegenerative disease.

## 6 Conclusion

Neurodegenerative diseases represent a significant clinical challenge that affects millions of people worldwide. There is an urgent need for continued research to advance our understanding of the pathogenesis and mechanisms of neurodegenerative diseases and to develop effective therapies to improve the clinical outcomes and quality of life of affected individuals. Considering this, our review aimed to propose a hypothesis regarding the triangular relationship between the brain, gut, and bone systems and to investigate its potential in the advancement of pioneering treatments for neurodegenerative disorders.

By means of dynamic communication along the brain–gut–bone axis, the gut microbiota collaborates with the brain and bone systems in four major ways: by modulating the immune response, impacting metabolism, regulating endocrine processes, such as hormones, neuropeptides, and neurotransmitters, and directly affecting neurons and neural signaling. Imbalances in the brain–gut–bone axis have been implicated in the pathology and development of various neurodegenerative diseases, including AD, PD, HD, ALS, and MS, as well as in gut microbiota dysbiosis and musculoskeletal imbalances. Advances in our understanding of the underlying mechanisms of the brain–gut–bone axis have paved the way for the development of therapeutic strategies for maintaining balance. In addition to the pharmacological treatments, healthy diet therapy with other interventions (e.g., FMT, probiotics, and prebiotics) may affect the gut microbiota and slow the progression of neurodegenerative diseases. In general, a healthy mindset and balanced diet may be better able to prevent or reduce symptoms in patients with AD and PD. A combination of reasonable exercise interventions can be considered to improve intestinal microecology and bone healthy, which in turn affects the neurotransmission in the brain–gut–bone axis, improves neurological function, and suppresses neurodegenerative diseases. Our proposed concept of the brain–gut–bone axis may offer valuable insights and guidance for the treatment of neurodegenerative disorders.

In conclusion, targeting the brain–gut–bone axis has enormous potential for the treatment of neurodegenerative diseases. However, further research and exploration are needed to find better treatment methods and ensure their safety and efficacy. A better understanding of the brain–gut–bone axis and its role in neurodegenerative diseases will continue to guide the development of novel treatments and interventions, offering hope for improved outcomes and quality of life for affected individuals. Undoubtedly, this intriguing concept will be further explored in the future.

## Conflict of Interest

The authors declare no conflict of interest.

## Author Contributions

R.L., Z.M., and Y.L. contributed equally to this work. All authors have read and approved the article. Drafting of the manuscript: R.L., M.Z., and Y.L.; Revision of the manuscript for important intellectual content: X.C. and H.W.; Obtained funding: J.S. and J.C.; Supervision: J.S. and J.C.

## References

[1] D. M. Wilson , M. R. Cookson , L. Van Den Bosch , H. Zetterberg , D. M. Holtzman , I. Dewachter , Cell 2023, 186, 693. 36803602 10.1016/j.cell.2022.12.032

[2] D. M. Teleanu , A. G. Niculescu , I. I. Lungu , C. I. Radu , O. Vladâcenco , E. Roza , B. Costăchescu , A. M. Grumezescu , R. I. Teleanu , Int. J. Mol. Sci. 2022, 23, 5938. 35682615 10.3390/ijms23115938 PMC9180653

[3] L. Culig , X. Chu , V. A. Bohr , Ageing Res. Rev. 2022, 78, 101636. 35490966 10.1016/j.arr.2022.101636 PMC9168971

[4] P. Sun , L. Su , H. Zhu , X. Li , Y. Guo , X. Du , L. Zhang , C. Qin , Microorganisms 2021, 9, 2281. 34835406 10.3390/microorganisms9112281 PMC8621510

[5] S. Yoshikawa , K. Taniguchi , H. Sawamura , Y. Ikeda , A. Tsuji , S. Matsuda , Metabolites 2022, 12, 1052. 36355135 10.3390/metabo12111052 PMC9692629

[6] a) L. Y. Tan , X. Y. Yeo , H. G. Bae , D. P. S. Lee , R. C. Ho , J. E. Kim , D. G. Jo , S. Jung , Life 2021, 11, 698; 34357070 10.3390/life11070698 PMC8305650

[7] S. Miri , J. Yeo , S. Abubaker , R. Hammami , Front. Microbiol. 2023, 14, 1098412. 36733917 10.3389/fmicb.2023.1098412 PMC9886687

[8] a) A. Rousseaud , S. Moriceau , M. Ramos‐Brossier , F. Oury , Horm. Mol. Biol. Clin. Invest. 2016, 28, 69; 10.1515/hmbci-2016-0030 27626767

[9] E. Otto , P. R. Knapstein , D. Jahn , J. Appelt , K. H. Frosch , S. Tsitsilonis , J. Keller , Int. J. Mol. Sci. 2020, 21, 4946. 32668736 10.3390/ijms21144946 PMC7404044

[10] C. Li , G. Pi , F. Li , Front. Cell Infect. Microbiol. 2021, 11, 579323. 33777828 10.3389/fcimb.2021.579323 PMC7994858

[11] Y. W. Zhang , Y. J. Li , P. P. Lu , G. C. Dai , X. X. Chen , Y. F. Rui , Food Funct. 2021, 12, 5703. 34048514 10.1039/d0fo03468a

[12] a) Y. Cheng , C. Chen , F. Zhang , Ageing Res. Rev. 2023, 85, 101857; 36669690 10.1016/j.arr.2023.101857

[13] a) N. G. Vallianou , E. Geladari , D. Kounatidis , J. Cardiovasc. Med. 2020, 21, 83; 10.2459/JCM.0000000000000900 31809283

[14] L. H. Morais , H. L. Schreiber , S. K. Mazmanian , Nat. Rev. Microbiol. 2021, 19, 241. 33093662 10.1038/s41579-020-00460-0

[15] M. Eisenstein , Nature 2016, 533, S104. 27191486 10.1038/533S104a

[16] J. F. Cryan , K. J. O'Riordan , C. S. Cowan , K. V. Sandhu , T. F. Bastiaanssen , M. Boehme , M. G. Codagnone , S. Cussotto , C. Fulling , A. V. Golubeva , Physiol. Rev. 2019, 99, 1877. 31460832 10.1152/physrev.00018.2018

[17] Y. Kakinuma , Neurochem. Int. 2021, 143, 104934. 33307153 10.1016/j.neuint.2020.104934

[18] X. J. Li , X. Y. You , C. Y. Wang , X. L. Li , Y. Y. Sheng , P. W. Zhuang , Y. J. Zhang , CNS Neurosci. Ther. 2020, 26, 783. 32472633 10.1111/cns.13401 PMC7366750

[19] E. A. Mayer , K. Tillisch , A. Gupta , J. Clin. Invest. 2015, 125, 926. 25689247 10.1172/JCI76304 PMC4362231

[20] E. A. Wehrwein , H. S. Orer , S. M. Barman , Regulation 2016, 37, 125.

[21] C. R. Martin , V. Osadchiy , A. Kalani , E. A. Mayer , Cell. Mol. Gastroenterol. Hepatol. 2018, 6, 133. 30023410 10.1016/j.jcmgh.2018.04.003 PMC6047317

[22] B. D. Needham , R. Kaddurah‐Daouk , S. K. Mazmanian , Nat. Rev. Neurosci. 2020, 21, 717. 33067567 10.1038/s41583-020-00381-0

[23] J. B. Furness , Nat. Rev. Gastroenterol. Hepatol. 2012, 9, 286. 22392290 10.1038/nrgastro.2012.32

[24] a) M. M. Kaelberer , K. L. Buchanan , M. E. Klein , B. B. Barth , M. M. Montoya , X. Shen , D. V. Bohórquez , Science 2018, 361, 5236; 10.1126/science.aat5236 PMC6417812 30237325

[25] D. V. Bohórquez , R. A. Shahid , A. Erdmann , A. M. Kreger , Y. Wang , N. Calakos , F. Wang , R. A. Liddle , J. Clin. Invest. 2015, 125, 782. 25555217 10.1172/JCI78361 PMC4319442

[26] Y. El‐Hakim , S. Bake , K. K. Mani , F. Sohrabji , Neurobiol. Dis. 2022, 165, 105627. 35032636 10.1016/j.nbd.2022.105627

[27] F. Leblhuber , D. Ehrlich , K. Steiner , S. Geisler , K. Kurz , Nutrients 2021, 13, 361. 33504065 10.3390/nu13020361 PMC7912578

[28] E. Stavropoulou , E. Bezirtzoglou , Front. Immunol. 2020, 11, 2192. 33072084 10.3389/fimmu.2020.02192 PMC7544950

[29] Y. Obata , Á. Castaño , S. Boeing , A. C. Bon‐Frauches , C. Fung , T. Fallesen , M. G. de Agüero , B. Yilmaz , R. Lopes , A. Huseynova , Nature 2020, 578, 284. 32025031 10.1038/s41586-020-1975-8

[30] B. Dalile , L. Van Oudenhove , B. Vervliet , K. Verbeke , Nat. Rev. Gastroenterol. Hepatol. 2019, 16, 461. 31123355 10.1038/s41575-019-0157-3

[31] L. Galland , J. Med. Food 2014, 17, 1261. 25402818 10.1089/jmf.2014.7000 PMC4259177

[32] F. De Vadder , E. Grasset , L. Mannerås Holm , G. Karsenty , A. J. Macpherson , L. E. Olofsson , F. Bäckhed , Proc. Natl. Acad. Sci. USA 2018, 115, 6458. 29866843 10.1073/pnas.1720017115 PMC6016808

[33] B. Niesler , S. Kuerten , I. E. Demir , K. H. Schäfer , Nat. Rev. Gastroenterol. Hepatol. 2021, 18, 393. 33514916 10.1038/s41575-020-00385-2

[34] M. Rao , M. D. Gershon , Nat. Rev. Gastroenterol. Hepatol. 2016, 13, 517. 27435372 10.1038/nrgastro.2016.107 PMC5005185

[35] X. Lv , F. Gao , X. Cao , Cell. Metab. 2022, 34, 1914. 36257317 10.1016/j.cmet.2022.09.025 PMC9742337

[36] Q. Q. Wan , W. P. Qin , Y. X. Ma , M. J. Shen , J. Li , Z. B. Zhang , J. H. Chen , F. R. Tay , L. N. Niu , K. Jiao , Adv. Sci. 2021, 8, 2003390. 10.1002/advs.202003390 PMC8025013 33854888

[37] a) S. Huang , Z. Li , Y. Liu , D. Gao , X. Zhang , J. Hao , F. Yang , J. Cell. Physiol. 2019, 234, 5466; 29377116 10.1002/jcp.26502

[38] Z. Zhang , Z. Hao , C. Xian , Y. Fang , B. Cheng , J. Wu , J. Xia , Acta Biomater. 2022, 153, 1. 36116724 10.1016/j.actbio.2022.09.023

[39] I. Ibrahim , S. Syamala , J. A. Ayariga , J. Xu , B. K. Robertson , S. Meenakshisundaram , O. S. Ajayi , Metabolites 2022, 12, 1247. 36557285 10.3390/metabo12121247 PMC9781427

[40] R. E. Tomlinson , B. A. Christiansen , A. A. Giannone , D. C. Genetos , Front. Endocrinol. 2020, 11, 646. 10.3389/fendo.2020.00646 PMC7538664 33071963

[41] O. Delbono , A. C. Z. Rodrigues , H. J. Bonilla , M. L. Messi , Ageing Res. Rev. 2021, 67, 101305. 33610815 10.1016/j.arr.2021.101305 PMC8049122

[42] L. Micheli , L. Bertini , A. Bonato , N. Villanova , C. Caruso , M. Caruso , R. Bernini , F. Tirone , Nutrients 2023, 15, 1767. 37049607 10.3390/nu15071767 PMC10096778

[43] Q. Sun , L. Cheng , X. Zeng , X. Zhang , Z. Wu , P. Weng , Int. J. Biol. Macromol. 2020, 164, 1484. 32735929 10.1016/j.ijbiomac.2020.07.208

[44] S. Zundler , C. Günther , A. E. Kremer , M. M. Zaiss , V. Rothhammer , M. F. Neurath , Nat. Rev. Gastroenterol. Hepatol. 2023, 20, 50. 35945456 10.1038/s41575-022-00663-1

[45] X. Huang , B. Hussain , J. Chang , CNS Neurosci. Ther. 2021, 27, 36. 33381913 10.1111/cns.13569 PMC7804893

[46] J. Wang , N. Zhu , X. Su , Y. Gao , R. Yang , Cells 2023, 12, 793. 36899929 10.3390/cells12050793 PMC10000530

[47] X. Zhu , B. Li , P. Lou , T. Dai , Y. Chen , A. Zhuge , Y. Yuan , L. Li , Neurosci. Bull. 2021, 37, 1510. 34216356 10.1007/s12264-021-00730-8 PMC8490573

[48] A. Zhou , Y. Yuan , M. Yang , Y. Huang , X. Li , S. Li , S. Yang , B. Tang , Front. Cell Infect. Microbiol. 2022, 12, 832672. 35155283 10.3389/fcimb.2022.832672 PMC8829037

[49] B. Das , G. B. Nair , J. Biosci. 2019, 44, 1. 31719226

[50] a) H. Stolp , K. Dziegielewska , Neuropathol Appl. Neurobiol 2009, 35, 132; 19077110 10.1111/j.1365-2990.2008.01005.x

[51] L. Liu , J. R. Huh , K. Shah , EBioMedicine 2022, 77, 103908. 35255456 10.1016/j.ebiom.2022.103908 PMC8897630

[52] J. Aguilera‐Lizarraga , H. Hussein , G. E. Boeckxstaens , Nat. Rev. Immunol. 2022, 22, 1. 35322260 10.1038/s41577-022-00713-4

[53] D. Yang , N. Almanzar , I. M. Chiu , Cell. Mol. Immunol. 2023, 20, 1. 37336989 10.1038/s41423-023-01054-5 PMC10616093

[54] S. Luo , Z. Chen , L. Deng , Y. Chen , W. Zhou , F. Canavese , L. Li , Nutrients 2023, 15, 3934. 37764718 10.3390/nu15183934 PMC10534888

[55] H. Y. Dar , S. Pal , P. Shukla , P. K. Mishra , G. B. Tomar , N. Chattopadhyay , R. K. Srivastava , Nutrition 2018, 54, 118. 29793054 10.1016/j.nut.2018.02.013

[56] A. M. Tyagi , Endocrinol., Diabetes Metab. 2024, 7, 440. 10.1002/edm2.440 PMC10782069 37505196

[57] a) G. Duque , D. C. Huang , N. Dion , M. Macoritto , D. Rivas , W. Li , X. F. Yang , J. Li , J. Lian , F. T. Marino , J. Bone Miner. Res. 2011, 26, 1472; 21308779 10.1002/jbmr.350

[58] H. Y. Dar , P. Shukla , P. K. Mishra , R. Anupam , R. K. Mondal , G. B. Tomar , V. Sharma , R. K. Srivastava , Bone Rep. 2018, 8, 46. 29955622 10.1016/j.bonr.2018.02.001 PMC6019967

[59] K. Atarashi , T. Tanoue , K. Oshima , W. Suda , Y. Nagano , H. Nishikawa , S. Fukuda , T. Saito , S. Narushima , K. Hase , Nature 2013, 500, 232. 23842501 10.1038/nature12331

[60] a) J. Y. Li , M. Yu , A. M. Tyagi , C. Vaccaro , E. Hsu , J. Adams , T. Bellido , M. N. Weitzmann , R. Pacifici , J. Bone Miner. Res. 2019, 34, 349; 30399207 10.1002/jbmr.3600

[61] H. Cheng , X. Guan , D. Chen , W. Ma , Microorganisms 2019, 7, 583. 31756956 10.3390/microorganisms7120583 PMC6956175

[62] K. M. Cawley , N. C. Bustamante‐Gomez , A. G. Guha , R. S. MacLeod , J. Xiong , I. Gubrij , Y. Liu , R. Mulkey , M. Palmieri , J. D. Thostenson , Cell Rep. 2020, 32. 10.1016/j.celrep.2020.108052 PMC7493998 32905775

[63] M. N. Weitzmann , I. Ofotokun , Nat. Rev. Endocrinol. 2016, 12, 518. 27312863 10.1038/nrendo.2016.91 PMC5857945

[64] a) N. Deshet‐Unger , A. Kolomansky , N. Ben‐Califa , S. Hiram‐Bab , D. Gilboa , T. Liron , M. Ibrahim , Z. Awida , A. Gorodov , H. S. Oster , Theranostics 2020, 10, 8744; 32754275 10.7150/thno.45845 PMC7392011

[65] X. Tong , J. Gu , R. Song , D. Wang , Z. Sun , C. Sui , C. Zhang , X. Liu , J. Bian , Z. Liu , J. Cell. Biochem. 2019, 120, 1630. 30256440 10.1002/jcb.27468

[66] K. D. Seely , C. A. Kotelko , H. Douglas , B. Bealer , A. E. Brooks , Int. J. Mol. Sci. 2021, 22, 9452. 34502371 10.3390/ijms22179452 PMC8431678

[67] Y. Pang , S. Zhu , J. Xu , C. Su , B. Wu , C. Zhang , J. Gao , Adv. Biol. 2023, 7, 2200321. 10.1002/adbi.202200321 36750967

[68] S. Thakur , R. Dhapola , P. Sarma , B. Medhi , D. H. Reddy , Inflammation 2023, 46, 1. 35986874 10.1007/s10753-022-01721-1

[69] K. Yokota , Immunol. Med. 2024, 47, 1. 37309864 10.1080/25785826.2023.2220931

[70] a) V. Fischer , M. Haffner‐Luntzer , Semin. Cell Dev. Biol. 2022, 123, 14; 34024716 10.1016/j.semcdb.2021.05.014

[71] Z. I. Kolabas , L. B. Kuemmerle , R. Perneczky , B. Förstera , S. Ulukaya , M. Ali , S. Kapoor , L. M. Bartos , M. Büttner , O. S. Caliskan , Cell 2023, 186, 3706. 37562402 10.1016/j.cell.2023.07.009 PMC10443631

[72] B. Cui , F. Peng , J. Lu , B. He , Q. Su , H. Luo , Z. Deng , T. Jiang , K. Su , Y. Huang , Brain Behav. Immun. 2021, 93, 368. 33160090 10.1016/j.bbi.2020.11.005

[73] Y. Baumer , M. A. Pita , A. S. Baez , L. R. Ortiz‐Whittingham , M. A. Cintron , R. R. Rose , V. C. Gray , F. Osei Baah , T. M. Powell‐Wiley , Clin. Sci. 2023, 137, 469. 10.1042/CS20220304 PMC10039705 36960908

[74] a) A. A. A. Reyes , D. J. Chandler , Neuroglia Aging Brain 2023, 4, 87;

[75] M. H. Ahmad , M. A. Rizvi , M. Ali , A. C. Mondal , Ageing Res. Rev. 2023, 85, 101840. 36603690 10.1016/j.arr.2022.101840

[76] R. Huo , B. Zeng , L. Zeng , K. Cheng , B. Li , Y. Luo , H. Wang , C. Zhou , L. Fang , W. Li , Front. Cell Infect. Microbiol. 2017, 7, 489. 29250490 10.3389/fcimb.2017.00489 PMC5715198

[77] Y. Wang , L. H. Kasper , Brain Behav. Immun. 2014, 38, 1. 24370461 10.1016/j.bbi.2013.12.015 PMC4062078

[78] Y. Zhao , X. Peng , Q. Wang , Z. Zhang , L. Wang , Y. Xu , H. Yang , J. Bai , D. Geng , Endocr. Rev. 2024, 45, 95. 37459436 10.1210/endrev/bnad025

[79] V. K. Yadav , S. Balaji , P. S. Suresh , X. S. Liu , X. Lu , Z. Li , X. E. Guo , J. J. Mann , A. K. Balapure , M. D. Gershon , Nat. Med. 2010, 16, 308. 20139991 10.1038/nm.2098 PMC2836724

[80] K. G. Jameson , C. A. Olson , S. A. Kazmi , E. Y. Hsiao , Mol. Cell 2020, 78, 577. 32275853 10.1016/j.molcel.2020.03.006

[81] Y. Chen , J. Xu , Y. Chen , Nutrients 2021, 13, 2099.34205336

[82] J. M. Yano , K. Yu , G. P. Donaldson , G. G. Shastri , P. Ann , L. Ma , C. R. Nagler , R. F. Ismagilov , S. K. Mazmanian , E. Y. Hsiao , Cell 2015, 161, 264. 25860609 10.1016/j.cell.2015.02.047 PMC4393509

[83] M. M. Kaelberer , L. E. Rupprecht , W. W. Liu , P. Weng , D. V. Bohórquez , Annu. Rev. Neurosci. 2020, 43, 337. 32101483 10.1146/annurev-neuro-091619-022657 PMC7573801

[84] a) P. Strandwitz , K. H. Kim , D. Terekhova , J. K. Liu , A. Sharma , J. Levering , D. McDonald , D. Dietrich , T. R. Ramadhar , A. Lekbua , Nat. Microbiol. 2019, 4, 396; 30531975 10.1038/s41564-018-0307-3 PMC6384127

[85] K. F. Azman , R. Zakaria , Int. J. Mol. Sci. 2022, 23, 6827.35743271

[86] E. P. Moreno‐Jiménez , M. Flor‐García , J. Terreros‐Roncal , A. Rábano , F. Cafini , N. Pallas‐Bazarra , J. Ávila , M. Llorens‐Martín , Nat. Med. 2019, 25, 554. 30911133 10.1038/s41591-019-0375-9

[87] A. Giacco , F. Cioffi , A. Cuomo , R. Simiele , R. Senese , E. Silvestri , A. Amoresano , C. Fontanarosa , G. Petito , M. Moreno , Nutrients 2022, 14, 1166. 35334826 10.3390/nu14061166 PMC8952016

[88] S. Zierold , K. Buschmann , S. Gachkar , M. L. Bochenek , D. Velmeden , L. Hobohm , C. F. Vahl , K. Schäfer , J. Am. Heart Assoc. 2021, 10, 018322. 10.1161/JAHA.120.018322 PMC8174206 33666096

[89] N. Zemanova , R. Omelka , V. Mondockova , V. Kovacova , M. Martiniakova , Biology 2022, 11, 1402. 36290306 10.3390/biology11101402 PMC9598716

[90] M. A. Terkawi , G. Matsumae , T. Shimizu , D. Takahashi , K. Kadoya , N. Iwasaki , Int. J. Mol. Sci. 2022, 23, 1786. 35163708 10.3390/ijms23031786 PMC8836472

[91] H. Maagensen , M. M. Helsted , L. S. Gasbjerg , T. Vilsbøll , F. K. Knop , Curr. Osteoporos Rep. 2023, 21, 21. 36441432 10.1007/s11914-022-00767-2

[92] J. Yan , J. W. Herzog , K. Tsang , C. A. Brennan , M. A. Bower , W. S. Garrett , B. R. Sartor , A. O. Aliprantis , J. F. Charles , Proc. Natl. Acad. Sci. USA 2016, 113, 7554. 10.1073/pnas.1607235113 PMC5127374 27821775

[93] A. Y. Sato , D. Richardson , M. Cregor , H. M. Davis , E. D. Au , K. McAndrews , T. A. Zimmers , J. M. Organ , M. Peacock , L. I. Plotkin , Endocrinology 2017, 158, 664. 28359087 10.1210/en.2016-1779 PMC5460781

[94] Y. Tu , R. Yang , X. Xu , X. Zhou , J. Leukoc. Biol. 2021, 110, 525. 33884666 10.1002/JLB.3MR0321-755R

[95] C. Willyard , Nature 2021, 590, 22. 33536656 10.1038/d41586-021-00260-3

[96] a) S. Zhu , Y. Jiang , K. Xu , M. Cui , W. Ye , G. Zhao , L. Jin , X. Chen , J. Neuroinflammation 2020, 17, 1; 31952509 10.1186/s12974-020-1705-z PMC6969442

[97] B. van der Hee , J. M. Wells , Trends Microbiol. 2021, 29, 700. 33674141 10.1016/j.tim.2021.02.001

[98] Y. Wu , Z. Hang , T. Lei , H. Du , Neurochem. Res. 2022, 47, 3565. 36309938 10.1007/s11064-022-03784-w

[99] Y. H. Kwon , H. Wang , E. Denou , J. E. Ghia , L. Rossi , M. E. Fontes , S. P. Bernier , M. S. Shajib , S. Banskota , S. M. Collins , Cell. Mol. Gastroenterol. Hepatol. 2019, 7, 709. 30716420 10.1016/j.jcmgh.2019.01.004 PMC6462823

[100] M. Sun , N. Ma , T. He , L. J. Johnston , X. Ma , Crit. Rev. Food Sci. Nutr. 2020, 60, 1760. 30924357 10.1080/10408398.2019.1598334

[101] N. Ma , T. He , L. J. Johnston , X. Ma , Gut. Microbes 2020, 11, 1203. 32401136 10.1080/19490976.2020.1758008 PMC7524279

[102] S. Shandilya , S. Kumar , N. K. Jha , K. K. Kesari , J. Ruokolainen , J. Adv. Res. 2022, 38, 223. 35572407 10.1016/j.jare.2021.09.005 PMC9091761

[103] L. W. Yu , G. Agirman , E. Y. Hsiao , Annu. Rev. Immunol. 2022, 40, 143. 34990209 10.1146/annurev-immunol-101320-014237

[104] A. Majumdar , I. P. Siva Venkatesh , A. Basu , ACS Chem. Neurosci. 2023, 14, 1045. 36868874 10.1021/acschemneuro.2c00803

[105] N. Singh , V. Singh , S. N. Rai , V. Mishra , E. Vamanu , M. P. Singh , Biomed. Pharmacother. 2022, 156, 113958. 36411639 10.1016/j.biopha.2022.113958

[106] J. Behera , J. Ison , S. C. Tyagi , N. Tyagi , Bone 2020, 135, 115317. 32169602 10.1016/j.bone.2020.115317 PMC8457311

[107] J. J. Ni , X. L. Yang , H. Zhang , Q. Xu , X. T. Wei , G. J. Feng , M. Zhao , Y. F. Pei , L. Zhang , Bone 2021, 143, 115652. 32971307 10.1016/j.bone.2020.115652

[108] J. He , S. Xu , B. Zhang , C. Xiao , Z. Chen , F. Si , J. Fu , X. Lin , G. Zheng , G. Yu , Aging 2020, 12, 8583. 32392181 10.18632/aging.103168 PMC7244073

[109] a) N. Gasaly , P. De Vos , M. A. Hermoso , Front. Immunol. 2021, 12, 658354; 34122415 10.3389/fimmu.2021.658354 PMC8187770

[110] M. Cantley , D. Fairlie , P. Bartold , K. Rainsford , G. Le , A. Lucke , C. Holding , D. Haynes , J. Cell. Physiol. 2011, 226, 3233. 21344383 10.1002/jcp.22684

[111] C. Montalvany‐Antonucci , L. Duffles , J. de Arruda , M. Zicker , S. de Oliveira , S. Macari , G. P. Garlet , M. Madeira , S. Y. Fukada , I. Andrade Jr , Bone 2019, 125, 112. 31100533 10.1016/j.bone.2019.05.016

[112] K. F. Contino , H. Yadav , Y. Shiozawa , Biochem. Pharmacol. 2022, 197, 114916. 35041811 10.1016/j.bcp.2022.114916 PMC8858876

[113] J. M. Anaya , W. B. Bollag , M. W. Hamrick , C. M. Isales , Int. J. Mol. Sci. 2020, 21, 6670. 32933099 10.3390/ijms21186670 PMC7555967

[114] C. Vidal , W. Li , B. Santner‐Nanan , C. K. Lim , G. J. Guillemin , H. J. Ball , N. H. Hunt , R. Nanan , G. Duque , Stem Cells 2015, 33, 111. 25186311 10.1002/stem.1836

[115] B. Kalaska , K. Pawlak , T. Domaniewski , E. Oksztulska‐Kolanek , B. Znorko , A. Roszczenko , J. Rogalska , M. M. Brzoska , P. Lipowicz , M. Doroszko , Front. Physiol. 2017, 8, 836. 29163188 10.3389/fphys.2017.00836 PMC5671515

[116] C. Chevalier , S. Kieser , M. Çolakoğlu , N. Hadadi , J. Brun , D. Rigo , N. Suárez‐Zamorano , M. Spiljar , S. Fabbiano , B. Busse , Cell. Metab. 2020, 32, 575. 32916104 10.1016/j.cmet.2020.08.012 PMC7116155

[117] M. M. Zaiss , R. M. Jones , G. Schett , R. Pacifici , J. Clin. Invest. 2019, 129, 3018. 31305265 10.1172/JCI128521 PMC6668676

[118] E. Emekli‐Alturfan , A. A. Alturfan , Mol. Biol. Rep. 2023, 50, 815. 36329336 10.1007/s11033-022-07925-w

[119] F. L. da Silva , E. C. Cerqueira , M. S. de Freitas , D. L. Gonçalves , L. T. Costa , C. Follmer , Neurochem. Int. 2013, 62, 103. 23064431 10.1016/j.neuint.2012.10.001

[120] S. L. Booth , M. K. Shea , K. Barger , S. E. Leurgans , B. D. James , T. M. Holland , P. Agarwal , X. Fu , J. Wang , G. Matuszek , Alzheimers Dement (N Y). 2022, 8, 12255. 10.1002/trc2.12255 PMC9019903 35475263

[121] X. X. Liu , P. F. Wu , Y. Z. Liu , Y. L. Jiang , M. D. Wan , X. W. Xiao , Q. J. Yang , B. Jiao , X. X. Liao , J. L. Wang , J. Alzheimer's Dis. 2022, 85, 829. 34864672 10.3233/JAD-215104

[122] M. Fusaro , M. C. Mereu , A. Aghi , G. Iervasi , M. Gallieni , Clin. Cases Miner. Bone Metab. 2017, 14, 200. 29263734 10.11138/ccmbm/2017.14.1.200 PMC5726210

[123] N. Alonso , A. Meinitzer , E. Fritz‐Petrin , D. Enko , M. Herrmann , Calcif. Tissue Int. 2023, 112, 178. 35150288 10.1007/s00223-022-00955-3 PMC9859868

[124] L. Pereira , R. Monteiro , Clin. Nutr. ESPEN 2022, 51, 37. 36184230 10.1016/j.clnesp.2022.08.002

[125] J. Yuan , B. P. Meloni , T. Shi , A. Bonser , J. M. Papadimitriou , F. L. Mastaglia , C. Zhang , M. Zheng , J. Gao , J. Alzheimer's Dis. 2019, 69, 59. 30932886 10.3233/JAD-181249

[126] F. Angelucci , K. Cechova , J. Amlerova , J. Hort , J. Neuroinflammation 2019, 16, 1. 31118068 10.1186/s12974-019-1494-4 PMC6530014

[127] V. T. Aho , P. A. Pereira , S. Voutilainen , L. Paulin , E. Pekkonen , P. Auvinen , F. Scheperjans , EBioMedicine 2019, 44, 691. 31221587 10.1016/j.ebiom.2019.05.064 PMC6606744

[128] C. I. Wasser , E. C. Mercieca , G. Kong , A. J. Hannan , S. J. McKeown , Y. Glikmann‐Johnston , J. C. Stout , Brain Commun. 2020, 2, 110. 10.1093/braincomms/fcaa110 PMC7519724 33005892

[129] J. Ochoa‐Repáraz , T. O. Kirby , L. H. Kasper , Perspect. Med. 2018, 8, 029017. 10.1101/cshperspect.a029017 PMC5983160 29311123

[130] D. S. Knopman , H. Amieva , R. C. Petersen , G. Chételat , D. M. Holtzman , B. T. Hyman , R. A. Nixon , D. T. Jones , Nat. Rev. Dis. Primers 2021, 7, 1. 33986301 10.1038/s41572-021-00269-y PMC8574196

[131] D. A. Stakos , K. Stamatelopoulos , D. Bampatsias , M. Sachse , E. Zormpas , N. I. Vlachogiannis , S. Tual‐Chalot , K. Stellos , J. Am. Coll. Cardiol. 2020, 75, 952. 32130931 10.1016/j.jacc.2019.12.033 PMC7042886

[132] K. Ramakrishna , L. V. Nalla , D. Naresh , K. Venkateswarlu , M. K. Viswanadh , B. N. Nalluri , G. Chakravarthy , S. Duguluri , P. Singh , S. N. Rai , Diseases 2023, 11, 89. 37489441 10.3390/diseases11030089 PMC10366863

[133] C. M. Dengler‐Crish , H. C. Ball , L. Lin , K. M. Novak , L. N. Cooper , Neurobiol. Aging 2018, 67, 148. 29660685 10.1016/j.neurobiolaging.2018.03.021

[134] F. Pistollato , S. Sumalla Cano , I. Elio , M. Masias Vergara , F. Giampieri , M. Battino , Nutr. Rev. 2016, 74, 624. 27634977 10.1093/nutrit/nuw023

[135] L. Wu , X. Xian , G. Xu , Z. Tan , F. Dong , M. Zhang , F. Zhang , Mediators Inflammation 2022, 2022, 7924199. 10.1155/2022/7924199 PMC9420645 36046763

[136] B. Zou , J. Li , R. X. Ma , X. Y. Cheng , R.‐Y. Ma , T. Y. Zhou , Z. Q. Wu , Y. Yao , J. Li , Aging Dis. 2023, 14, 964. 37191418 10.14336/AD.2022.1127 PMC10187701

[137] S. Chandra , S. S. Sisodia , R. J. Vassar , Mol. Neurodegener. 2023, 18, 1. 36721148 10.1186/s13024-023-00595-7 PMC9889249

[138] M. Dhami , K. Raj , S. Singh , Aging Health Res. 2023, 3, 100128.

[139] M. S. Kim , Y. Kim , H. Choi , W. Kim , S. Park , D. Lee , D. K. Kim , H. J. Kim , H. Choi , D. W. Hyun , Gut 2020, 69, 283. 31471351 10.1136/gutjnl-2018-317431

[140] S. Liu , J. Gao , M. Zhu , K. Liu , H. L. Zhang , Mol. Neurobiol. 2020, 57, 5026. 32829453 10.1007/s12035-020-02073-3 PMC7541367

[141] Y. H. Chen , R. Y. Lo , Tzu. Chi. Med. J. 2017, 29, 138. 10.4103/tcmj.tcmj_54_17 PMC5615992 28974906

[142] N. Loskutova , R. A. Honea , W. M. Brooks , J. M. Burns , J. Alzheimer's Dis. 2010, 20, 313. 20164583 10.3233/JAD-2010-1364 PMC2892930

[143] S. G. Jeon , M. Y. Cha , J. I. Kim , T. W. Hwang , K. A. Kim , T. H. Kim , K. C. Song , J. J. Kim , M. Moon , Nanomedicine 2019, 17, 297. 30794963 10.1016/j.nano.2019.02.004

[144] H. Depypere , A. Vierin , S. Weyers , A. Sieben , Maturitas 2016, 94, 98. 27823753 10.1016/j.maturitas.2016.09.009

[145] M. C. Jurcău , F. L. Andronie‐Cioara , A. Jurcău , F. Marcu , D. M. Ţiț , N. Pașcalău , D. C. Nistor‐Cseppentö , Antioxidants 2022, 11, 2167. 36358538 10.3390/antiox11112167 PMC9686795

[146] A. Guerrero , B. De Strooper , I. L. Arancibia‐Cárcamo , Trends Neurosci. 2021, 44, 714. 34366147 10.1016/j.tins.2021.06.007

[147] D. Goyal , S. A. Ali , R. K. Singh , Neuropsychopharmacol. Biol. Psychiatry 2021, 106, 110112. 10.1016/j.pnpbp.2020.110112 32949638

[148] K. Fehsel , J. Christl , Ageing Res. Rev. 2022, 76, 101592. 35192961 10.1016/j.arr.2022.101592

[149] R. Balestrino , A. Schapira , Eur. J. Neurol 2020, 27, 27. 31631455 10.1111/ene.14108

[150] W. Poewe , K. Seppi , C. M. Tanner , G. M. Halliday , P. Brundin , J. Volkmann , A. E. Schrag , A. E. Lang , Nat. Rev. Dis. Primers 2017, 3, 1. 10.1038/nrdp.2017.13 28332488

[151] S. Kim , S. H. Kwon , T. I. Kam , N. Panicker , S. S. Karuppagounder , S. Lee , J. H. Lee , W. R. Kim , M. Kook , C. A. Foss , Neuron 2019, 103, 627. 31255487 10.1016/j.neuron.2019.05.035 PMC6706297

[152] A. Serafino , M. Cozzolino , Neural Regener. Res. 2023, 18, 306. 10.4103/1673-5374.343908 PMC9396482 35900408

[153] S. K. Yadav , S. N. Rai , S. P. Singh , J. Chem. Neuroanat 2017, 80, 1. 27919828 10.1016/j.jchemneu.2016.11.009

[154] J. Prakash , S. Chouhan , S. K. Yadav , S. Westfall , S. N. Rai , S. P. Singh , Neurochem. Res. 2014, 39, 2527. 25403619 10.1007/s11064-014-1443-7

[155] I. F. Ivan , V. L. Irincu , Ş. Diaconu , O. Falup‑Pecurariu , B. Ciopleiaș , C. Falup‑Pecurariu , Exp. Ther Med. 2021, 22, 1. 10.3892/etm.2021.10517 PMC8355716 34447476

[156] M. Lubomski , A. H. Tan , S. Y. Lim , A. J. Holmes , R. L. Davis , C. M. Sue , J. Neurol. 2020, 267, 2507. 31041582 10.1007/s00415-019-09320-1

[157] M. Barichella , M. Severgnini , R. Cilia , E. Cassani , C. Bolliri , S. Caronni , V. Ferri , R. Cancello , C. Ceccarani , S. Faierman , Mov. Disord. 2019, 34, 396. 30576008 10.1002/mds.27581

[158] S. Romano , G. M. Savva , J. R. Bedarf , I. G. Charles , F. Hildebrand , A. Narbad , Parkinson's Dis. 2021, 7, 27. 10.1038/s41531-021-00156-z PMC7946946 33692356

[159] S. Li , L. Zhao , J. Xiao , Y. Guo , R. Fu , Y. Zhang , S. Xu , Mol. Cell. Biochem. 2023, 1. 10.1007/s11010-023-04853-6 37787835

[160] a) M. F. Sun , Y. L. Zhu , Z. L. Zhou , X. B. Jia , Y. D. Xu , Q. Yang , C. Cui , Y. Q. Shen , Brain Behav. Immun. 2018, 70, 48; 29471030 10.1016/j.bbi.2018.02.005

[161] Z. L. Zhou , X. B. Jia , M. F. Sun , Y. L. Zhu , C. M. Qiao , B. P. Zhang , L. P. Zhao , Q. Yang , C. Cui , X. Chen , Neurotherapeutics 2019, 16, 741. 30815845 10.1007/s13311-019-00719-2 PMC6694382

[162] X. Y. Kuai , X. H. Yao , L. J. Xu , Y. Q. Zhou , L. P. Zhang , Y. Liu , S. F. Pei , C. L. Zhou , Microb. Cell Fact 2021, 20, 1. 33985520 10.1186/s12934-021-01589-0 PMC8120701

[163] P. Gazerani , Int. J. Mol. Sci. 2019, 20, 4121.31450864

[164] G. Bivona , B. L. Sasso , G. Iacolino , C. M. Gambino , C. Scazzone , L. Agnello , M. Ciaccio , Clin. Chim. Acta 2019, 497, 82. 31330127 10.1016/j.cca.2019.07.022

[165] S. Malochet‐Guinamand , F. Durif , T. Thomas , Jt., Bone, Spine 2015, 82, 406. 10.1016/j.jbspin.2015.03.009 26453100

[166] C. A. Figueroa , C. J. Rosen , Expert Rev. Endocrinol. Metab. 2020, 15, 185. 32336178 10.1080/17446651.2020.1756772 PMC7250483

[167] Z. Lyu , Y. Hu , Y. Guo , D. Liu , Bone Res. 2023, 11, 31. 37296111 10.1038/s41413-023-00264-x PMC10256815

[168] B. R. Leavitt , H. B. Kordasiewicz , S. Schobel , JAMA Neurol. 2020, 77, 764. 32202594 10.1001/jamaneurol.2020.0299

[169] G. P. Bates , R. Dorsey , J. F. Gusella , M. R. Hayden , C. Kay , B. R. Leavitt , M. Nance , C. A. Ross , R. I. Scahill , R. Wetzel , Nat. Rev. Dis. Primers 2015, 1, 1. 10.1038/nrdp.2015.5 27188817

[170] C. Smith‐Geater , S. J. Hernandez , R. G. Lim , M. Adam , J. Wu , J. T. Stocksdale , B. T. Wassie , M. P. Gold , K. Q. Wang , R. Miramontes , Stem Cell Rep. 2020, 14, 406. 10.1016/j.stemcr.2020.01.015 PMC7066322 32109367

[171] S. J. Tabrizi , R. Ghosh , B. R. Leavitt , Neuron 2019, 101, 801. 30844400 10.1016/j.neuron.2019.01.039

[172] L. Przybyl , M. Wozna‐Wysocka , E. Kozlowska , A. Fiszer , Int. J. Mol. Sci. 2021, 22, 1561. 33557131 10.3390/ijms22041561 PMC7913877

[173] G. Kong , K. A. Lê Cao , L. M. Judd , S. Li , T. Renoir , A. J. Hannan , Neurobiol. Dis. 2020, 135, 104268. 30194046 10.1016/j.nbd.2018.09.001

[174] G. Kong , S. Ellul , V. K. Narayana , K. Kanojia , H. T. T. Ha , S. Li , T. Renoir , K. A. Le Cao , A. J. Hannan , Neurobiol. Dis. 2021, 148, 105199. 33249136 10.1016/j.nbd.2020.105199

[175] C. I. Radulescu , M. Garcia‐Miralles , H. Sidik , C. F. Bardile , N. A. B. M. Yusof , H. U. Lee , E. X. P. Ho , C. W. Chu , E. Layton , D. Low , Neurobiol. Dis. 2019, 127, 65. 30802499 10.1016/j.nbd.2019.02.011

[176] a) J. M. van der Burg , A. Winqvist , N. A. Aziz , M. L. Maat‐Schieman , R. A. Roos , G. P. Bates , P. Brundin , M. Björkqvist , N. Wierup , Neurobiol. Dis. 2011, 44, 1; 21624468 10.1016/j.nbd.2011.05.006

[177] E. Cubo , J. Rivadeneyra , C. Gil‐Polo , D. Armesto , A. Mateos , N. Mariscal‐Pérez , J. Neurol Sci. 2015, 358, 335. 26394908 10.1016/j.jns.2015.09.351

[178] R. R. Ribchester , D. Thomson , N. I. Wood , T. Hinks , T. H. Gillingwater , T. M. Wishart , F. A. Court , A. J. Morton , Eur. J. Neurosci. 2004, 20, 3092. 15579164 10.1111/j.1460-9568.2004.03783.x

[179] M. Turner , A. Reyes , D. M. Bartlett , S. Culpin , N. H. Hart , L. Hardt , K. Feindel , G. R. Poudel , M. Ziman , T. M. Cruickshank , J. Musculoskelet. Neuronal. Interact. 2020, 20, 332. 32877970 PMC7493441

[180] R. C. de Miranda , N. Di Lorenzo , A. Andreoli , L. Romano , G. L. De Santis , P. Gualtieri , A. De Lorenzo , Nutrition 2019, 59, 145. 30468934 10.1016/j.nut.2018.08.005

[181] M. C. Kiernan , S. Vucic , B. C. Cheah , M. R. Turner , A. Eisen , O. Hardiman , J. R. Burrell , M. C. Zoing , Lancet 2011, 377, 942. 21296405 10.1016/S0140-6736(10)61156-7

[182] R. J. Mead , N. Shan , H. J. Reiser , F. Marshall , P. J. Shaw , Nat. Rev. Drug Discovery 2023, 22, 185. 36543887 10.1038/s41573-022-00612-2 PMC9768794

[183] a) K. E. Meijboom , R. H. Brown , Neurotherapeutics 2022, 19, 1159; 36068427 10.1007/s13311-022-01285-w PMC9587165

[184] C. Wong , M. Stavrou , E. Elliott , J. M. Gregory , N. Leigh , A. Pinto , T. Williams , J. Chataway , R. Swingler , M. K. B. Parmar , N. Stallard , C. J. Weir , R. A. Parker , A. Chaouch , H. Hamdalla , J. Ealing , G. Gorrie , I. Morrison , C. Duncan , P. J. Connelly , F. J. Carod‐Artal , R. Davenport , P. G. Reitboeck , A. Radunovic , V. Srinivasan , J. Preston , A. R. Mehta , D. Leighton , S. A. Glasmacher , E. Beswick , et al., Brain Commun. 2021, 3, 242. 10.1093/braincomms/fcab242 PMC8659356 34901853

[185] A. Breiner , L. Zinman , P. R. Bourque , Can. Med. Assoc. J. 2020, 192, 319. 10.1503/cmaj.191236 PMC7101182 32392516

[186] X. Jiang , Y. Guan , Z. Zhao , F. Meng , X. Wang , X. Gao , J. Liu , Y. Chen , F. Zhou , S. Zhou , Cells 2021, 10, 839. 33917816 10.3390/cells10040839 PMC8068170

[187] D. Di Gioia , N. Bozzi Cionci , L. Baffoni , A. Amoruso , M. Pane , L. Mogna , F. Gaggìa , M. A. Lucenti , E. Bersano , R. Cantello , BMC Med. 2020, 18, 1. 32546239 10.1186/s12916-020-01607-9 PMC7298784

[188] K. Nicholson , K. Bjornevik , G. Abu‐Ali , J. Chan , M. Cortese , B. Dedi , M. Jeon , R. Xavier , C. Huttenhower , A. Ascherio , Amyotrophic Lateral Scler. Frontotemporal Degener. 2021, 22, 186. 10.1080/21678421.2020.1828475 33135936

[189] E. Blacher , S. Bashiardes , H. Shapiro , D. Rothschild , U. Mor , M. Dori‐Bachash , C. Kleimeyer , C. Moresi , Y. Harnik , M. Zur , Nature 2019, 572, 474. 31330533 10.1038/s41586-019-1443-5

[190] R. R. Kelly , S. J. Sidles , A. C. LaRue , Front. Psychol. 2020, 11, 612366. 33424724 10.3389/fpsyg.2020.612366 PMC7793932

[191] a) J. Caplliure‐Llopis , D. Escrivá , M. Benlloch , J. E. de la Rubia Ortí , J. M. Estrela , C. Barrios , Front. Neurol. 2020, 11, 599216; 33391162 10.3389/fneur.2020.599216 PMC7775537

[192] a) C. Quessada , A. Bouscary , F. René , C. Valle , A. Ferri , S. T. Ngo , J. P. Loeffler , Cells 2021, 10, 1449; 34207859 10.3390/cells10061449 PMC8226541

[193] D. Sandi , Z. Kokas , T. Biernacki , K. Bencsik , P. Klivényi , L. Vécsei , Int. J. Mol. Sci. 2022, 23, 5162. 35563559 10.3390/ijms23095162 PMC9100097

[194] P. A. Calabresi , Am. Fam. Physician 2004, 70, 1935. 15571060

[195] H. Lassmann , W. Brück , C. F. Lucchinetti , Brain Pathol. 2007, 17, 210. 17388952 10.1111/j.1750-3639.2007.00064.x PMC8095582

[196] J. E. Lengfeld , S. E. Lutz , J. R. Smith , C. Diaconu , C. Scott , S. B. Kofman , C. Choi , C. M. Walsh , C. S. Raine , I. Agalliu , Proc. Natl. Acad. Sci. USA 2017, 114, 1168. 10.1073/pnas.1609905114 PMC5320985 28137846

[197] S. Jangi , R. Gandhi , L. M. Cox , N. Li , F. Von Glehn , R. Yan , B. Patel , M. A. Mazzola , S. Liu , B. L. Glanz , Nat. Commun. 2016, 7, 12015. 27352007 10.1038/ncomms12015 PMC4931233

[198] Y. Fan , J. Zhang , Front. Microbiol. 2019, 10, 740. 31040833 10.3389/fmicb.2019.00740 PMC6476896

[199] M. Kozhieva , N. Naumova , T. Alikina , A. Boyko , V. Vlassov , M. R. Kabilov , BMC Microbiol. 2019, 19, 1. 31888483 10.1186/s12866-019-1685-2 PMC6937728

[200] a) D. Takewaki , W. Suda , W. Sato , L. Takayasu , N. Kumar , K. Kimura , N. Kaga , T. Mizuno , S. Miyake , M. Hattori , Proc. Natl. Acad. Sci. USA 2020, 117, 22402; 32839304 10.1073/pnas.2011703117 PMC7486801

[201] S. Makkawi , C. Camara‐Lemarroy , L. Metz , Neurol. Neuroimmunol. Neuroinflamm. 2018, 5, 459. 10.1212/NXI.0000000000000459 PMC5882466 29619403

[202] R. Redondo‐Castillejo , A. Garcimartín , M. Hernández‐Martín , M. E. López‐Oliva , A. Bocanegra , A. Macho‐González , S. Bastida , J. Benedí , F. J. Sánchez‐Muniz , Int. J. Mol. Sci. 2023, 24, 5369. 36982444 10.3390/ijms24065369 PMC10049473

[203] M. T. Bazelier , T. van Staa , B. M. Uitdehaag , C. Cooper , H. G. Leufkens , P. Vestergaard , J. Bentzen , F. de Vries , J. Bone Miner. Res. 2011, 26, 2271. 21557309 10.1002/jbmr.418 PMC3193376

[204] B. J. Ross , A. J. Ross , O. C. Lee , T. L. Waters , M. M. Familia , W. F. Sherman , Osteoporos. Int. 2022, 33, 1999. 35670832 10.1007/s00198-022-06451-6

[205] C. S. Simonsen , E. G. Celius , C. Brunborg , C. Tallaksen , E. F. Eriksen , T. Holmøy , S. M. Moen , BMC Neurol. 2016, 16, 1. 27919248 10.1186/s12883-016-0771-4 PMC5139093

[206] A. P. Hearn , E. Silber , Mult. Scler. J. 2010, 16, 1031. 10.1177/1352458510368985 20551086

[207] I. Figueroa‐González , A. Cruz‐Guerrero , G. Quijano , J. Microbial. Biochem. Technol. 2011, 1, 1948.

[208] A. Peredo‐Lovillo , H. Romero‐Luna , M. Jiménez‐Fernández , Food Res. Int. 2020, 136, 109473. 32846558 10.1016/j.foodres.2020.109473

[209] C. Bedu‐Ferrari , P. Biscarrat , P. Langella , C. Cherbuy , Nutrients 2022, 14, 2096. 35631237 10.3390/nu14102096 PMC9147914

[210] M. I. Hossain , M. Sadekuzzaman , S. D. Ha , Food Res. Int. 2017, 100, 63. 28873730 10.1016/j.foodres.2017.07.077

[211] E. Vamanu , S. N. Rai , Diseases 2021, 9, 45. 34201465 10.3390/diseases9030045 PMC8293145

[212] D. A. Hall , R. M. Voigt , T. M. Cantu‐Jungles , B. Hamaker , P. A. Engen , M. Shaikh , S. Raeisi , S. J. Green , A. Naqib , C. B. Forsyth , Nat. Commun. 2023, 14, 926. 36801916 10.1038/s41467-023-36497-x PMC9938693

[213] T. H. Hsieh , C. W. Kuo , K. H. Hsieh , M. J. Shieh , C. W. Peng , Y. C. Chen , Y. L. Chang , Y. Z. Huang , C. C. Chen , P. K. Chang , Brain Sci. 2020, 10, 206. 32244769 10.3390/brainsci10040206 PMC7226147

[214] T. Li , C. Chu , L. Yu , Q. Zhai , S. Wang , J. Zhao , H. Zhang , W. Chen , F. Tian , Nutrients 2022, 14, 4678. 36364939 10.3390/nu14214678 PMC9655354

[215] H. Sun , F. Zhao , Y. Liu , T. Ma , H. Jin , K. Quan , B. Leng , J. Zhao , X. Yuan , Z. Li , Parkinson's Dis. 2022, 8, 62. 10.1038/s41531-022-00327-6 PMC9130297 35610236

[216] Y. Wang , D. Wang , H. Lv , Q. Dong , J. Li , W. Geng , J. Wang , F. Liu , L. Jia , Y. Wang , Mol. Nutr. Food Res. 2022, 66, 2200265. 10.1002/mnfr.202200265 35975737

[217] X. Song , Z. Zhao , Y. Zhao , Z. Wang , C. Wang , G. Yang , S. Li , Nutr. Neurosci. 2022, 25, 2588. 34755592 10.1080/1028415X.2021.1991556

[218] M. Nimgampalle , Y. Kuna , J. Clin. Diagn. Res. 2017, 11, 1. 10.7860/JCDR/2017/26106.10428 PMC5620801 28969160

[219] F. Pistollato , R. C. Iglesias , R. Ruiz , S. Aparicio , J. Crespo , L. D. Lopez , P. P. Manna , F. Giampieri , M. Battino , Pharmacol. Res. 2018, 131, 32. 29555333 10.1016/j.phrs.2018.03.012

[220] a) Q. He , J. Huang , T. Zheng , D. Lin , H. Zhang , J. Li , Z. Sun , FEMS Microbiol. Ecol. 2021, 97, 151; 10.1093/femsec/fiab151 34792102

[221] V. V. Giau , S. Y. Wu , A. Jamerlan , S. S. A. An , S. Kim , J. Hulme , Nutrients 2018, 10, 1765. 30441866 10.3390/nu10111765 PMC6266223

[222] A. Labarre , E. Guitard , G. Tossing , A. Forest , E. Bareke , M. Labrecque , M. Tétreault , M. Ruiz , J. Alex Parker , Commun. Biol. 2022, 5, 1340. 36477191 10.1038/s42003-022-04295-8 PMC9729297

[223] G. Sharma , S. S. Biswas , J. Mishra , U. Navik , R. Kandimalla , P. H. Reddy , G. K. Bhatti , J. S. Bhatti , Life Sci. 2023, 328, 121882. 37356750 10.1016/j.lfs.2023.121882

[224] S. K. Tankou , K. Regev , B. C. Healy , E. Tjon , L. Laghi , L. M. Cox , P. Kivisäkk , I. V. Pierre , L. Hrishikesh , R. Gandhi , Ann. Neurol. 2018, 83, 1147. 29679417 10.1002/ana.25244 PMC6181139

[225] J. Jiang , C. Chu , C. Wu , C. Wang , C. Zhang , T. Li , Q. Zhai , L. Yu , F. Tian , W. Chen , Food Funct. 2021, 12, 2354. 33629669 10.1039/d0fo03203d

[226] M. E. Amini , N. Shomali , A. Bakhshi , S. Rezaei , M. Hemmatzadeh , R. Hosseinzadeh , S. Eslami , F. Babaie , S. Aslani , S. Torkamandi , Int. Immunopharmacol. 2020, 88, 107024. 33182024 10.1016/j.intimp.2020.107024

[227] J. Correale , R. Hohlfeld , S. E. Baranzini , Nat. Rev. Neurol. 2022, 18, 544. 35931825 10.1038/s41582-022-00697-8

[228] F. L. Collins , N. D. Rios‐Arce , J. D. Schepper , N. Parameswaran , L. R. McCabe , Microbiol. Spectr. 2017, 5, 5.4.20. 10.1128/microbiolspec.bad-0015-2016 PMC5710820 28840819

[229] P. Li , B. Ji , H. Luo , D. Sundh , M. Lorentzon , J. Nielsen , npj Biofilms Microbiomes 2022, 8, 84. 36261538 10.1038/s41522-022-00348-2 PMC9581899

[230] J. Yu , G. Cao , S. Yuan , C. Luo , J. Yu , M. Cai , BMJ Open 2021, 11, 041393. 10.1136/bmjopen-2020-041393 PMC7929795 33653743

[231] A. de Sire , R. de Sire , C. Curci , F. Castiglione , W. Wahli , Cells 2022, 11, 743. 35203401 10.3390/cells11040743 PMC8870226

[232] K. O. Kim , M. Gluck , Clin. Endosc. 2019, 52, 137. 30909689 10.5946/ce.2019.009 PMC6453848

[233] R. Chen , Y. Xu , P. Wu , H. Zhou , Y. Lasanajak , Y. Fang , L. Tang , L. Ye , X. Li , Z. Cai , Pharmacol. Res. 2019, 148, 104403. 31425750 10.1016/j.phrs.2019.104403

[234] K. Li , S. Wei , L. Hu , X. Yin , Y. Mai , C. Jiang , X. Peng , X. Cao , Z. Huang , H. Zhou , Mediators Inflammation 2020, 2020, 2058272. 10.1155/2020/2058272 PMC7426773 32831634

[235] J. Mandrioli , A. Amedei , G. Cammarota , E. Niccolai , E. Zucchi , R. D'Amico , F. Ricci , G. Quaranta , T. Spanu , L. Masucci , Front. Neurol. 2019, 10, 1021. 31620079 10.3389/fneur.2019.01021 PMC6763586

[236] E. Bok , M. Jo , S. Lee , B. R. Lee , J. Kim , H. J. Kim , Int. J. Mol. Sci. 2019, 20, 464. 30678217 10.3390/ijms20030464 PMC6386998

[237] J. A. Luchsinger , M. X. Tang , S. Shea , R. Mayeux , Arch. Neurol. 2002, 59, 1258. 12164721 10.1001/archneur.59.8.1258

[238] M. H. Baky , M. Salah , N. Ezzelarab , P. Shao , M. S. Elshahed , M. A. Farag , Crit. Rev. Food Sci. Nutr. 2024, 1954. 36094440 10.1080/10408398.2022.2119931

[239] D. Parada Venegas , M. K. De la Fuente , G. Landskron , M. J. González , R. Quera , G. Dijkstra , H. J. Harmsen , K. N. Faber , M. A. Hermoso , Front. Immunol. 2019, 277. 30915065 10.3389/fimmu.2019.00277 PMC6421268

[240] A. E. Sanders , E. D. Weatherspoon , B. M. Ehrmann , P. S. Soma , S. R. Shaikh , J. S. Preisser , R. Ohrbach , R. B. Fillingim , G. D. Slade , J. Pain 2022, 23, 1737. 35477107 10.1016/j.jpain.2022.04.006 PMC9561958

[241] F. Pistollato , M. Battino , Trends Food Sci. Technol. 2014, 40, 62.

[242] A. Rivas , A. Romero , M. Mariscal‐Arcas , C. Monteagudo , B. Feriche , M. L. Lorenzo , F. Olea , Int. J. Food Sci. Nutr. 2013, 64, 155. 22946650 10.3109/09637486.2012.718743

[243] W. Wang , Y. Li , X. Meng , Heliyon 2023, 9, 12877. 10.1016/j.heliyon.2023.e12877 PMC9938420 36820164

[244] S. Cristiane , F. Mariane , R. Marcelo , S. Flavia , F. Regina , Cur. Dev. Nutr. 2022, 6, 1194.

[245] G. Voulgaridou , S. K. Papadopoulou , P. Detopoulou , D. Tsoumana , C. Giaginis , F. S. Kondyli , E. Lymperaki , A. Pritsa , Diseases 2023, 11, 29. 36810543 10.3390/diseases11010029 PMC9944083

[246] D. Haghmorad , A. Soltanmohammadi , M. Jadid Tavaf , S. Zargarani , E. Yazdanpanah , A. Shadab , B. Yousefi , Int. J. Neurosci. 2024, 134, 735. 36369838 10.1080/00207454.2022.2147431

[247] A. Popescu , M. German , Nutrients 2021, 13, 2206. 34199021 10.3390/nu13072206 PMC8308377

[248] Y. X. Yu , X. D. Yu , Q. Z. Cheng , L. Tang , M. Q. Shen , Aging 2020, 12, 16410. 32862152 10.18632/aging.103691 PMC7485738

[249] R. Lasemi , M. Kundi , N. B. Moghadam , H. Moshammer , J. A. Hainfellner , Wien. Klin. Wochenschr. 2018, 130, 307. 29500722 10.1007/s00508-018-1328-x PMC5966473

[250] J. Iwamoto , Y. Sato , K. Tanaka , T. Takeda , H. Matsumoto , Aging Clin. Exp. Res. 2009, 21, 277. 19959915 10.1007/BF03324916

[251] M. Montero‐Odasso , N. van der Velde , F. C. Martin , M. Petrovic , M. P. Tan , J. Ryg , S. Aguilar‐Navarro , N. B. Alexander , C. Becker , H. Blain , Age Ageing 2022, 51, 205.

[252] a) R. Bonanni , I. Cariati , U. Tarantino , G. D'Arcangelo , V. Tancredi , J. Funct. Morphol. Kinesiol. 2022, 7, 38; 35645300 10.3390/jfmk7020038 PMC9149968

[253] P. Z. Liu , R. Nusslock , Front. Neurosci. 2018, 12, 52. 29467613 10.3389/fnins.2018.00052 PMC5808288

[254] C. J. Cortes , Z. De Miguel , Brain Plast. 2022, 8, 865. 10.3233/BPL-220139 PMC9661359 36448044

[255] S. F. Sleiman , J. Henry , R. Al‐Haddad , L. El Hayek , E. Abou Haidar , T. Stringer , D. Ulja , S. S. Karuppagounder , E. B. Holson , R. R. Ratan , eLife 2016, 5, 15092. 10.7554/eLife.15092 PMC4915811 27253067

[256] B. K. Pedersen , Nat. Rev. Endocrinol. 2019, 15, 383. 30837717 10.1038/s41574-019-0174-x

[257] S. F. Kazim , J. Blanchard , C. L. Dai , Y. C. Tung , F. M. LaFerla , I. G. Iqbal , K. Iqbal , Neurobiol. Dis. 2014, 71, 110. 25046994 10.1016/j.nbd.2014.07.001

[258] R. E. MacPherson , Am. J. Physiol. Regul. Integr. Comp. Physiol. 2017, 313, R585. 28814391 10.1152/ajpregu.00255.2017 PMC5792152

[259] A. S. Puente‐Gonzalez , M. C. Sanchez‐Sanchez , E. J. Fernandez‐Rodriguez , J. E. Hernandez‐Xumet , F. J. Barbero‐Iglesias , R. Mendez‐Sanchez , Brain Sci. 2021, 11, 63. 33419016 10.3390/brainsci11010063 PMC7825330

[260] P. Müller , Dtsch. Z. Sportmed. 2020, 71, 113.

[261] R. Stephen , K. Hongisto , A. Solomon , E. Lönnroos , J. Gerontol., Ser. A 2017, 72, 733. 10.1093/gerona/glw251 28049634

[262] B. Intzandt , E. N. Beck , C. R. A. Silveira , Neurosci. Biobehav. Rev. 2018, 95, 136. 30291852 10.1016/j.neubiorev.2018.09.018

[263] L. Quinn , M. Busse , J. Carrier , N. Fritz , A. Rao , JBI Database System Rev. Implement. Rep. 2017, 15, 1783. 10.11124/JBISRIR-2016-003274 28708742

[264] M. R. Islam , S. Valaris , M. F. Young , E. B. Haley , R. Luo , S. F. Bond , S. Mazuera , R. R. Kitchen , B. J. Caldarone , L. E. Bettio , Nat. Metab. 2021, 3, 1058. 34417591 10.1038/s42255-021-00438-z PMC10317538

[265] P. Pignataro , M. Dicarlo , R. Zerlotin , C. Zecca , M. T. Dell'Abate , C. Buccoliero , G. Logroscino , S. Colucci , M. Grano , Int. J. Mol. Sci. 2021, 22, 1605. 33562601 10.3390/ijms22041605 PMC7915567

[266] C. E. Mazo , E. R. Miranda , J. Shadiow , M. Vesia , J. M. Haus , Brain Plast. 2022, 8, 5. 36448040 10.3233/BPL-220137 PMC9661358

[267] A. De la Rosa , E. Solana , R. Corpas , D. Bartrés‐Faz , M. Pallàs , J. Vina , C. Sanfeliu , M. C. Gomez‐Cabrera , Sci. Rep. 2019, 9, 3337. 30833610 10.1038/s41598-019-40040-8 PMC6399244

[268] A. Żebrowska , B. Hall , A. Maszczyk , R. Banaś , J. Urban , Diabetes Res. Clin. Pract. 2018, 144, 126. 30179684 10.1016/j.diabres.2018.08.018

[269] S. S. Jayashankar , K. T. Arifin , M. L. Nasaruddin , Nutrients 2023, 15, 524. 36771231 10.3390/nu15030524 PMC9921456

[270] L. El Hayek , M. Khalifeh , V. Zibara , R. Abi Assaad , N. Emmanuel , N. Karnib , R. El‐Ghandour , P. Nasrallah , M. Bilen , P. Ibrahim , J. Neurosci. 2019, 39, 2369. 30692222 10.1523/JNEUROSCI.1661-18.2019 PMC6435829

[271] M. Wang , H. Zhang , J. Liang , J. Huang , N. Chen , J. Neuroinflammation 2023, 20, 76. 36935511 10.1186/s12974-023-02753-6 PMC10026496

[272] C. Mo , A. J. Hannan , T. Renoir , Neurosci. Biobehav. Rev. 2015, 52, 178. 25770041 10.1016/j.neubiorev.2015.03.003

[273] M. Rai , F. Demontis , Brain Plast. 2022, 8, 43. 36448045 10.3233/BPL-210133 PMC9661353

[274] A. R. Isaac , R. A. S. Lima‐Filho , M. V. Lourenco , Neuropharmacology 2021, 197, 108744. 34363812 10.1016/j.neuropharm.2021.108744

[275] W. Dong , Y. Wang , S. Liao , M. Lai , L. Peng , G. Song , Microorganisms 2020, 8, 597. 32326047 10.3390/microorganisms8040597 PMC7232393

[276] W. Yang , Y. Liu , G. Yang , B. Meng , Z. Yi , G. Yang , M. Chen , P. Hou , H. Wang , X. Xu , Front. Cell Infect. Microbiol. 2021, 11, 712381. 34631598 10.3389/fcimb.2021.712381 PMC8498591

[277] L. Dohnalová , P. Lundgren , J. R. Carty , N. Goldstein , S. L. Wenski , P. Nanudorn , S. Thiengmag , K. P. Huang , L. Litichevskiy , H. C. Descamps , Nature 2022, 612, 739. 36517598 10.1038/s41586-022-05525-z PMC11162758

[278] H. Hamasaki , J. Exp. Integr. Med. 2017, 15, 270. 10.1016/S2095-4964(17)60342-X 28659231

[279] B. A. Dolezal , D. M. Boland , E. V. Neufeld , J. L. Martin , C. B. Cooper , Sports Med. 2019, 3, 48. 10.1055/a-0900-7501 PMC6629998 31312715

[280] W. Barton , N. C. Penney , O. Cronin , I. Garcia‐Perez , M. G. Molloy , E. Holmes , F. Shanahan , P. D. Cotter , O. O'Sullivan , Gut 2018, 67, 625. 28360096 10.1136/gutjnl-2016-313627

[281] M. Mokhtarzade , M. M. Shamsi , M. Abolhasani , B. Bakhshi , M. A. Sahraian , L. S. Quinn , R. Negaresh , Complement Ther. Clin. Pract. 2021, 45, 101463. 34348201 10.1016/j.ctcp.2021.101463

[282] C. Ohlsson , K. Sjögren , Trends Endocrinol. Metab. 2015, 26, 69. 25497348 10.1016/j.tem.2014.11.004

[283] A. Fasano , F. Bove , M. Gabrielli , M. Petracca , M. A. Zocco , E. Ragazzoni , F. Barbaro , C. Piano , S. Fortuna , A. Tortora , Mov. Disord. 2013, 28, 1241. 23712625 10.1002/mds.25522

[284] L. Rani , A. C. Mondal , Neurosci. Res. 2021, 168, 100. 33417973 10.1016/j.neures.2021.01.001

[285] S. Cankaya , B. Cankaya , U. Kilic , E. Kilic , B. Yulug , Drugs Context. 2019, 8, 212553. 30873213 10.7573/dic.212553 PMC6408180

[286] T. Wang , W. Kuang , W. Chen , W. Xu , L. Zhang , Y. Li , H. Li , Y. Peng , Y. Chen , B. Wang , Alzheimers Res. Ther. 2020, 12, 1. 10.1186/s13195-020-00678-3 PMC7489025 32928279

[287] Z. Yu , Y. Yang , R. B. Chan , M. Shi , T. Stewart , Y. Huang , Z. Liu , G. Lan , L. Sheng , C. Tian , CNS Neurosci. Ther. 2024, 30, 14393. 10.1111/cns.14393 PMC10848097 37563872

[288] H. R. Cheng , C. Y. Wen , C. Zhang , W. Kwapong , Authorea 2020.

[289] Y. L. Jiang , Z. X. Wang , X. X. Liu , M. D. Wan , Y. W. Liu , B. Jiao , X. X. Liao , Z. W. Luo , Y. Y. Wang , C. G. Hong , Adv. Sci. 2022, 9, 2105316.

[290] A. Obri , L. Khrimian , G. Karsenty , F. Oury , Nat. Rev. Endocrinol. 2018, 14, 174. 29376523 10.1038/nrendo.2017.181 PMC5958904

[291] M. Nakamura , M. Imaoka , M. Takeda , Int. J. Neurosci. 2021, 131, 1115. 32410480 10.1080/00207454.2020.1770247

[292] F. Oury , L. Khrimian , C. A. Denny , A. Gardin , A. Chamouni , N. Goeden , Y. Y. Huang , H. Lee , P. Srinivas , X. B. Gao , Cell 2013, 155, 228. 24074871 10.1016/j.cell.2013.08.042 PMC3864001

[293] X. Z. Guo , C. Shan , Y. F. Hou , G. Zhu , B. Tao , L. H. Sun , H. Y. Zhao , G. Ning , S. T. Li , J. M. Liu , Front. Mol. Neurosci. 2018, 11, 343. 30319352 10.3389/fnmol.2018.00343 PMC6170617

[294] V. Joers , M. G. Tansey , G. Mulas , A. R. Carta , Prog. Neurobiol. 2017, 155, 57. 27107797 10.1016/j.pneurobio.2016.04.006 PMC5073045

[295] Y. F. Hou , C. Shan , S. Y. Zhuang , Q. Q. Zhuang , A. Ghosh , K. C. Zhu , X. K. Kong , S. M. Wang , Y. L. Gong , Y. Y. Yang , Microbiome 2021, 9, 1. 33517890 10.1186/s40168-020-00988-6 PMC7849090

[296] S. Frank , Neurotherapeutics 2014, 11, 153. 24366610 10.1007/s13311-013-0244-z PMC3899480

[297] J. F. Cryan , K. J. O'Riordan , K. Sandhu , V. Peterson , T. G. Dinan , Lancet Neurol. 2020, 19, 179. 31753762 10.1016/S1474-4422(19)30356-4

[298] C. I. Wasser , E. C. Mercieca , G. Kong , A. J. Hannan , B. Allford , S. J. McKeown , J. C. Stout , Y. Glikmann‐Johnston , J. Huntington's Dis. 2023, 12, 43. 37005888 10.3233/JHD-220556

[299] L. Mazzini , L. Mogna , F. De Marchi , A. Amoruso , M. Pane , I. Aloisio , N. B. Cionci , F. Gaggìa , A. Lucenti , E. Bersano , J. Clin. Gastroenterol. 2018, 52, S68. 29782468 10.1097/MCG.0000000000001042

[300] a) J. Chen , N. Chia , K. R. Kalari , J. Z. Yao , M. Novotna , M. M. Paz Soldan , D. H. Luckey , E. V. Marietta , P. R. Jeraldo , X. Chen , Sci. Rep. 2016, 6, 1; 27346372 10.1038/srep28484 PMC4921909

